# Home range size in central chimpanzees (*Pan troglodytes troglodytes*) from Loango National Park, Gabon

**DOI:** 10.1007/s10329-021-00927-5

**Published:** 2021-07-04

**Authors:** Laura Martínez-Íñigo, Pauline Baas, Harmonie Klein, Simone Pika, Tobias Deschner

**Affiliations:** 1 Interim Group Primatology, Max Planck Institute for Evolutionary Anthropology, Deutscher Platz 6, 04103 Leipzig, Germany; 2 Institute of Cognitive Science, Comparative BioCognition, Osnabrück University, Artilleriestrasse 34, 49076 Osnabrück, Germany; 3 Wild Chimpanzee Foundation - Guinean Representation, Commune de Dixinn, BP1487P Conakry, Guinea

**Keywords:** Territory, BRB, Interspecies competition, Intercommunity competition, Habitat mosaic

## Abstract

Ranging behavior has been studied extensively in eastern (*Pan troglodytes schweinfurthii)* and western (*P. t. verus*) chimpanzees, but relatively little is known regarding home ranges of the other two subspecies (*P. t. ellioti; P. t. troglodytes*). In this study, we determined the home range size and space use of a habituated community (Rekambo) of central chimpanzees living in a habitat mosaic in Loango National Park, Gabon. Data on travel routes were collected during follows between January 2017 and April 2019 (*N* = 670,616 relocations, collected over 640 days and 5690 h of observation). We used three methods for calculating home range size (minimum convex polygon, kernel density estimation, and biased random bridges). We compare our estimates to those obtained from prior genetic and camera trap studies of the Rekambo community and contrast them with estimates from other chimpanzee communities of the four chimpanzee subspecies. Depending on the methodology used, the home range size of the Rekambo community ranged between 27.64 and 59.03 km^2^. The location of the center of the home range remained relatively stable over the last decade, while the overall size decreased. The Rekambo home range is, therefore, one of the largest documented so far for chimpanzees outside savannah-woodland habitats. We discuss several explanations, including the presence of savannah, interspecies competition, and intercommunity interactions.

**Supplementary Information:**

The online version contains supplementary material available at 10.1007/s10329-021-00927-5.

## Introduction

Animals usually restrict their activities to particular areas which may range from a few square meters to thousands of square kilometers (Burt 1943; Laver and Kelly 2008; Powell and Mitchell 2012). Such areas are called home ranges and are the specific geographic regions that an animal or a group of animals uses to meet its needs over a defined time span (Burt 1943). By comparing home range sizes across populations, it is possible to understand the ecological flexibility of species. Estimating home ranges sheds light on what habitats are essential for a species and how it might respond to environmental change (Mitani et al. 2010; Cumming and Cornélis 2012; Powell and Mitchell 2012; Fieberg and Börger 2012). As such, home range estimations are essential for understanding species-specific ecological requirements and implementing management and conservation programs (Fauvelle et al. 2017; Albani et al. 2020).

Chimpanzees (*Pan troglodytes*) are an endangered species with four subspecies: eastern (*P. t. schweinfurthii*), western (*P. t. verus*), Nigeria-Cameroon (*P. t. ellioti*), and central chimpanzees (*P. t. troglodytes*) (Humle et al. 2016). Chimpanzees live in communities comprising multiple adult males and females and their offspring (Nishida 1979; Goodall 1986; Boesch and Boesch-Achermann 2000; Watts and Mitani 2015). Across chimpanzee communities and subspecies, individuals spend most of their time in smaller parties (i.e., subgroups) of varying size and composition, termed fission–fusion societies (Nishida 1979; Goodall 1986; Boesch and Boesch-Achermann 2000; Watts and Mitani 2015). Activities are restricted to a home range whose location is relatively stable across the years, although its shape and size can vary relatively widely over time (Nakamura et al. 2013). Chimpanzees defend their home ranges aggressively and often lethally from neighboring communities (Mitani et al. 2010; Lemoine et al. 2020). Thus, chimpanzee home ranges are commonly called territories. They contain a heavily used central area (i.e., core area) surrounded by a less frequently visited periphery that may overlap extensively with neighboring territories (Herbinger et al. 2001; Wilson et al. 2001). Home range size in chimpanzees is related to the size of the community, food availability, population density, intercommunity relationships, and interspecific competition (Nishida et al. 1985; Goodall 1986; Boesch and Boesch-Achermann 2000; Herbinger et al. 2001; Lehmann and Boesch 2003; Amsler 2009; Mitani et al. 2010; Head et al. 2012; Nakamura et al. 2013; Lemoine et al. 2020).

Ranging patterns have been predominantly studied in eastern and western chimpanzees (see references above). However, relatively little is known about Nigeria-Cameroon chimpanzees and central chimpanzees (see for an overview Table S1 in Electronic Supplementary Material, ESM, and Abwe 2018). Central chimpanzees have been studied in a relatively homogenous forested environment (Morgan et al. 2006). Nevertheless, some communities, such as the Rekambo community in Loango National Park in Gabon, live in a mosaic of different habitats that contains savannahs and mangroves in addition to forests and swamps (Boesch et al. 2007). Loango National Park harbors a high density of two main food competitors, forest elephants (*Loxodonta cyclotis*) and western lowland gorillas (*Gorilla gorilla gorilla*) (Head et al. 2011, 2012, 2013).

Previous studies showed that the Rekambo community was surrounded by 3–5 other chimpanzee communities (Arandjelovic et al. 2011; Head et al. 2013). Before the habituation of the Rekambo community, their minimum home range was estimated as 45 km^2^, using noninvasive genetic monitoring (Arandjelovic et al. 2011), and 24.4 km^2^ using camera traps (Head et al. 2013). Preliminary direct observational data led to a home range estimate of 36 km^2^ for the community (Head et al. 2013). Here, we provide the first estimates of the home range of the Rekambo community based on data collected when most individuals were fully habituated to human presence, enabling direct and regular behavioral observations. We calculated annual and cumulative home ranges from January 2017 to April 2019, using three different estimators: minimum convex polygon, kernel density estimates and biased random bridges. Minimum convex polygon (Mohr 1947) and kernel density estimation (Worton 1987) have been commonly used to estimate chimpanzee home ranges (see Table S1 in ESM). Consequently, these estimators are useful for comparing estimates across chimpanzee sites. The third estimator, biased random bridges (Benhamou and Cornélis 2010), is more suited for the data that we have available than the more traditional estimators. We then compare our estimates to the minimum home range estimates of studies on the Rekambo community prior to their habituation (Arandjelovic et al. 2011; Head et al. 2013), as well as to estimates of other chimpanzee communities, and discuss the potential drivers of our results.

## Methods

### Study site and community

**Fig. 1:**
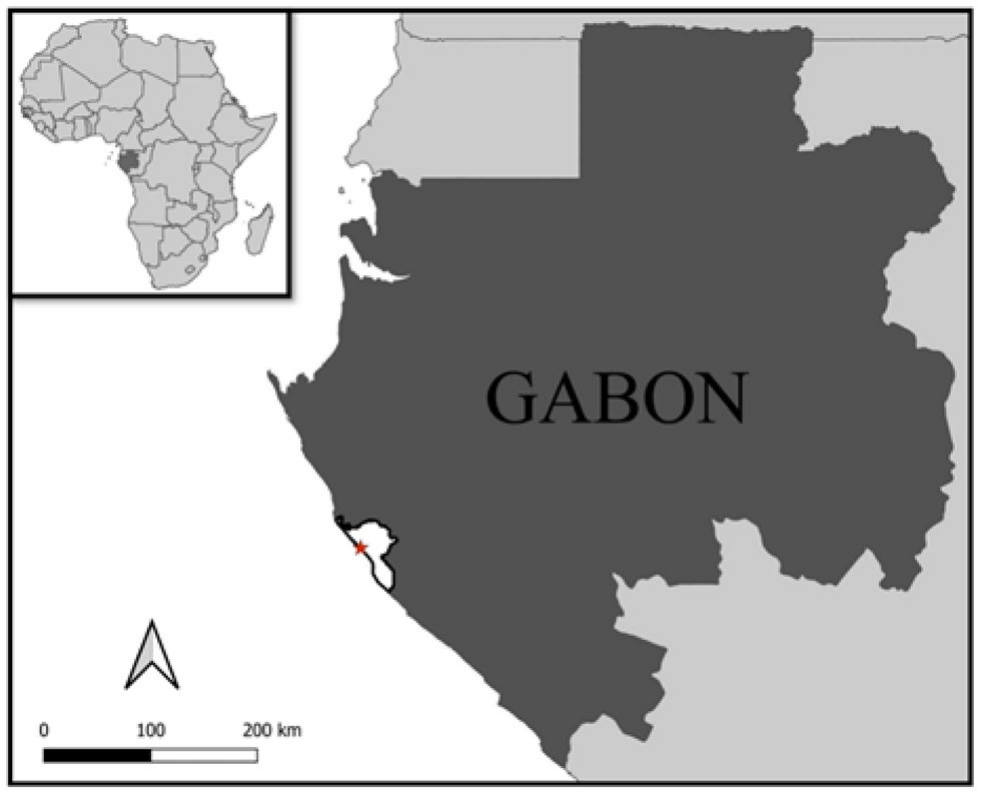
Location of study site (red star) in Loango National Park (white), Gabon (dark gray). Maps were created using QGIS (v.3.10.5, QGIS Development Team, 2019), and shapefiles from the Map Library (Map Maker Ltd 2007) and Protected Planet (UNEP-WCMC and IUCN 2020)

The study site was established in 2005 in Loango National Park, Gabon (see Fig. 1, 2° 04′ S, 9° 33′ E; Boesch et al. 2007). The area consists of a mosaic of rivers, swamps, coastal forests, mangroves, savannahs, and secondary and mature forests, bordered by the Atlantic Ocean and a lagoon (Boesch et al. 2007). The mean annual rainfall in 2017–2018 was 2099 mm. Temperatures ranged between 18 and 32 °C across the same period, with the mean minimum and maximum temperatures being 22.7 °C and 27.8 °C, respectively. There is a long rainy season between October and April, interrupted by a short dry season between December and January. The long dry season usually lasts from May to September (Head et al. 2011).

The size of the Rekambo community ranged from 44 to 47 individuals during the study period, including 16–17 adult females and 8–9 adult males (see Table S2 in ESM).

### Data collection and preparation

Between January 2017 and April 2019, observers followed parties of chimpanzees of the Rekambo community as soon as they encountered them each day. Similarly to all other communities studied so far, the entire community is rarely found together. Instead, individuals move alone, in a single party, or switch between several different parties over the course of a day. Observers followed the largest possible party of chimpanzees throughout the contact rather than particular individuals. Thus, the followed party may change size and composition throughout a given contact. Whenever possible, different teams of observers followed more than one party simultaneously. A contact started when observers saw at least one member of the community (contact starting time: median: 6:50, min: 6:00; max: 16:50). Observers stopped a contact before dusk or when no member of the community had been observed for more than 30 min (contact ending time: median: 17:55, min: 7:31; max: 18:55). Track-log data were collected every 1–60 s using a handheld GPS device (Garmin, Rhino 750) and downloaded into BaseCamp software (v. 4.7.1. Garmin Ltd. 2008). Whenever information from simultaneous contacts was available (i.e., traveling routes of different parties recorded over the same period by different teams of observers), only the longest duration travel route was retained for downstream analyses. This exclusion was necessary because the biased random bridges estimator cannot be computed with simultaneous routes belonging to the same individual or group. We applied the exclusion to the calculation of the three estimators to allow an easier comparison between their results.

### Home range analysis

We used a total of 670,616 relocations collected over 640 days and 5691 h of observation to calculate the home range of the Rekambo community from January 2017 to April 2019 (mean ± SD; 1126 ± 547 relocations/day). We calculated the home range using three estimators: minimum convex polygon (MCP), kernel density estimation (KDE), and biased random bridges (BRB) (for details see ESM).

MCPs are obtained by joining the outermost relocations to create a polygon with all internal angles not exceeding 180 degrees and encompassing all recorded locations. KDE calculates the probability of finding the target animal or group at any given time based on the frequency of having identified the target at the location and its nearby surroundings in the past (Powell and Mitchell 2012). BRB is an extension of KDE specifically designed to address movement data autocorrelation without the need for subsampling (Benhamou and Cornélis 2010). BRB incorporates information regarding the order in which locations are obtained, the time lag between them, as well as the average error made when locations are recorded (Benhamou and Cornélis 2010). The technique interpolates inferred locations in a straight line between each pair of consecutively recorded locations.

We computed and mapped the home ranges in R (v. 4.0.2*,* R Core Team 2020) using the package adehabitatHR (v0.4.18, Calenge 2006). We calculated home range size with MCP, KDE, and BRB using the isopleths 99%, 98%, and 95% to provide comparable estimations to results from other habituated chimpanzee communities (see Table S1 in ESM). We calculated the 100% isopleth only for MCP, which is the only method amenable to the procedure and has been widely used to calculate home range size in chimpanzees (see Table S1 in ESM). We estimated the core range area as 80%, 75%, and 50% isopleths for comparability with other chimpanzee studies (see Table S1 in ESM).

We calculated fixed KDE using the *h*_ref_ and *h*_LSCV_ techniques to select the smoothing parameter, since they have been used most often in other chimpanzee studies (Kouakou et al. 2011; Boyer Ontl 2017; Moore et al. 2018; Green et al. 2020a). However, our model did not converge with *h*_LSCV_. This is a frequent problem when the number of relocations is high (Walter et al. 2011; Pebsworth et al. 2012; Bauder et al. 2015; Boyer Ontl 2017; Moore et al. 2018). The value of *h*_ref_ for the cumulative home range (i.e. January 2017–April 2019) was 155.6736.

We used the package adehabitatLT (v.0.3.25, Calenge 2006) to store the travel routes into a *ltraj* object, which is necessary to calculate BRB in adehabitatHR. The values of the parameters needed to estimate BRB and their justification can be found in the ESM.

We calculated annual home ranges for 2017 and 2018 respectively, using the three techniques described above. Data for 2017 included 205,545 relocations collected for 259 days (856 ± 479 relocations/day) and 2328 h of observation. Data for 2018 included 340,263 relocations collected for 277 days (1326 ± 529 relocations/day) and 2793 h of observation. For KDE, *h*_ref_ of 2017 was 152.8558, and *h*_ref_ of 2018 was 172.765. All other parameters were the same for annual and cumulative estimates.

We used the package *caTools* (v.1.18.0, Tuszynski 2020) in R to calculate the area under the curve (AUC, Cumming and Cornélis 2012; Walter et al. 2015). AUC detects when a home range estimate includes areas in which there is no evidence of the presence of the target and excludes areas in which there is evidence of presence (Cumming and Cornélis 2012; Walter et al. 2015). Consequently, AUC serves as a goodness-of-fit metric, whose values range from 0.5 to 1. The closer AUC is to 1, the closer is the agreement between the estimated home range and the GPS relocations (Cumming and Cornélis 2012). We calculated AUC as in Cumming and Cornélis (2012, see R code in ESM); that is, we calculated one AUC per utilization density volume or MCP_100_ and not per isopleth. All estimates were made with grids of 100m × 100m to allow for comparable AUC calculations.

### Recalculating MCP and KDE under different relocation subsampling regimes

Commonly, chimpanzee home range size estimated by MCP and KDE use subsampling of 1–4 GPS relocations per observation day (Fawcett 2000; Newton-Fisher 2003; Morgan et al. 2006; Moore et al. 2018; Green et al. 2020a). Alternatively, subsampling relocations to one every 15–30 min is also employed (Herbinger et al. 2001; Williams et al. 2002; Amsler 2009; Kouakou et al. 2011; Wilson et al. 2012). Thus, to provide comparable estimates for the Rekambo community to other chimpanzee groups, we recalculated their home range between January 2017 and April 2019 under three subsampling regimes. We generated 30 subsets of data: ten subsets with one relocation per observation day (*N*_relocations_ = 640), ten subsets with three relocations per observation day (*N*_relocations_ = 1920), and ten subsets with twelve relocations per observation day (*N*_relocations_ = 7680; equivalent to 1–2 relocations per hour). Relocations were randomly selected per each observation day. We calculated MCPs (100% and 50%) and KDEs (95% and 50%), as described in the previous section, for each of the 30 subsets of data. We performed ANOVAs to investigate the differences between the average size of the home ranges estimated under the three subsampling schemes, depending on the technique and isopleth (e.g., we checked whether the average MCP_100_ was significantly different if calculated using a subset of 1 location/day, 3 locations/day, and 12 locations/day). We performed post hoc comparisons using Tukey’s honestly significant difference (HSD) test. *P*-values lower than 0.05 were considered significant.

## Results

The estimates of the Rekambo community home range size from January 2017 to April 2019 varied between 27.64 and 59.03 km^2^ depending on the estimator and isopleth considered (see isopleths 100–95% in Table 1). Home range size estimates of the same isopleth differed from 0.01 to 10.48 km^2^ between estimators. MCP included more areas without relocations than KDE (see AUC values in Table 1). KDE displayed slightly lower AUC than BRB. Relocation subsampling had a significant effect on the areas estimated. The lower the number of relocations, the lower the MCP estimate, while KDE estimates increased with fewer relocations (see Table 2).

**Table 1:** Home range areas (km^2^) by isopleth (%) and area under the curve (AUC) calculated for the Rekambo community by minimum convex polygon, kernel density estimation, and biased randombridges

	Estimator	Isopleth (%)							AUC
		100	99	98	95	80	75	50	
Cumulative	MCP	59.03	49.67	45.22	38.12	21.31	18.74	9.63	0.8823
	KDE	–	42.46	38.30	30.39	17.55	14.97	7.03	0.9990
	BRB	–	39.34	36.12	27.64	14.93	12.99	6.39	0.9995
2017	MCP	35.25	28.89	26.53	22.10	14.32	12.69	6.54	0.8653
	KDE	–	28.89	25.57	21.13	12.53	10.70	5.35	0.9334
	BRB	–	27.52	22.45	18.31	11.29	9.70	4.72	0.9977
2018	MCP	55.49	44.25	40.46	34.47	21.18	19.03	10.38	0.8599
	KDE	–	42.27	38.98	31.29	16.63	13.95	6.23	0.9955
	BRB	–	39.97	38.08	31.97	15.15	13.00	5.73	0.9988

Cumulative: January 2017–April 2019, 670,616 relocations collected over 640 days and 5691 h of observation. 2017: 205,545 relocations collected over 259 days and 2348 h of observation. 2018: 340,263 relocations were collected for 277 days and 2793 h of observation. AUC values can range from 0.5 to 1. The closer AUC values are to 1, the closer is the agreement between the estimated home range and the relocations (Cumming and Cornélis 2012). AUC are estimated once per full home range volume rather than per isopleth

**Table 2:** MCP_100_, MCP_50_, KDE_95,_ and KDE_50_ of the Rekambo community obtained with different relocation subsampling schemes

Relocations/day	km^2^			
	MCP_100_	MCP_50_	KDE_95_	KDE_50_
1	40.87 ± 1.91	8.03 ± 0.44	36.95 ± 0.75	9.2 ± 0.40
3	46.52 ± 1.66	8.62 ± 0.29	34.70 ± 0.17	8.56 ± 0.15
12	52.2 ± 1.77	8.81 ± 0.13	33.41 ± 0.23	8.02 ± 0.05

The total number of sampling days between January 2017 and April 2019 was 640. Each subsampling scheme was performed 10 times. Values are mean ± SD

### Home range analysis

The results showed that the Rekambo community home range and core area estimates varied depending on the estimator employed and the period considered (see Table 1). MCP consistently produced the largest estimates for all isopleths. KDE estimates were larger than BRB ones for the cumulative and 2018 estimates but not for 2017. MCP displayed the lowest AUC values, followed by KDE and BRB (see Table 1). Cumulative estimates, meaning estimates calculated combining all data between January 2017 and April 2019, were larger than 2017 estimates for all three methods. Cumulative estimates were larger than 2018 estimates for MCP and KDE, but the opposite was true for BRB (see Table 1).

**Fig. 2:**
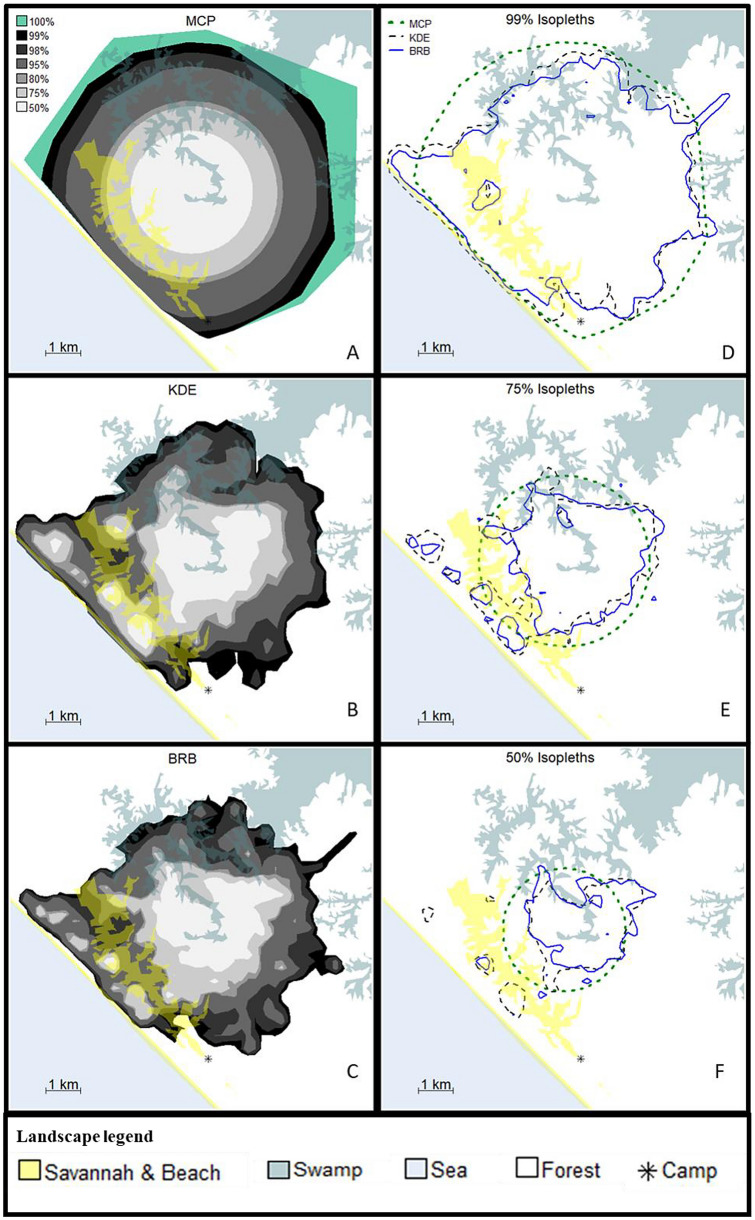
﻿Maps of the cumulative Rekambo home range calculated using three different techniques: minimum convex polygon (MCP), kernel density estimation (KDE), and biased random bridges (BRB). **A**–**C** show all the isopleths calculated with each technique in grayscale, where the darkest is 99% and the lightest 50%. The 100% MCP is depicted in turquoise. **D**–**F** compare equivalent isopleths of the three techniques. MCP in green, KDE in black, and BRB in blue. Background colors depict the different ecosystems: savannah, beach, swamp, sea, and forest. The * shows the location of the Ozouga research camp. Data used were 670,616 relocations collected over 640 days and 5691 h of observation between January 2017 and April 2019. The figure was created using R (v. 4.0.2, R Core Team 2020)

When focusing on the maximum common isopleth (see 99%, Fig. 2D), MCP_99_ encompasses KDE_99_ and BRB_99_, except for some areas close to the coast. MCP_99_ is unique in including the research camp within the Rekambo home range, while KDE_99_ overlays with the sea. Core area isopleths (75% and 50%) fell mostly in the center of the home range taking the 100%MCP as a reference. KDE and BRB displayed core area patches on the coastal forest (between the savannah and the sea, see Fig. 2E and F), but their areas were larger in KDE than in BRB. Several coastal forest patches that remain in KDE_50_ disappear in BRB_50_ (see Fig. 2F). The 50% isopleth of KDE and BRB is extended towards the edge of the swamp at the northeast of the center, outside the MCP_50_ (see Fig. 2F). In comparison, MCP_50_ encompassed parts of the savannah and left out the high-use patches from the coastal forest and the edge of the northeastern swamp.

### Recalculating MCP and KDE under different relocation subsampling regimes

Subsampling the relocations to obtain 1, 3, and 12 per observation day had significant effects on the size of the isopleths estimated by both MCP and KDE (MCP_100_: *F*(2,7) = 101.4, *p* < 0.001; MCP_50_: *F*(2,27) = 17.13; *p* < 0.001; KDE_95_: *F*(2,27) = 147.8, *p* < 0.001; KDE_50_: *F*(2,27) = 56.74, *p* < 0.001). The larger the number of relocations per observation day, the larger the area estimated by MCP isopleths, except the core area estimated with 3 and 12 relocations per day, which were not significantly different from each other (see Table 2, all Tukey tests *p* < 0.001, except for MCP_100(3relocations/day)_—MCP_100(12relocations/day),_ where *p* = 0.35). Conversely, the smaller the number of relocations per observation day, the larger the area estimated by KDE isopleths (see Table 2, Tukey tests *p* < 0.001). The difference between the cumulative MCP_100_ calculated with all data (see Table 1) and the MCP_100_ estimated with one relocation per observation day, (see Table 2) was 18.16 km^2^. The difference between the cumulative KDE_95_ calculated with all data (see Table 1), and the KDE_95_ estimated with one relocation per observation day (see Table 2), was 6.56 km^2^.

## Discussion

Here, we used three different estimators and different sampling procedures to assess the home range size of a community of central chimpanzees living in a mosaic of different habitat types. We provide the first home range size assessment of the Rekambo community after the habituation of most of its members. Overall, our data showed that the estimated home range size of Rekambo between January 2017 and April 2019 (i.e., cumulative estimate) was 39.34–49.67 km^2^ when focusing on the largest common isopleth of the three estimators (i.e., 99%). The annual estimates tended to be smaller than the cumulative estimate. The evaluation of the estimators indicated that BRB performed slightly better than KDE, and both were more accurate than MCP as measured by AUC. The subsampling of relocations yielded significant differences in the results, with smaller numbers of relocations per day leading to smaller MCP and larger KDE estimates.

### Comparison between different home range estimators

The Rekambo home range size estimates differed as expected between those produced with MCP, KDE, and BRB (Börger et al. 2006; Amsler 2009). Equivalent isopleths of MCP covered larger areas than those of KDE and BRB (see Table 1). As expected, subsampling significantly affected the areas estimated by MCP and KDE (Pebsworth et al. 2012; Fieberg and Börger 2012). Smaller numbers of relocations per day produced smaller MCP estimates and larger KDE estimates. KDE and BRB fit the data with comparable accuracy according to AUC values. However, BRB tended to minimally outperform KDE (see Table 1).

Our findings thus strengthen recent notions that, currently, no existing home range estimator is suited for all practical situations and research questions (Fieberg and Börger 2012; Bauder et al. 2015). However, most studies would benefit from estimators that reasonably fit and are suitable for their data (Cumming and Cornélis 2012; Walter et al. 2015). Researchers investigating nonhuman primates often have access to large datasets of highly autocorrelated GPS data (Pebsworth et al. 2012; Cheyne et al. 2019; Albani et al. 2020; Dore et al. 2020). Using such a dataset, we showed that KDE and BRB produced estimates similar to each other in terms of isopleth area and shape (see Fig. 2, Table 1, and Figs. S2 and S3 in ESM). However, BRB showed a slightly better fit according to AUC in the three conditions tested (i.e., one cumulative and two annual estimations). BRB was developed for datasets like the one we had, with highly autocorrelated relocations (Benhamou and Cornélis 2010). Thus, it is not surprising that BRB estimates are closer to the observed relocations in this case. However, BRB presents a practical problem when it comes to between-population comparisons. BRB requires selecting more parameters than KDE, which are likely to differ between studies. Similar problems will arise with other new estimators that also account for the temporal component of relocations (e.g., Brownian bridge models, BBMM, Bullard 1998; Horne et al. 2007; dynamic Brownian bridge models, dBBMM, Kranstauber et al. 2012; or autocorrelated kernel density estimation, AKDE, Fleming et al. 2015; Calabrese et al. 2016; Noonan et al. 2019). This may render home range size comparisons between populations even more challenging than they usually are. We exemplify this aspect when comparing the Rekambo home range size to those of other chimpanzee communities. Decisions on what values to choose for the parameters should be made in line with the aims of each study, and it would be unrealistic to expect a consensus across researchers investigating the same species. Therefore, a potential solution to enable comparisons across populations could be to make GPS-relocation information available in databanks, such as movebank.org (Wikelski et al. 2020), allowing researchers to standardize home ranges calculations to compare across sites (Gregory 2017). Researchers specialized in the study of mammal orders such as Rodentia, Carnivora, Cingulata, or Artiodactyla (e.g., Rowcliffe et al. 2012; Calabrese et al. 2016) frequently deposit their GPS-relocation data in databanks. However, this practice is currently uncommon among primatologists (but see Strandburg-Peshkin et al. 2016). Decisions on the availability of location data, however, should consider the potential impacts for the study population (e.g., facilitating poaching).

### Comparison with previous estimates of the minimum home range of the Rekambo community

Previous studies have estimated the minimum home range of the Rekambo community before habituation. For instance, Arandjelovic and colleagues (2011) estimated a minimum home range of 45 km^2^ for the Rekambo community between 2005 and 2008 using noninvasive genetic monitoring. Head and colleagues (2013), using camera trap data collected for 20 months between 2009 and 2010, estimated a minimum home range of 24.4 km^2^ for the Rekambo community. Subsequently, during habituation efforts between 2009 and 2011, Head and colleagues (2013) calculated an MCP_100_ of 36 km^2^ for this community based on direct observations.

All previous estimates of the minimum home range of the Rekambo community (Arandjelovic et al., 2011; Head et al. 2013) were smaller than our cumulative estimate (i.e.,${\text{MCP}}_{{100}}^{{2017 - 2019}}$: 59.03 km^2^). However, we argue that the actual home range of the community was larger in previous years than in our study period.

First, rarely used areas are unlikely to be represented in an MCP_100_ drawn using data from noninvasive genetic monitoring due to the low probability of finding samples in areas that are not frequently visited by the study subjects (Granjon et al. 2017; Arandjelovic and Vigilant 2018). In contrast, our ${\text{MCP}}_{{100}}^{{2017 - 2019}}$ included one-time forays, which significantly increased the total estimate (e.g., compare ${\text{MCP}}_{{100}}^{{2017 - 2019}}$ and ${\text{MCP}}_{{99}}^{{2017 - 2019}}$ in Table 1 and Fig. 2A). Second, Arandjelovic and colleagues (2011) found samples belonging to the Rekambo community males 1–2 km to the north and south of the limits of our ${\text{MCP}}_{{100}}^{{2017 - 2019}}$. This finding indicates that the home range was larger in 2005–2008 than during our study period.

Third, the study of Head and colleagues (2013) did not monitor some of the northernmost areas that the Rekambo community used in 2005–2008 (Arandjelovic et al. 2011) and during our study period. Chimpanzee community members may, however, have used those areas where no cameras were placed, as they did before and after the study. If this is true, the home range of 2009–2010 would be larger than estimated. Finally, the number of relocations collected during follows greatly exceeds the quantity of equivalent data used by Head and colleagues (2013; 840 GPS points in their study versus 670,616 in ours). Because MCPs_100_ are very sensitive to sample size (e.g., see Table 2), the difference in data quantity is the most likely explanation for the smaller home range size estimate for 2009–2011 in comparison to our 2017–2019 estimate.

Further evidence suggesting that the Rekambo home range might have been larger in the past is the observation of fewer males during our study period. Studies at other sites such as Gombe (Tanzania) and Taï (Côte d'Ivoire) showed that home range size is often positively correlated with the number of males in the community (Goodall 1986; Boesch and Boesch-Achermann 2000; Lehmann and Boesch 2003). In 2005–2008, Arandjelovic and colleagues (2011) genotyped a minimum of 21 weaned males in the Rekambo community, while Head and colleagues (2013) assigned at least 16 weaned males using camera trap data from 2009 to 2010. These estimates are higher than the 14–16 weaned males observed in our study period (Martínez-Íñigo et al. 2021). Thus, the Rekambo community may have experienced a home range size reduction over the years, along with a decrease in the number of adult males in the community.

### Comparing the Rekambo home range size to the home range of other chimpanzee communities

Most chimpanzee studies have relied on either MCP or KDE to calculate the home range of their community (see Table S1 in ESM). However, studies vary in the amount of data used, the time frame included, how presence data were collected, whether deep incursions were excluded or, in the case of KDE, in the parameters used (see Table S1 in ESM for references). Home range size estimates can change an order of magnitude or more, depending on the estimator employed, the time frame considered, and the number of relocations per day used to produce the estimation (see Tables 1, 2; Grueter et al. 2009; Pebsworth et al. 2012). Thus, it is essential to keep these limitations in mind when comparing home range sizes across studies and communities.

The Rekambo community, which inhabits a forest mosaic, shows a home range size intermediate between forest chimpanzee sites and savannah sites (see Table S1 in ESM for a home range size comparison across 34 chimpanzee communities). We argue that the large size of the home range is likely produced by a combination of factors: the presence of savannah within the home range, interspecific competition, and intercommunity interactions.

Looking at the maximum estimates for each chimpanzee community (see Table S1 in ESM), the Rekambo community has the largest home range after Ugalla (Tanzania, 400–500 km^2^), Mt. Assirik (Senegal, 278–333 km^2^), Kasakati L (Tanzania, 124 km^2^), Kasakati Z (Tanzania, 122 km^2^), Fongoli (Senegal, 110.39 km^2^), and Mayebe (Rwanda; 60.98 km^2^) (see Table S1 in ESM for references). Mayebe dwells in a montane forest, a habitat which tends to have lower food availability than lowland forests (Green et al. 2020b). All other communities inhabit dry habitats. Dry habitats tend to have low food availability (Dunbar 1988; Janson and Chapman 1999; Hunt and McGrew 2002) and low population density (Wilson et al. 2014), and are both associated with large home range sizes (Maruhashi 1998; South 1999; Campos et al. 2014). However, Loango has one of the highest annual rainfalls registered for chimpanzee study sites, including those in rainforests (Wessling et al. 2018). Therefore, habitat dryness is unlikely to be the reason why the Rekambo home range is larger than those reported for other forest-dwelling communities of similar size such as Sonso (6.78–9.68 km^2^, 38–56 individuals) or Kanyawara (37.8–41.4 km^2^; 43–51 individuals) both from Uganda (see Table S1 in ESM for references).

The large home range size of the Rekambo community may be due to the National Park's unique habitat mosaic which includes savannah, a habitat that is poor in food resources for chimpanzees. The Rekambo chimpanzees heavily use the bordering areas of the savannah patches in specific periods (see Fig. 2), such as the *Sacoglottis gabonensis* fruiting season. However, the savannah itself only provides relatively low amounts of other fruit sources, such as *Chrysobalanus icaco* (Loango Chimpanzee Project, unpublished data). Thus, the community mostly uses the savannah to traverse between the mature forest and the coastal forest. Hence, although the savannah adds a crucial part to the overall home range size, it is not an important contributor to chimpanzees' food resources. It, thereby, may reduce the average food density per square kilometer, contributing to the large size of the home range and the low population density.

A second factor contributing to the large home range size of the Rekambo community is intense interspecific competition for food resources. Loango National Park shelters forest elephants and western lowland gorillas in addition to chimpanzees. Their diets overlap considerably in Loango (Head et al. 2012), as they do in other central African sites such as Kahuzi-Biega, in the Democratic Republic of the Congo (Kaboko community in Table S1 in ESM), and Goualougo Triangle in Nouabale-Ndoki National Park, Republic of the Congo (Moto community in Table S1 in ESM) (Yumoto et al. 1995; Blake 2002; Morgan and Sanz 2006). Elephants and gorillas could be lowering the density of food available to chimpanzees, resulting in an increase of the home range size of the Rekambo community. However, so far, relatively little is known about interactions and food competition of the Rekambo chimpanzees with gorillas and elephants. Head and colleagues (2011) found that chimpanzee and gorilla diets at Loango overlapped between 0.3 and 69% in relation to the season but not to fruit availability. Southern and colleagues (2021, in press) recently reported two lethal coalitional attacks of individuals of the Rekambo community against gorillas. They argued that additional observations in combination with isochronous assessments of fruit availability and dietary overlap are crucially needed to differentiate whether the attacks represent opportunistic hunting or species competition in times of food scarcity. In addition, elephants competitively exclude chimpanzees when fruits are scarce (Head et al. 2012) and compete with them for honey (Estienne et al. 2017a). Elephants are more abundant in Loango National Park than in either Kahuzi-Biega or Nouabale-Ndoki National Park (Hall et al. 1997; Stokes et al. 2010; Head et al. 2012). Thus, interspecies competition between elephants and chimpanzees may also have a crucial impact upon home range size at Loango.

Finally, intercommunity interactions might be a third factor contributing to the size of the Rekambo community home range. At Taï, low intercommunity encounter rates correlate with larger home ranges (Boesch and Boesch-Achermann 2000; Lehmann and Boesch 2003; Lemoine et al. 2020). Both, low intercommunity encounter rates and large home ranges, seem to be a consequence of a large community size relative to neighboring communities (Lemoine et al. 2020). Previous studies estimated that at least three chimpanzee communities surrounded Rekambo, which was the largest community of the study area in 2005–2011 (Arandjelovic et al. 2011; Head et al. 2013). To date, individuals of the Rekambo community encounter individuals of neighboring communities less often than many other chimpanzee communities elsewhere (Martínez-Íñigo et al. 2021). The combination of large home range and community size, and low intergroup encounter rate, suggests that Rekambo benefits from a competitive advantage over neighboring communities. In fact, combined evidence suggests that the Rekambo community expanded their range towards the south, beyond the research camp (see Fig. 2), after killing several individuals of the community that ranged there between 2005 and 2007 (Boesch et al. 2007; Arandjelovic et al. 2011). Consequently, a competitive advantage over their neighboring communities would be a factor explaining the Rekambo community’s large home range size. Nonetheless, contrary to this interpretation, neighboring communities seem to exert great pressure over Rekambo, entering within their core area, where intergroup encounters are more frequent than in the periphery (Martínez-Íñigo et al. 2021). At Taï, communities experiencing incursions into their core areas are less numerous than their neighbors, and their home ranges are smaller (Lemoine et al. 2020). Hence, it may be possible that the Rekambo community had a numerical advantage that allowed them to maintain a large home range in the past (i.e., 2005–2011). However, between 2005 and 2019, the number of adult males, which are the most active age-sex class during intercommunity encounters (Martínez-Íñigo et al. 2021), decreased in the Rekambo community (Arandjelovic et al. 2011; Head et al. 2013; Estienne et al. 2017b, present study). As a consequence, the community might have lost their competitive advantage (Boesch et al. 2007; Arandjelovic et al. 2011), encouraging neighbors to enter the home range of the Rekambo community. If this is the case, we may expect a shrinkage in the size of the home range of the Rekambo community along with an increased rate of intercommunity encounters in the future.

## Conclusions and future research

Currently, there are many different home range estimators available to researchers. MCP and KDE have long been used, and are useful for comparison across studies. However, researchers compiling highly autocorrelated data from GPS devices would obtain more accurate estimates using new-generation estimators designed explicitly for such data, like BRB.

The Rekambo community appears to have one of the largest home ranges among chimpanzees living in habitats other than savannah-woodland, regardless of the estimator or amount of data used to calculate it. The location of its home range has remained constant over the last decade, although some evidence suggests that its size might have decreased. The large size of the home range could be due to several factors combined, such as the presence of a considerable amount of savannah habitat within the range, interspecies competition with elephants and gorillas, and intercommunity relationships. Future studies on food availability and distribution at the site, as well as further research into interspecies competition between sympatric apes and other large mammals, are crucial to understanding their impact on home range size.

## Supplementary Information

Below is the link to the electronic supplementary material.

Supplementary file1 (DOCX 4161 KB)

## Data Availability

Travel routes are available through Movebank (https://www.movebank.org/cms/webapp?gwt_fragment=page=studies,path=study1280077382; Movebank ID: 1280077382).

## Code availability

Electronic Supplementary Material.
