# Supplementary material for: Home range size in central chimpanzees (Pan troglodytes troglodytes) from Loango National Park, Gabon
Source: Primates. 2021 Jul 4;62(5):723–34. doi: 10.1007/s10329-021-00927-5 (PMC8410711; doi:10.1007/s10329-021-00927-5)
Supplement: Supplementary file 1 — Supplementary file1 (DOCX 4161 KB) [file 10329_2021_927_MOESM1_ESM.docx]

**Supplementary information**

**Home range size in central chimpanzees (*Pan troglodytes troglodytes*) from Loango National Park, Gabon**

**Journal:** Primates

Laura Martínez-Íñigo, Pauline Baas, Harmonie Klein, Simone Pika, Tobias Deschner

***Corresponding author:***

Laura Martínez-Íñigo

Email: laura_m_innigo@live.com

**SUPPLEMENTS**

## Extended methods of home range analysis

We used 670,616 relocations collected over 640 days and 5,690 hours of observation to calculate the home range of the Rekambo community from January 2017-April 2019 (Mean±SD; 1,126.4±546.62 relocations/day). We calculated the home range using three estimators: Minimum Convex Polygon (MCP), Kernel Density Estimation (KDE), and Biased Random Bridges (BRB).

Over the last decades, MCP and KDE have been the most widely used estimators in wildlife ecology in general (Laver and Kelly 2008; Fauvelle et al. 2017) and chimpanzee studies in particular (see Table S1 in ESM). MCPs are obtained by joining the outermost relocations to create a polygon with all internal angles not exceeding 180 degrees and encompassing all recorded locations (Lay 1942; Mohr 1947; Worton 1987) or a pre-set percentage of them (Worton 1995). MCPs are simple to calculate, even with few data points, but often include unused areas, are strongly sensitive to sample size and outliers, and do not provide information on how intensely each part of the home range is used (Gregory 2017).

KDE calculates the probability of finding the target animal or group at any given time based on the frequency of having identified the target at the location and its nearby surroundings in the past (Powell and Mitchell 2012). A smoothing parameter or bandwidth (h) defines how close a sampling event must occur to a location for it to be considered nearby. In turn, this has a significant effect on the size of the home range estimated (Börger et al. 2006; Walter et al. 2011; Bauder et al. 2015). KDE home ranges encompass fewer unvisited areas than MCP home ranges and allow detecting several hotspots of activity instead of assuming a central core area (Börger et al. 2006). However, KDE also tends to overestimate home range area, especially with few relocations (Börger et al. 2006). Furthermore, the KDE models assume that data are independent (Worton 1989). Movement data are inherently autocorrelated and therefore dependent but can be treated as independent if sufficiently separated in time (Swihart and Slade 1985). Thus, researchers often subsample their relocations to attain the required statistical independence for KDE (Fawcett 2000; Morgan et al. 2006; Moore et al. 2018; Green et al. 2020a).

Subsampling, however, comes with the cost of losing biologically meaningful information (de Solla et al. 2001; Walter et al. 2011). Hence, some researchers opt to use all available data and possibly obtain biased estimates (Swihart and Slade 1985; Noonan et al. 2019 but see de Solla et al. 2001; Walter et al. 2011). BRB is an extension of KDE specifically designed to address movement data autocorrelation without the need for subsampling (Benhamou and Cornélis 2010). BRB incorporates information regarding the order in which locations are obtained, the time lag between them, and the average error made when locations are recorded (Benhamou and Cornélis 2010). The technique interpolates inferred locations in a straight line between each pair of consecutively recorded locations. Then, the technique applies the kernel function over each recorded and inferred location. However, the smoothing parameter does not remain constant, as in the traditional KDE. Instead, it varies between a minimum and a maximum value. The uncertainty of the animal's true location is minimal in the recorded locations, where the sole source of variability is the measurement error. Thus, the minimum value of the smoothing parameter is applied over the recorded locations. The highest uncertainty of the animal's location is the inferred location in the midpoint between two recorded locations. Thus, the smoothing parameter reaches its maximum value in these points. Inferred relocations between locations and mid-points have values in between the maximum and the minimum. BRB discards more unused areas than KDE (Walter et al. 2015) and is robust to missing data (Stark et al. 2017) but have not yet been frequently used in primatology (but see Campos et al. 2014; Stark et al. 2017; Albani et al. 2020).

We computed and mapped the home ranges in R (v. 4.0.2, R Core Team 2020) using the package adehabitatHR (v0.4.18, Calenge 2006). We calculated home range size with MCP, KDE, and BRB using the isopleths 99%, 98%, and 95% to provide comparable estimates to results from other habituated chimpanzee communities (see Table S1 in ESM). We calculated the 100% isopleth only for MCP, which is the only method amenable to the procedure and has been widely used to calculate home range size in chimpanzees (see Table S1 in ESM). We estimated the core range area as 80%, 75%, and 50% isopleths for comparability with other chimpanzee studies (see Table S1 in ESM).

We calculated fixed KDE using the h_ref_ and h_LSCV_ techniques to select the smoothing parameter (Börger et al. 2006; Fieberg 2007) since they have both been the most commonly used in chimpanzee studies (Kouakou et al., 2011; Boyer Ontl, 2017; Moore et al., 2018; Green et al., 2020a). However, our model did not converge with h_LSCV_. This is a frequent problem when the number of relocations is high (Walter et al. 2011; Pebsworth et al. 2012; Bauder et al. 2015; Boyer Ontl 2017; Moore et al. 2018). The value of h_ref_ for the cumulative home range (i.e., January 2017-April 2019) was 155.6736.

We used the package adehabitatLT (v.0.3.25, Calenge 2006) to store the travel routes into a *ltraj* object, which is necessary to calculate BRB in adehabitatHR. The parameters needed to estimate BRB are the diffusion coefficient (D), maximum time threshold (T_max_), minimum step length (L_min_), and minimum kernel smoothing parameter (h_min_, Calenge 2006). D is the speed of random drift between relocations. T_max_ is the maximum time above which successive relocations are not considered linked. L_min_ is the smallest distance below which the animal or group is considered to be still. H_min_ is the minimum smoothing parameter applied to the relocations. H_min_ is meant to account for several sources of location uncertainty, such as the standard deviation of the localization errors and random components of focal animal’s or group’s behaviour (Benhamou and Cornélis 2010). We calculated D using the *BRB.likD* function of the R package adehabitatHR, searching on a range of 0.1-10000. The resulting D was 1.6 m^2^/s. We first set T_max_ as 30 minutes since this was the amount of time we used to define separate chimpanzee contacts (see Fig.S1 in ESM). However, this short period produced an under-representation of core areas around the mangroves, in which observers tended to lose the chimpanzees due to the rugged terrain. For these occasions, chimpanzees were often re-found within the next two hours, not far from the initial obstacle. Thus, we set Tmax as 120 minutes to account for these incidents. We set Lmin as 50 m to account for the GPS reading error plus observers keeping a distance from the chimpanzees and moving around to observe them. We set h_min_ as 100 m as a rough estimate of all the sources of uncertainty, including the chimpanzee party spread.

We calculated annual home ranges for 2017 and 2018, respectively, using the three techniques described above. Data for 2017 included 205,545 relocations collected for 259 days and 2,328 hours of observation. Data for 2018 included 340,263 relocations collected for 277 days and 2,793 hours of observation. For KDE, 2017 h_ref_ of 2017 was 152.8558, and 2018 h_ref_ of 2018 was 172.765. All other parameters were the same for annual and cumulative estimates.

We used the package *caTools* (v.1.18.0, Tuszynski 2020) in R to calculate the area-under-the-curve (AUC, Cumming and Cornélis 2012; Walter et al. 2015). AUC detects when a home range estimate includes areas in which there is no evidence of the presence of the target and excludes areas in which there is evidence of presence (Cumming and Cornélis 2012; Walter et al. 2015). Consequently, AUC serves as a metric of goodness-of-fit, whose values range from 0.5 to 1. The closer AUC is to 1, the closer is the agreement between the estimated home range and the GPS relocations (Cumming and Cornélis 2012). We calculated AUC as in Cumming and Cornélis (2012, see R code in ESM); that is, we calculated one AUC per utilization density volume or MCP100 and not per isopleth. All estimates were made with grids of 100mX100m to allow for comparable AUC calculations among them.

## References from extended methods of home range analysis

Albani A, Cutini M, Germani L, et al (2020) Activity budget, home range, and habitat use of moor macaques (*Macaca maura*) in the karst forest of South Sulawesi, Indonesia. Primates. 61: 673-684. https://doi.org/10.1007/s10329-020-00811-8

Bauder JM, Breininger DR, Bolt MR, et al (2015) The role of the bandwidth matrix in influencing kernel home range estimates for snakes using VHF telemetry data. Wildl Res 42:437–453. https://doi.org/10.1071/WR14233

Benhamou S, Cornélis D (2010) Incorporating movement behavior and barriers to improve kernel home range space use estimates. J Wildl Manage 74:1353–1360. https://doi.org/10.2193/2009-441

Börger L, Franconi N, De Michele G, et al (2006) Effects of sampling regime on the mean and variance of home range size estimates. J Anim Ecol 75:1393–1405. https://doi.org/10.1111/j.1365-2656.2006.01164.x

Boyer Ontl KM (2017) Chimpanzees in the Island of Gold: Impacts of artisanal small-scale gold mining on chimpanzees (*Pan troglodytes verus*) in Fongoli, Senegal. Dissertation, Iowa State University

Calenge C (2006) The package "adehabitat" for the R software: A tool for the analysis of space and habitat use by animals. Ecol Modell 197:516–519. https://doi.org/10.1016/j.ecolmodel.2006.03.017

Campos FA, Bergstrom ML, Childers A, et al (2014) Drivers of home range characteristics across spatiotemporal scales in a Neotropical primate, *Cebus capucinus*. Anim Behav 91:93–109. https://doi.org/10.1016/j.anbehav.2014.03.007

Cumming GS, Cornélis D (2012) Quantitative comparison and selection of home range metrics for telemetry data. Divers Distrib 18:1057–1065. https://doi.org/10.1111/j.1472-4642.2012.00908.x

de Solla DE, Shane R, Bonduriansky R, Brooks RJ (2001) Eliminating autocorrelation reduces biological relevance of home range estimates. J Anim Ecol 68:221–234

Fauvelle C, Diepstraten R, Jessen T (2017) A meta-analysis of home range studies in the context of trophic levels: Implications for policy-based conservation. PLoS One 12(3): e0173361. https://doi.org/10.1371/journal.pone.0173361

Fawcett KA (2000) Female relationships and food availability in a forest community of chimpanzees. Dissertation, University of Edinburgh

Fieberg J, Börger L (2012) Could you please phrase "home range" as a question? J Mammal 93:890–902. https://doi.org/10.1644/11-mamm-s-172.1

Green SJ, Boruff BJ, Grueter CC (2020a) From ridge tops to ravines: landscape drivers of chimpanzee ranging patterns. Anim Behav 163:51–60. https://doi.org/10.1016/j.anbehav.2020.02.016

Gregory T (2017) Home Range Estimation. In: Fuentes A (ed) The International Encyclopedia of Primatology. John Wiley & Sons, Inc., Hoboken, NJ, pp 1–4

Kouakou CY, Boesch C, Kuehl HS (2011) Identifying hotspots of chimpanzee group activity from transect surveys in Taï National Park, Côte d'Ivoire. J Trop Ecol 27:621–630. https://doi.org/10.1017/S0266467411000423

Laver PN, Kelly MJ (2008) A Critical Review of Home Range Studies. J Wildl Manage 72:290–298. https://doi.org/10.2193/2005-589

Lay DW (1942) Ecology of the opossum in eastern Texas. J Mammal 23:147–159

Mohr CO (1947) Table of equivalent populations of North American small mammals. Am Midl Nat 37:223–249

Moore JF, Mulindahabi F, Gatorano G, et al (2018) Shifting through the forest: home range, movement patterns, and diet of the eastern chimpanzee (*Pan troglodytes schweinfurthii*) in Nyungwe National Park, Rwanda. Am J Primatol 80 (8): e22897. https://doi.org/10.1002/ajp.22897

Morgan D, Sanz C, Onononga JR, Strindberg S (2006) Ape abundance and habitat use in the Goualougo Triangle, Republic of Congo. Int J Primatol, 27(1):147-149. https://doi.org/10.1007/s10764-005-9013-0

Pebsworth PA, Morgan HR, Huffman MA (2012) Evaluating home range techniques: Use of Global Positioning System (GPS) collar data from chacma baboons. Primates 53:345–355. https://doi.org/10.1007/s10329-012-0307-5

Powell RA, Mitchell MS (2012) What is a home range? J Mammal 93:948–958. https://doi.org/10.1644/11-mamm-s-177.1

R Core Team (2020) R: A language and environment for statistical computing. R Foundation for Statistical Computing, Vienna

Swihart RK, Slade NA (1985) Influence of sampling interval on estimates of home range size. J Wildl Manage 49:1019–1025.

Tuszynski J (2020) caTools: Tools: Moving Window Statistics, GIF, Base64, ROC AUC, etc. R package version 1.18.0

Walter DW, Fischer JW, Baruch-Mordo S, VerCauteren KC (2011) What is the proper method to delineate home range of an animal using 'today's advanced GPS telemetry systems: the initial step. In: Krejcar O (ed) Modern Telemetry. InTech, London, pp 249–268

Walter WD, Onorato DP, Fischer JW (2015) Is there a single best estimator? Selection of home range estimators using area-under-the-curve. Mov Ecol 3:1–11. https://doi.org/10.1186/s40462-015-0039-4

Worton BJ (1987) A review of models of home range for animal movement. Ecol Modell 38:277–298. https://doi.org/10.1016/0304-3800(87)90101-3

Worton BJ (1995) A convex hull-based estimator of home-range size. Biometrics 51:1206–1215

Worton BJJ (1989) Kernel methods for estimating the utilization distribution in home-range studies


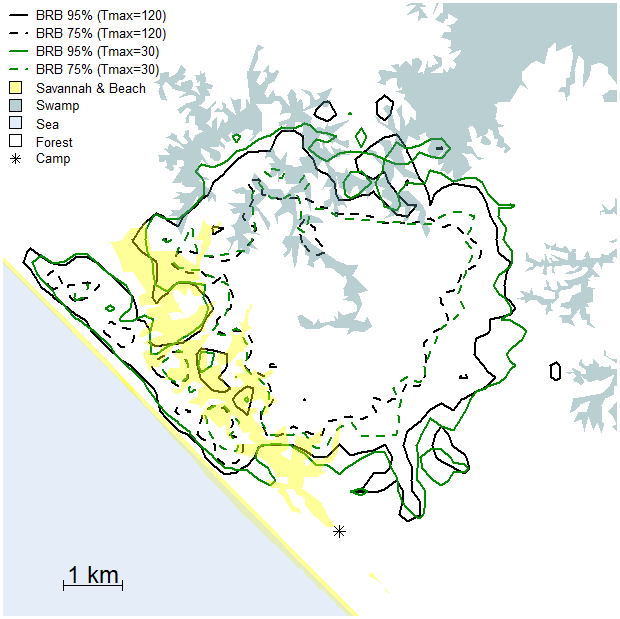


Fig. S1 Rekambo home range and core areas (January 2017-April 2019) calculated using Biased Random Bridges with maximum time threshold (T_max_) of 120 and 30 minutes, respectively. When calculated with T_max_=30 min, BRB 95%= 29.43 km^2^ and BRB 75% = 13.10 km^2^. Maps created with R (v.4.0.2 R Core Team 2020).


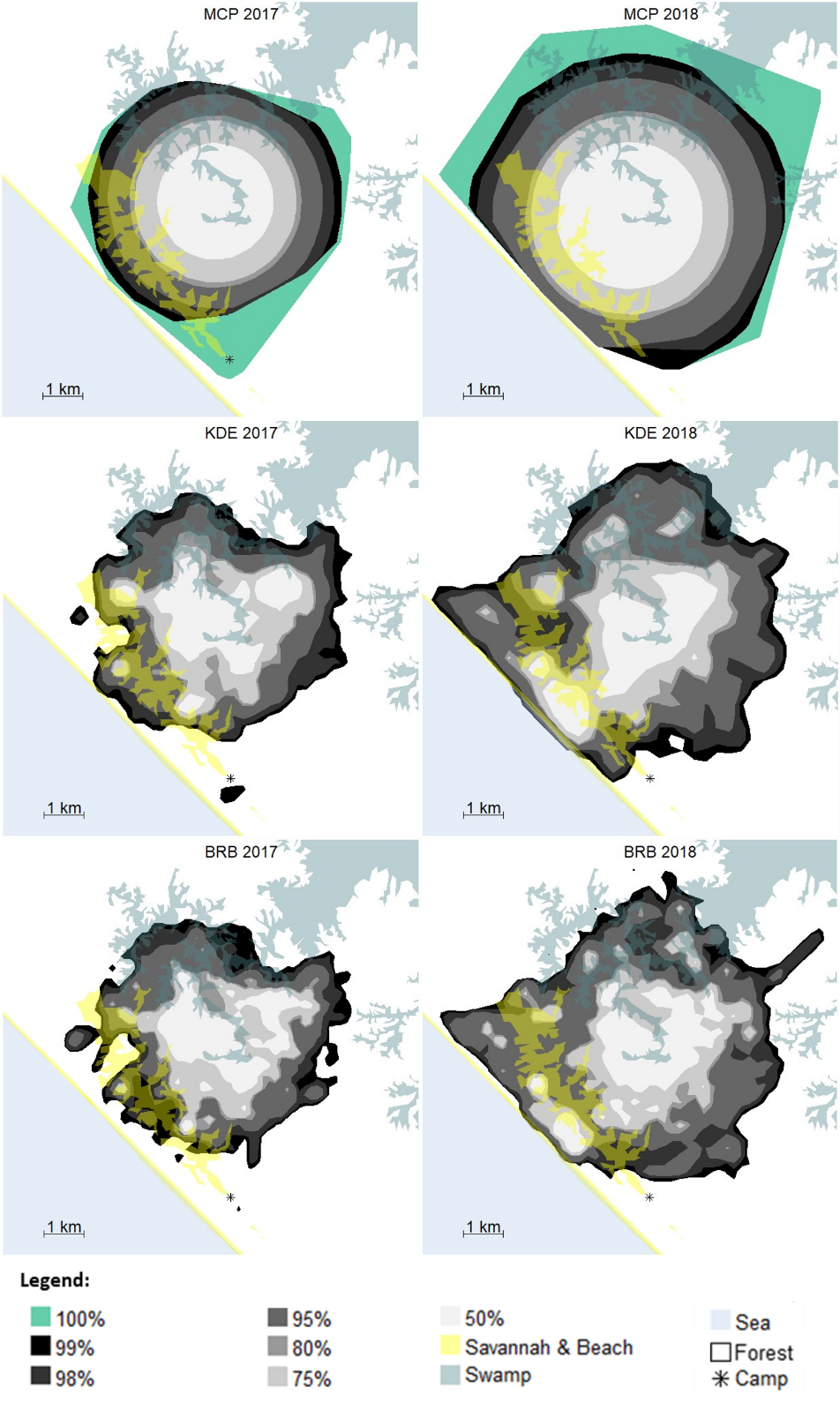


Fig. S2 Maps of annual Rekambo home range calculated with three methods: MCP, KDE_,_ and BRB. 2017: 205,545 relocations collected over 259 days and 2328 hours of observation. 2018: 340,263 relocations collected for 277 days and 2793 hours of observation. Maps created with R (v.4.0.2 R Core Team 2020).


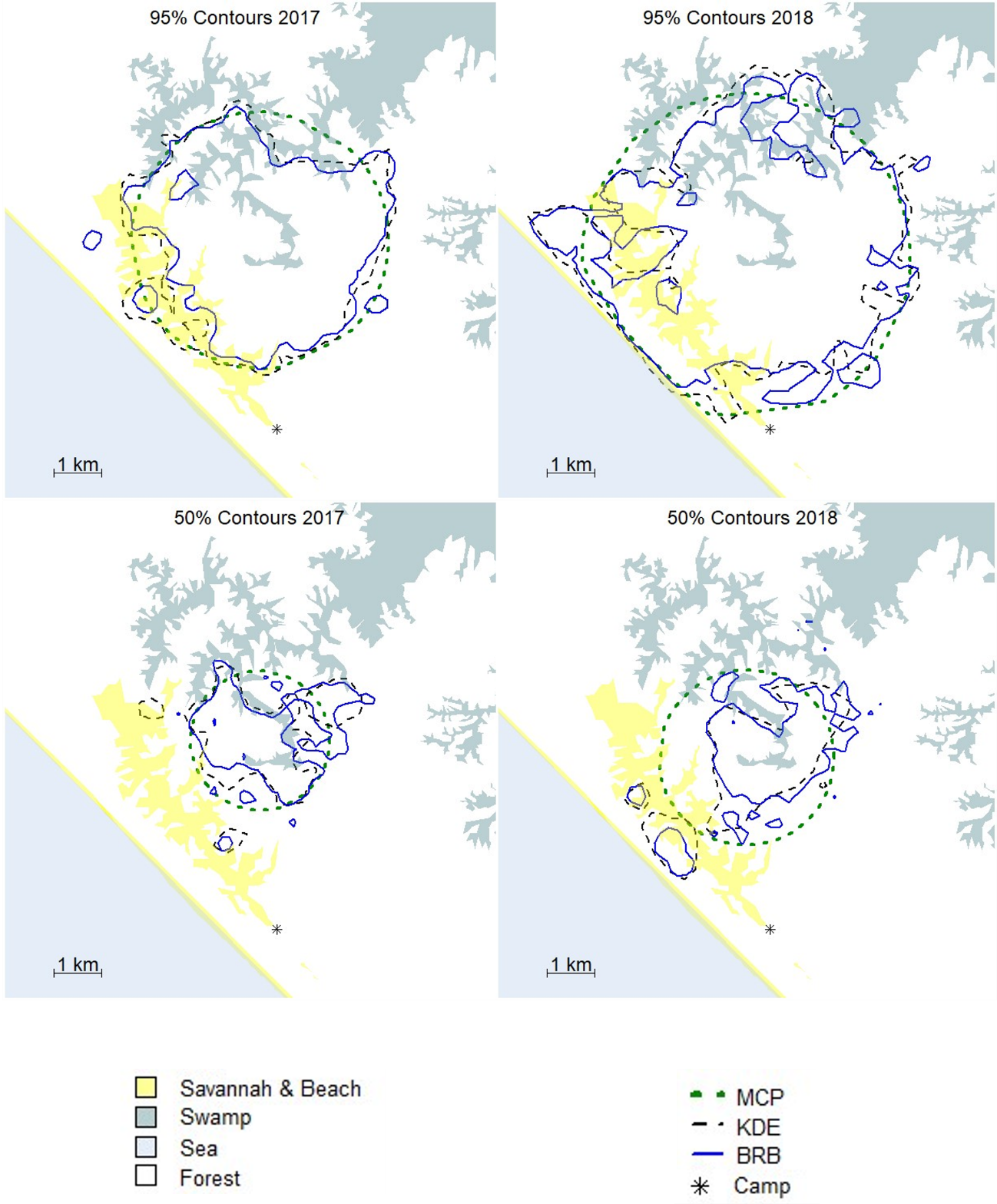


Fig. S3 Maps comparing the 95%, and 50% isopleths of the home range calculated for Rekambo community three different techniques (MCP, KDE, and BRB) in 2017 and 2018. Data: 2017: 221,981 relocations collected over 261 days and 2329.325 hours of observation. 2018: 369,754 relocations collected for 279 days and 2794.411 hours of observation. Maps created with R (v.4.0.2 R Core Team 2020).

| **Table S1 Home range size, community size and population density comparison across chimpanzee communities.** | | | | | | | | | | | | |
| --- | --- | --- | --- | --- | --- | --- | --- | --- | --- | --- | --- | --- |
| **Habitat** | **Community** | **Study period** | **Home range size (km^2^)** | | | | | | | | **Community**  **size** | **Population**  **density**  **(chimp/km^2^)** |
|  |  |  | **MCP %** | | | | | | | **Other**  **method** |  |  |
|  |  |  | **100** | **99** | **98** | **95** | **80** | **75** | **50** |  |  |  |
| Savannah-woodland | Mt.Assirik^V^ | 1976-1979^1^ | ≥37.4^OP^ |  |  |  |  |  |  | 51.4/72.1^IN^/278-333^IN^ | ≥23 | ≥0.61 |
| and gallery forest |  | (44 months) |  |  |  |  |  |  |  |  |  |  |
|  | Mugiri^S^ | 1996-1999^2^ | 50.1 |  |  |  |  |  |  | 38.3 | ≥29 | ≥0.58 |
|  |  | (600 days) |  |  |  |  |  |  |  |  |  |  |
|  |  | 1996-2011^3^ | 72.1 |  |  |  |  |  |  |  | ≈104 | ≈1.44 |
|  |  |  |  |  |  |  |  |  |  |  |  |  |
| Savannah-woodland | Fongoli^V^ | 2001-2004^4^ |  |  |  |  |  |  |  | ≥63^IN^ | ≥32 | ≥0.5 |
|  |  | (40 months) |  |  |  |  |  |  |  |  |  |  |
|  |  | 2001-2014^5*^ |  |  |  |  |  |  |  | 86 | 31.7 | 0.37 |
|  |  |  |  |  |  |  |  |  |  |  |  |  |
|  |  | 2005-2014^6^ | 110.39 |  |  |  |  |  | 9.97 | 38.72K | 31.6 | 0.29 |
|  |  | (120 months) |  |  |  |  |  |  |  |  |  |  |
|  |  | 2005-2014^6^ | 64.6 |  |  |  |  |  | 9.2 | 35.34K | 31.6 | 0.49 |
|  |  | (annual mean) |  |  |  |  |  |  |  |  |  |  |
|  | Kasakati L^S^ | 1963-1967^7^ |  |  |  |  |  |  |  | 124 | ≥10 | ≥0.08 |
|  |  | (23 months) |  |  |  |  |  |  |  |  |  |  |
|  | Kasakati Z^S^ | 1963-1967^7^ |  |  |  |  |  |  |  | 122 | ≥40 | 0.34 |
|  |  | (23 months) |  |  |  |  |  |  |  |  |  |  |
|  | Ugalla^S^ | 1995-2003^8^ |  |  |  |  |  |  |  | 400-500^IN^ | 30-35 | 0.07 |
|  |  | (5 months) |  |  |  |  |  |  |  |  |  |  |

| **Table S1 (Cont. I)** | | | | | | | | | | | | | |
| --- | --- | --- | --- | --- | --- | --- | --- | --- | --- | --- | --- | --- | --- |
| **Habitat** | **Community** | **Study period** | **Home range size (km^2^)** | | | | | | | | **Community**  **size** | **Population**  **density (chimp/km^2^)** |  |
|  |  |  | **MCP %** | | | | | | | **Other**  **method** |  |  |  |
|  |  |  | **100** | **99** | **98** | **95** | **80** | **75** | **50** |  |  |  |  |
| Submontane forest | Ngel Nyaki^E^ | 2005-2006^9^ |  |  |  |  |  |  |  | 7.5^IN^ | ≥12 | 1.60 |  |
|  |  | (4 months) |  |  |  |  |  |  |  |  |  |  |  |
| Riverine forest, grassland, | Bulindi^W^ | 2006-2008^10^ | ≥21^IN^ |  |  |  |  |  |  |  | - | - |  |
| swamp and plantations |  | (15 months) |  |  |  |  |  |  |  |  |  |  |  |
| Riverine forest, plantations, | Wagaisa^W^ | 2018^11^ | 42.5 |  |  | 28.2 |  |  |  | 52.9^OP^,16.7K | 34-37 | 0.80 |  |
| and human infrastructures |  | (3 months) |  |  |  |  |  |  |  |  |  |  |  |
| Montane forest | Kaboko^S^ | 1991-1999^12^ |  |  |  |  |  |  |  | 12.8 | 22.5 | 1.76 |  |
| and swamp |  | (729 days) |  |  |  |  |  |  |  |  |  |  |  |
|  |  | 1991-1999^12^ |  |  |  |  |  |  |  | 7.6 | 22.5 | 0.59 |  |
|  |  | (annual mean) |  |  |  |  |  |  |  |  |  |  |  |
| Montane forest | Cyamudongo^S^ | 2005-2015^13^ | 8.18 |  |  | 5.11 |  |  | 0.62 | 3.72K | 35-40 | 4.58 |  |
| and plantations |  | (132 months) |  |  |  |  |  |  |  |  |  |  |  |
| Montane forest | Mayebe^S^ | 2016-2017^14^ | 41.27 |  |  |  |  |  |  | 34,46K | 67 | 1.62 |  |
|  |  | (13 months) |  |  |  |  |  |  |  |  |  |  |  |
|  |  | 2000-2015^13^ | 60.98 |  |  | 25.31 |  |  | 5.63 | 21.23K | 50-60 | 0.90 |  |
|  |  | (192 months) |  |  |  |  |  |  |  |  |  |  |  |
| Forest-woodland | Kwano^E^ | 2000-2001^15^ |  |  |  |  |  |  |  | ≥26.2^IN^ | ≥36 | 1.30 |  |
|  |  | (358 days) |  |  |  |  |  |  |  |  |  |  |  |
| Forest, woodland | Mitumba^S^ | 1985-2014^5*^ |  |  |  |  |  |  |  | 4.6 | 24.6 | 5.35 |  |
| and grassland |  |  |  |  |  |  |  |  |  |  |  |  |  |

| **Table S1 (Cont. II)** | | | | | | | | | | | | |
| --- | --- | --- | --- | --- | --- | --- | --- | --- | --- | --- | --- | --- |
| **Habitat** | **Community** | **Study period** | **Home range size (km^2^)** | | | | | | | | **Community**  **size** | **Population**  **density**  **(chimp/km^2^)** |
|  |  |  | **MCP %** | | | | | | | **Other**  **method** |  |  |
|  |  |  | **100** | **99** | **98** | **95** | **80** | **75** | **50** |  |  |  |
| Forest, woodland | Kahama^S^ | 1973-1976^5*^ |  |  |  |  |  |  |  | 4.1 | 15.6 | 3.80 |
| and grassland |  |  |  |  |  |  |  |  |  |  |  |  |
|  | Kasekela^S^ | 1975–1978^16^ |  | 12 |  |  |  |  |  |  | 32-60 | 3.83 |
|  |  | (48 months) |  |  |  |  |  |  |  |  |  |  |
|  |  | 1979–1982^16^ |  | 5.4 |  |  |  |  |  |  | 32-60 | 8.52 |
|  |  | (48 months) |  |  |  |  |  |  |  |  |  |  |
| Forest, swamps | Kanyatale^S^ | 1997-2014^5*^ |  |  |  |  |  |  |  | 12 | 84 | 7.00 |
| and grassland |  |  |  |  |  |  |  |  |  |  |  |  |
|  | Kanyawara^S^ | 1988-1991^17^ | >14.9 |  |  |  |  |  |  |  | ≥41 | ≥2.75 |
|  |  | (37 months) |  |  |  |  |  |  |  |  |  |  |
|  |  | 1992-2006^18^ | 41.4 |  |  |  |  |  |  |  | 43-51 | 2.87 |
|  |  | (180 months) |  |  |  |  |  |  |  |  |  |  |
|  |  | 1996-1998^19^ | 37.8 |  |  |  |  |  |  |  | 50 | 1.32 |
|  |  | (30 months) |  |  |  |  |  |  |  |  |  |  |
|  |  | 1992-2006^18^ |  |  | 16.4 |  |  |  |  |  | 43-52 | 1.61 |
|  |  | (annual median) |  |  |  |  |  |  |  |  |  |  |
|  | Kyambura^S^ | 1994^5*^ |  |  |  |  |  |  |  | 4.3 | 18 | 4.19 |
|  |  |  |  |  |  |  |  |  |  |  |  |  |
|  | Sonso^S^ | 1997 - 1998^20^ | 9.68 |  |  |  | 3 |  |  | 9.19 | 56 | 8.26 |
|  |  | (19 months) |  |  |  |  |  |  |  |  |  |  |

| **Table S1 (Cont. III)** | | | | | | | | | | | | |
| --- | --- | --- | --- | --- | --- | --- | --- | --- | --- | --- | --- | --- |
| **Habitat** | **Community** | **Study period** | **Home range size (km^2^)** | | | | | | | | **Community**  **size** | **Population**  **density**  **(chimp/km^2^)** |
|  |  |  | **MCP %** | | | | | | | **Other**  **method** |  |  |
|  |  |  | **100** | **99** | **98** | **95** | **80** | **75** | **50** |  |  |  |
| Forest, swamps | Sonso^S^ (cont.) | 1994-1995^21^ | 6.78 |  |  |  |  |  |  | 6.89 | 38-46 | 6.19 |
| and grassland (cont.) |  | (16 months) |  |  |  |  |  |  |  |  |  |  |
| **Forest, savannah,** | **Rekambo^T^** | **2005-2008^22^** | **≥45^IN^** |  |  |  |  |  |  |  | **≥47** | **≥1.04** |
| **mangroves and swamps** |  | **(42 months)** |  |  |  |  |  |  |  |  |  |  |
|  |  | **2009-2011^23^** | **36^OP^** |  |  |  |  |  |  |  | - | - |
|  |  | **(36 months)** |  |  |  |  |  |  |  |  |  |  |
|  |  | **2009-2010^23^** | **≥24.4^IN^** |  |  |  |  |  |  |  | **≥45** | **≥1.84** |
|  |  | **(20 months)** |  |  |  |  |  |  |  |  |  |  |
|  |  | **2017-2018^24A^** | **45.37** | **36.57** | **33.49** | **28.29** | **17.75** | **15.86** | **8.46** | **26.21K** | **44-47** | **0.99** |
|  |  | **(annual mean)** |  |  |  |  |  |  |  |  |  |  |
|  |  | **2017-2019^24B^** | **59.03** | **49.67** | **45.22** | **38.12** | **21.31** | **18.74** | **9.63** | **30.39K** | **44-47** | **0.77** |
|  |  | **(28 months)** |  |  |  |  |  |  |  |  |  |  |
|  |  | **2017-2019^24C^** | **40.87** | **36.77** | **35.09** | **30.82** | **18.31** | **16.70** | **8.03** | **36.95K** | **44-47** | **1.11** |
|  |  | (28 months) |  |  |  |  |  |  |  |  |  |  |
| Forest, savannah, | Caiquene-Cadique^V^ | 2013-2014^25^ |  |  |  |  |  |  |  | 8 | ≥40 | 5.00 |
| mangroves and plantations |  | (12 months) |  |  |  |  |  |  |  |  |  |  |
| Forest | Moto^T^ | 2000-2002^26^ | 19.2 |  |  |  |  |  |  | 17.3K | >43 | >2.23 |
| and swamp |  | (24 months) |  |  |  |  |  |  |  |  |  |  |
|  |  | 1999-2014^5*^ |  |  |  |  |  |  |  | 25 | 42 | 1.68 |

| **Table S2 (Cont. IV)** | | | | | | | | | | | | |
| --- | --- | --- | --- | --- | --- | --- | --- | --- | --- | --- | --- | --- |
| **Habitat** | **Community** | **Study period** | **Home range size (km^2^)** | | | | | | | | **Community**  **size** | **Population density**  **(chimp/km^2^)** |
|  |  |  | **MCP %** | | | | | | | **Other**  **method** |  |  |
|  |  |  | **100** | **99** | **98** | **95** | **80** | **75** | **50** |  |  |  |
| Forest | Bossou^V^ | 1976-2014^5*^ |  |  |  |  |  |  |  | 9 | 17 | 1.89 |
| and plantations |  |  |  |  |  |  |  |  |  |  |  |  |
|  |  | - |  |  |  |  |  |  |  | 15 | 12 | 1.25 |
|  |  |  |  |  |  |  |  |  |  |  |  |  |
| Forest | Ngogo^S^ | 1999-2008^28^ | *28.76^ⱡ^* |  |  |  |  |  |  |  | 145 – 160 | 5.30 |
| and grassland |  | (38 months) |  |  |  |  |  |  |  |  |  |  |
|  |  | 2003-2006^29^ | *27.7^ⱡ^* |  |  |  |  | 9.3^ⱡ^ | 4.2^ⱡ^ | 19.5 | 140-150 | 5.14 |
|  |  | (19 months) |  |  |  |  |  |  |  |  |  |  |
|  |  | 200928 | *35.16^ⱡ^* |  |  |  |  |  |  |  | 145 – 160 | 4.35 |
|  |  | (5 months) |  |  |  |  |  |  |  |  |  |  |
| Forest | Madina^V^ | 2007^30^ | ≥19.02^IN^ |  |  |  | ≥6.82^IN^ | 5.62^IN^ | ≥2.72^IN^ | 8.52K^IN^ | - | - |
| and farm |  | (6 months) |  |  |  |  |  |  |  |  |  |  |
| Forest | Kalinzu M^S^ | 1997-1998^31^ |  |  |  |  |  |  |  | 12 | >24 | >2 |
|  |  | (10 months) |  |  |  |  |  |  |  |  |  |  |
|  |  | 1997-2014^5*^ |  |  |  |  |  |  |  | 10.5 | 73 | 6.95 |
|  |  |  |  |  |  |  |  |  |  |  |  |  |
|  | Mahale K^S^ | 1965-2014^5*^ |  |  |  |  |  |  |  | 10.4 | 23.5 | 2.26 |
|  |  |  |  |  |  |  |  |  |  |  |  |  |
|  | Mahale M^S^ | 1994-2009^32^ | 18.4 |  |  |  |  |  | 4.3 |  | 47-71 | 3.21 |
|  |  | (annual mean) |  |  |  |  |  |  |  |  |  |  |

| **Table S1 (Cont. V)** | | | | | | | | | | | | |
| --- | --- | --- | --- | --- | --- | --- | --- | --- | --- | --- | --- | --- |
| **Habitat** | **Community** | **Study period** | **Home range size (km^2^)** | | | | | | | | **Community**  **size** | **Population**  **density**  **(chimp/km^2^)** |
|  |  |  | **MCP %** | | | | | | | **Other**  **method** |  |  |
|  |  |  | **100** | **99** | **98** | **95** | **80** | **75** | **50** |  |  |  |
| Forest (cont.) | Mahale M^S^ (cont.) | 1994-2009^32^ | 27.5 |  |  |  |  |  |  |  | 47-70 | 2.13 |
|  |  | (192 months) |  |  |  |  |  |  |  |  |  |  |
|  | Seringbara^V^ | 2012-2013^33^ | ≥29.03^IN^ |  |  |  |  | 16.43^IN^ |  | 35.73^IN^ | ≥100 | ≥3.45 |
|  |  | (12 months) |  |  |  |  |  |  |  |  |  |  |
|  | Taï East^V^ | 2005-2007^34^ |  |  |  | 31 |  |  |  | 30.1K | 25 | 0.81 |
|  |  | (18 months) |  |  |  |  |  |  |  |  |  |  |
|  |  | 2013-2015^35^ |  |  |  |  |  |  |  | 40.37K | 36 | 0.89 |
|  |  | (297 days) |  |  |  |  |  |  |  |  |  |  |
|  | Taï Middle^V^ | 1996-1997^36^ | 12.1 |  |  | 9 |  | 2.3 | 0.6 |  | 11 | 0.91 |
|  |  | (10 months) |  |  |  |  |  |  |  |  |  |  |
|  |  | 2003-2004^34^ |  |  |  | 8.1 |  |  |  | 9.7K | 4 | 0.41 |
|  |  | (9 months) |  |  |  |  |  |  |  |  |  |  |
|  | Taï North^V^ | 1996-1997^36^ | 16.8 |  |  | 10.5 |  | 4.9 | 2 |  | 35 | 2.08 |
|  |  | (10 months) |  |  |  |  |  |  |  |  |  |  |
|  |  | 2004-2005^34^ |  |  |  | 13.3 |  |  |  | 15K | 18 | 1.20 |
|  |  | (12 months) |  |  |  |  |  |  |  |  |  |  |
|  | Taï South^V^ | 2004-2006^36^ |  |  |  | 27.2 |  |  |  | 26.5K | 33 | 1.21 |
|  |  | (20 months) |  |  |  |  |  |  |  |  |  |  |
|  |  | 1996-1997^34^ | 26.5 |  |  | 13.5 |  | 3.5 | 1.5 |  | 63 | 2.38 |
|  |  | (10 months) |  |  |  |  |  |  |  |  |  |  |
| Community^Chimpanzee subspecies^: ^S^=Eastern chimpanzee (Pan troglodytes schweinfurthii); ^V^=Western chimpanzee (P.t. verus); ^T^ =Central chimpanzee (P.t. troglodytes); ^E^=Nigerian-Cameroon chimpanzee (P.t.ellioti). Study period^Reference^**^:^ 1**. (Baldwin et al. 1982), **2.** (Hunt and McGrew 2002), **3.**(Samson and Hunt 2012), **4**.(Pruetz 2006), **5*** (Wilson et al. 2014) who report mean size of community's home range.**6.** (Boyer Ontl 2017), **7.** (Izawa 1970), **8.** (Ogawa et al. 2007), **9.** (Beck and Chapman 2008), **10.** (Mclennan 2010), **11.** (McLennan et al. 2020)**, 12**.(Basabose 2005), **13.** (Moore et al. 2018), **14.** (Green et al. 2020), **15.** (Sommer et al. 2004), **16.** (Williams et al. 2002), **17.** (Chapman and Wrangham 1993), **18.** (Wilson et al. 2012), **19.** (Wilson et al. 2001)**, 20.** (Fawcett 2000)**, 21.** (Newton-Fisher 2003)**, 22.**  (Arandjelovic et al. 2011), **23.** (Head et al. 2013), **24A.**Present study: Averaged annual calculations (2017: 221,981 relocations ; 2018: 369,754 relocations). **24B.**Present study: Calculations made with all available data (N=724,286 relocations); **24C**.Present study: Calculated by randomly selecting 1 relocation per day (N=643) and averaging the result of 10 repetitions. **25.** (Bessa et al. 2015)**, 26.** (Morgan et al. 2006), **27.** (Hockings et al. 2006)**, 28.** (Mitani et al. 2010), **29.** (Amsler 2009), **30.**(Vieira et al. 2019), **31.**(Hashimoto et al. 2001), **32.** (Nakamura et al. 2013), **33.** (Montanari 2014), **34.** (Kouakou et al. 2011), **35.**(Després-Einspenner et al. 2017), **36.** (Herbinger et al. 2001). ^ⱡ^Patrols and incursions excluded from calculations. K= "Other method" was KDE 95% (does not account for differences in bandwidth nor type of kernel). ^IN^= calculation was based on indirect data, such as nest counts, density extrapolations, etc. ^OP^= From opportunistic observations as opposed to systematic follows. Community size includes all age-sex classes. Population density calculated by dividing (average) community size by the maximum MCP available or the greatest alternative if MCP was not calculated. | | | | | | | | | | | | |

**References from Table S1**

Amsler S (2009) Ranging behavior and territoriality in chimpanzees at Ngogo, Kibale National Park, Uganda. Dissertation, University of Michigan

Arandjelovic M, Head J, Rabanal LI, et al (2011) Non-Invasive Genetic Monitoring of Wild Central Chimpanzees. PLoS One 6:e14761. https://doi.org/10.1371/journal.pone.0014761

Baldwin PJ, McGrew WC, Tutin CEG (1982) Wide-ranging chimpanzees at Mt. Assirik, Senegal. Int J Primatol 3:367–385. https://doi.org/10.1007/BF02693739

Basabose AK (2005) Ranging patterns of chimpanzees in a montane forest of Kahuzi, Democratic Republic of Congo. Int J Primatol 26:33–54. https://doi.org/10.1007/s10764-005-0722-1

Beck J, Chapman H (2008) A population estimate of the endangered chimpanzee *Pan troglodytes vellerosus* in a Nigerian montane forest: Implications for conservation. Oryx 42:448–451. https://doi.org/10.1017/S0030605308001397

Bessa J, Sousa C, Hockings KJ (2015) Feeding ecology of chimpanzees (*Pan troglodytes verus*) inhabiting a forest-mangrove-savanna-agricultural matrix at Caiquene-Cadique, Cantanhez National Park, Guinea-Bissau. Am J Primatol 77:651–665. https://doi.org/10.1002/ajp.22388

Boyer Ontl KM (2017) Chimpanzees in the Island of Gold: Impacts of artisanal small-scale gold mining on chimpanzees (*Pan troglodytes verus*) in Fongoli, Senegal. Iowa State University

Chapman CA, Wrangham RW (1993) Range use of the forest chimpanzees of Kibale: Implications for the understanding of chimpanzee social organization. Am J Primatol 31:263–273. https://doi.org/10.1002/ajp.1350310403

Després-Einspenner ML, Howe EJ, Drapeau P, Kühl HS (2017) An empirical evaluation of camera trapping and spatially explicit capture-recapture models for estimating chimpanzee density. Am J Primatol 79:1–12. https://doi.org/10.1002/ajp.22647

Fawcett KA (2000) Female relationships and food availability in a forest community of chimpanzees . Dissertation, University of Edinburgh

Green SJ, Boruff BJ, Grueter CC (2020) From ridge tops to ravines: landscape drivers of chimpanzee ranging patterns. Anim Behav 163:51–60. https://doi.org/10.1016/j.anbehav.2020.02.016

Hashimoto C, Furuichi T, Tashiro Y (2001) What factors affect the size of chimpanzee parties in the Kalinzu forest, Uganda? Int J Primatol 22:947–959

Head JS, Boesch C, Robbins MM, et al (2013) Effective sociodemographic population assessment of elusive species in ecology and conservation management. Ecol Evol 3:2903–2916. https://doi.org/10.1002/ece3.670

Herbinger I, Boesch C, Rothe H (2001) Territory characteristics among three neighboring chimpanzee communities in the Taï National Park, Côte d’Ivoire. Int J Primatol 22:143–167. https://doi.org/10.1023/A:1005663212997

Hockings KJ, Anderson JR, Matsuzawa T (2006) Road crossing in chimpanzees: A risky business. Curr Biol 16:668–670. https://doi.org/10.1016/j.cub.2006.08.019

Hunt KD, McGrew WC (2002) Chimpanzees in the dry habitats of Assirik, Senegal and Semliki Wildlife Reserve, Uganda. In: Boesch C, Hohmann G, Marchant L (eds) Behavioural Diversity in Chimpanzees and Bonobos. Cambridge University Press, Cambridge, pp 35–51

Izawa K (1970) Unit groups of chimpanzees and their nomadism in the savanna woodland. Primates 11:1–45. https://doi.org/10.1007/BF01730674

Kouakou CY, Boesch C, Kuehl HS (2011) Identifying hotspots of chimpanzee group activity from transect surveys in Taï National Park, Côte d’Ivoire. J Trop Ecol 27:621–630. https://doi.org/10.1017/S0266467411000423

Mclennan MR (2010) Chimpanzee ecology and interactions with people in an unprotected human-dominated landscape at Bulindi , Western Uganda. Oxford Brookes University

McLennan MR, Hintz B, Kiiza V, et al (2020) Surviving at the extreme: Chimpanzee ranging is not restricted in a deforested human‐dominated landscape in Uganda. Afr J Ecol 1–12. https://doi.org/10.1111/aje.12803

Mitani JC, Watts DP, Amsler SJ (2010) Lethal intergroup aggression leads to territorial expansion in wild chimpanzees. Curr Biol 20:507–508. https://doi.org/10.1016/j.cub.2010.04.021

Montanari D (2014) Ranging patterns of Seringbara chimpanzees: methodological insights. Dissertation, Universiteit Utrecht

Moore JF, Mulindahabi F, Gatorano G, et al (2018) Shifting through the forest: home range, movement patterns, and diet of the eastern chimpanzee ( *Pan troglodytes schweinfurthii* ) in Nyungwe National Park, Rwanda. Am J Primatol 80:e22897. https://doi.org/10.1002/ajp.22897

Morgan D, Sanz C, Onononga JR, Strindberg S (2006) Ape abundance and habitat use in the Goualougo Triangle, Republic of Congo. Int J Primatol 27:147–179. https://doi.org/10.1007/s10764-005-9013-0

Nakamura M, Corp N, Fujimoto M, et al (2013) Ranging behavior of Mahale chimpanzees: A 16 year study. Primates 54:171–182. https://doi.org/10.1007/s10329-012-0337-z

Newton-Fisher NE (2003) The home range of the Sonso community of chimpanzees from the Budongo Forest, Uganda. Afr J Ecol 41:150–156. https://doi.org/10.1046/j.1365-2028.2003.00408.x

Ogawa H, Idani G, Moore J, et al (2007) Sleeping parties and nest distribution of chimpanzees in the savanna woodland, Ugalla, Tanzania. Int J Primatol 28:1397–1412. https://doi.org/10.1007/s10764-007-9210-0

Pruetz JD (2006) Feeding ecology of savanna chimpanzees (*Pan troglodytes verus*) at Fongoli, Senegal. In: Hohmann G, Robbins MM, Boesch C (eds) Feeding ecology in apes and other primates. Ecological, physocal and behavioral aspects. Cambridge University Press, Cambridge, pp 161–182

Samson DR, Hunt KD (2012) A Thermodynamic Comparison of Arboreal and Terrestrial Sleeping Sites for Dry-Habitat Chimpanzees (*Pan troglodytes schweinfurthii*) at the Toro-Semliki Wildlife Reserve, Uganda. Am J Primatol 74:811–818. https://doi.org/10.1002/ajp.22031

Sommer V, Fowler A, Adanu J, et al (2004) Nigerian chimpanzees ( *Pan troglodytes vellerosus* ) at Gashaka : Two years of habituation efforts. Folia Primatol 75:295–316. https://doi.org/10.1159/000080208

Vieira WF, Kerry C, Hockings KJ (2019) A comparison of methods to determine chimpanzee home-range size in a forest–farm mosaic at Madina in Cantanhez National Park, Guinea-Bissau. Primates 60:355–365. https://doi.org/10.1007/s10329-019-00724-1

Williams JM, Pusey AE, Carlis J V., et al (2002) Female competition and male territorial behaviour influence female chimpanzees’ ranging patterns. Anim Behav 63:347–360. https://doi.org/10.1006/anbe.2001.1916

Wilson ML, Boesch C, Fruth B, et al (2014) Lethal aggression in Pan is better explained by adaptive strategies than human impacts. Nature 513:414–417. https://doi.org/10.1038/nature13727

Wilson ML, Hauser MD, Wrangham RW (2001) Does participation in intergroup conflict depend on numerical assessment, range location, or rank for wild chimpanzees? Anim Behav 61:1203–1216. https://doi.org/10.1006/anbe.2000.1706

Wilson ML, Kahlenberg SM, Wells M, Wrangham RW (2012) Ecological and social factors affect the occurrence and outcomes of intergroup encounters in chimpanzees. Anim Behav 83:277–291. https://doi.org/10.1016/j.anbehav.2011.11.004

| Table S2 Monthly size and composition of the Rekambo community | | | | | | | | | | | |
| --- | --- | --- | --- | --- | --- | --- | --- | --- | --- | --- | --- |
|  | **AF** | **AM** | **AU** | **ADF** | **ADM** | **JF** | **JM** | **IF** | **IM** | **IU** | **Community**  **size** |
| Jan-17 | 17 | 9 | 1 | 8 | 4 | 2 | 3 | 1 | 1 | 1 | **47** |
| Feb-17 | 17 | 9 | 1 | 8 | 3 | 2 | 3 | 1 | 1 | 1 | **46** |
| Mar-17 | 17 | 9 | 1 | 8 | 3 | 2 | 3 | 1 | 1 | 1 | **46** |
| Apr-17 | 17 | 9 | 1 | 8 | 3 | 2 | 3 | 1 | 1 | 1 | **46** |
| May-17 | 17 | 9 | 1 | 8 | 3 | 2 | 3 | 1 | 1 | 1 | **46** |
| Jun-17 | 17 | 9 | 1 | 8 | 3 | 2 | 3 | 1 | 1 | 1 | **46** |
| Jul-17 | 17 | 9 | 1 | 8 | 3 | 2 | 3 | 1 | 1 | 1 | **46** |
| Aug-17 | 17 | 9 | 1 | 8 | 3 | 2 | 3 | 1 | 1 | 1 | **46** |
| Sep-17 | 17 | 9 | 1 | 7 | 3 | 2 | 3 | 1 | 1 | 1 | **45** |
| Oct-17 | 17 | 9 | 1 | 7 | 3 | 2 | 3 | 1 | 1 | 1 | **45** |
| Nov-17 | 17 | 9 | 1 | 7 | 3 | 2 | 3 | 1 | 1 | 1 | **45** |
| Dec-17 | 17 | 9 | 1 | 7 | 3 | 2 | 3 | 1 | 1 | 1 | **45** |
| Jan-18 | 17 | 9 | 1 | 7 | 3 | 2 | 3 | 1 | 2 | 1 | **46** |
| Feb-18 | 17 | 9 | 0 | 7 | 3 | 2 | 3 | 1 | 2 | 1 | **45** |
| Mar-18 | 17 | 9 | 0 | 7 | 3 | 2 | 3 | 1 | 2 | 1 | **45** |
| Apr-18 | 17 | 9 | 0 | 7 | 3 | 2 | 3 | 1 | 2 | 1 | **45** |
| May-18 | 17 | 9 | 0 | 7 | 3 | 2 | 3 | 1 | 2 | 1 | **45** |
| Jun-18 | 17 | 9 | 0 | 8 | 3 | 2 | 3 | 3 | 2 | 0 | **47** |
| Jul-18 | 16 | 9 | 0 | 8 | 3 | 1 | 3 | 3 | 2 | 1 | **46** |
| Aug-18 | 16 | 9 | 0 | 8 | 3 | 1 | 3 | 3 | 2 | 0 | **45** |
| Sep-18 | 16 | 9 | 0 | 8 | 3 | 1 | 3 | 3 | 2 | 0 | **45** |
| Oct-18 | 16 | 8 | 0 | 8 | 3 | 1 | 3 | 3 | 2 | 0 | **44** |
| Nov-18 | 16 | 8 | 0 | 8 | 3 | 1 | 3 | 3 | 2 | 0 | **44** |
| Dec-18 | 16 | 8 | 0 | 8 | 3 | 1 | 3 | 3 | 2 | 0 | **44** |
| Jan-19 | 16 | 8 | 0 | 8 | 3 | 1 | 3 | 4 | 2 | 0 | **45** |
| Feb-19 | 16 | 8 | 0 | 7 | 3 | 1 | 3 | 4 | 2 | 0 | **44** |
| Mar-19 | 16 | 8 | 0 | 7 | 3 | 1 | 3 | 4 | 2 | 0 | **44** |
| Apr-19 | 16 | 8 | 0 | 7 | 3 | 1 | 3 | 4 | 3 | 0 | **45** |
| May-19 | 16 | 8 | 0 | 7 | 3 | 1 | 3 | 5 | 3 | 0 | **46** |
| Jun-19 | 16 | 8 | 0 | 6 | 3 | 1 | 3 | 5 | 3 | 0 | **45** |
| AF= Adult female; AM= Adult Male; AU = Adult of unknown sex; ADF= Adolescent Female; ADM = Adolescent Male; JF = Juvenile Female; JM= Juvenile Male; IF = Infant Female; IM=Infant Male; IU= Infant of Unknown sex. Age classes were assigned according to Goodall, J. (1968). The Behaviour of Free-living Chimpanzees in the Gombe Stream Reserve. Animal Behaviour Monographs, 1, 161-IN12. <https://doi.org/10.1016/s0066-1856(68)80003-2> | | | | | | | | | | | |

**R code to calculate and map the home ranges.**

Note: This code was designed to be run directly in R. Maps may be slightly different if the code is run in Rstudio (e.g., legends and scale may be placed incorrectly)

**Table of Contents**

[#### 1. Preparing R and the data for analysis ################# 18](#_Toc70373729)

[# 1.1 Installing and activating all the needed packages 18](#_Toc70373730)

[# 1.2 Uploading the landscape shapes for the map 18](#_Toc70373731)

[# 1.3. Uploading the tracks 19](#_Toc70373732)

[# 1.4 Eliminating duplicates (sometimes it happened with some time stamps) and NA (problems with lag calculation) 19](#_Toc70373733)

[# 1.5 Preparing the coordinates for the calculations (from WGS to UTM and transformed into spatial points) 19](#_Toc70373734)

[# 1.6 Counting amount of data used in the calculations 19](#_Toc70373735)

[# 1.7 Counting the number of relocations per day 19](#_Toc70373736)

[# 1.8 Splitting by year to calculate annual home ranges 19](#_Toc70373737)

[#### 2. Calculating the home range as a Minimum Convex Polygon (MCP) ###### 19](#_Toc70373738)

[# 2.1 Calculating the MCP 19](#_Toc70373739)

[# 2.2 Calculating the area of each MCP isopleth (km2) 20](#_Toc70373740)

[# 2.3 Plotting the MCPs on the map of the study site 20](#_Toc70373741)

[# 2.4 Saving the map 21](#_Toc70373742)

[#### 3. Calculating the home range with Kernel Density Estimation (KDE) ###### 21](#_Toc70373743)

[# 3.1 KDE (href) 21](#_Toc70373744)

[# 3.2 KDE (hLCSV) 22](#_Toc70373745)

[#### 4. Calculating the home range with Biased Random Bridges (BRB) ###### 24](#_Toc70373746)

[# 4.1 Preparing the data to create a ltraj object (a trayectory instead of the Spatial Points used in KDE and MCP) 24](#_Toc70373747)

[# 4.2 Estimating D (diffusion coefficient) 24](#_Toc70373748)

[# 4.3 Calculating the BRB 24](#_Toc70373749)

[# 4.4 Preparing the different isopleths to calculate their area 24](#_Toc70373750)

[# 4.4 Plotting the BRB home range over the map of the study site and calculation of the areas 25](#_Toc70373751)

[#### 5. Comparing contours across the three different estimators ###### 26](#_Toc70373752)

[# 5.1 Comparing 99% contours 26](#_Toc70373753)

[# 5.2 Comparing 75% contours 27](#_Toc70373754)

[# 5.3 Comparing 50% contours 28](#_Toc70373755)

[#### 6. Calculating the Area Under the Curve ###### 30](#_Toc70373756)

[# 6.1 AUC KDE (href) 30](#_Toc70373757)

[# 6.2 AUC BRB 30](#_Toc70373758)

[# 6.3 AUC MCP 31](#_Toc70373759)

[#### 7. Comparing the BRB home range calculated with tmax=120 minutes VS tmax 30 minutes ###### 31](#_Toc70373760)

[# 7.1 Recalculating the home range by BRB using 30 minutes as Tmax 31](#_Toc70373761)

[# 7.2 Plotting and calculating the BRB when Tmax=30 31](#_Toc70373762)

[# 7.3 Showing the area of the resulting isopleths (km2) 32](#_Toc70373763)

[# 7.4 Comparing 95% and 50% contours of BRB Tmax=30 and BRB Tmax=120 32](#_Toc70373764)

[# 7.5 Saving the map 33](#_Toc70373765)

[#### 8. Calculating and mapping the home range of the Rekambo community in 2017 33](#_Toc70373766)

[# 8.1 Preparing the data for analyses 33](#_Toc70373767)

[# 8.2 MCP 2017 34](#_Toc70373768)

[# 8.3 KDE (href) 2017 35](#_Toc70373769)

[# 8.4 BRB 2017 36](#_Toc70373770)

[# 8.5 Comparing the 95% and 50% contours of 2017 for the three estimators 37](#_Toc70373771)

[# 8.6 Areas Under the Curve 2017 40](#_Toc70373772)

[#### 9. Calculating and mapping the home range of the Rekambo community in 2018 42](#_Toc70373773)

[# 9.1 Preparing the data for analyses 42](#_Toc70373774)

[# 9.2 MCP of the Rekambo community in 2018 42](#_Toc70373775)

[# 9.3 KDE 2018 43](#_Toc70373776)

[# 9.4 BRB 2018 45](#_Toc70373777)

[# 9.5 Comparing the 95% and 50% contours of 2018 for the three estimators 46](#_Toc70373778)

[# 9.6 Area Under the Curve 2018 49](#_Toc70373779)

[#### 10. Effects of relocation subsampling in MCP and KDE ########## 50](#_Toc70373780)

[# 10.1 Preparing the data for the sub-sampling 50](#_Toc70373781)

[# 10.2 Calculating MCP and KDE with one sample per day (x10 trials) 50](#_Toc70373782)

[# 10.3 Calculating MCP and KDE with three samples per day (x10 trials) 63](#_Toc70373783)

[# 10.4 Calculating MCP and KDE with twelve samples per day (x10 trials) 76](#_Toc70373784)

[# 10.5 Comparing KDE across the three different subsampling schemes 89](#_Toc70373785)

[# 10.6 Comparing MPC across the three different subsampling schemes 91](#_Toc70373786)

#####################################################################

#### 1. Preparing R and the data for analysis #################

######################################################################

1.1 Installing and activating all the needed packagesif(!require(installr)) {

install.packages("installr"); require(installr)} #load / install+load installr #Helps to automatically detect if a new version of R needs to be installed# using the package:

updateR() # this will start the updating process of your R installation. It will check for newer versions, and if one is available, will guide you through the decisions you'd need to make.install.packages("devtools")#package needed to automatically check if it is necessary to install Rtools

library(devtools)

#REMEMBER TO INSTALL THE PROPER VERSION OF RTOOLS FROM: https://cran.r-project.org/bin/windows/Rtools/

install.packages("shapefiles")#Needed to read the shp of the swamps, savannah, etc

install.packages("rgdal")#Needed to manipulate shp and convert coordinates to utm and writeOGR

install.packages("adehabitatHR")#Needed to calculate MCP, KDE, and BRB

install.packages("splancs")# Needed to use the object areapl to calculate the different MCP contours

install.packages("spatstat")#Needed to manipulate shp and convert coordinates to utm

install.packages("adehabitatLT")#Needed to transform the tracklog data into a ltraj object so they can be used to calculate BRB in adehabitatHR

install.packages("caTools")#Needed to calculate AUC

install.packages("bitops")#Needed to calculate AUC

install.packages("raster")#Needed to calculate the AUC

install.packages("dplyr")#to count the number of relocations per day

library(shapefiles)

library(rgdal)

library(adehabitatHR)

library(splancs)

library(spatstat)

library(adehabitatLT)

library(bitops)

library(caTools)

library(bitops)

library(raster)

library(dplyr)

# 1.2 Uploading the landscape shapes for the map

source("C:/Users/Laura/Dropbox/Research/LoangoChimpanzeeProject/201909_intercommunity_study/R_map/split_shape.r")

source("C:/Users/Laura/Dropbox/Research/LoangoChimpanzeeProject/201909_intercommunity_study/R_map/geo_functions_bcgps.r")

savannah=read.shp("C:/Users/Laura/Dropbox/Research/LoangoChimpanzeeProject/201909_intercommunity_study/R_map/Savannah_polygone.shp")$shp

savannah=split.shape(shp=savannah)

swamp=read.shp("C:/Users/Laura/Dropbox/Research/LoangoChimpanzeeProject/201909_intercommunity_study/R_map/swamps1.shp")$shp

swamp=split.shape(shp=swamp)

sea=read.shp("C:/Users/Laura/Dropbox/Research/LoangoChimpanzeeProject/201909_intercommunity_study/R_map/Sea.shp")$shp

sea=split.shape(shp=sea)# 1.3. Uploading the tracks alltracks=read.table(file="C:/Users/Laura/Dropbox/Research/LoangoChimpanzeeProject/201909_intercommunity_study/2019refresh/202004_maps/202004_tracklogs_201701_to_201904.txt",sep="\t", header=TRUE)# 1.4 Eliminating duplicates (sometimes it happened with some time stamps) and NA (problems with lag calculation)

dup.ts=as.data.frame(duplicated(alltracks$date))#Like this we can see which rows have a duplicated time stamp in case we want to dig deeper

summary(dup.ts)#75623 duplicated time stamps

anyDuplicated(alltracks$date)

alltracks=alltracks[!duplicated(alltracks$date), ]#Eliminate rows with duplicated time stamp

anyDuplicated(alltracks$date)

any(is.na(alltracks))

alltracks<-na.omit(alltracks)

apply(alltracks, 2, function(x) any(is.infinite(x)))#No infinite values detected. Good!# 1.5 Preparing the coordinates for the calculations (from WGS to UTM and transformed into spatial points)

utm.coords=WGS.to.UTM(long=alltracks$lon, lat=alltracks$lat, utm.zone=utm.zone(mean(alltracks$lon), mean(alltracks$lat)), avoid.negatives=T, return.zone=F)

tracks=utm.coords

xyALL=SpatialPoints(coords=tracks[, c("long", "lat")])# 1.6 Counting amount of data used in the calculations

alltracks$yyyymmdd<-as.factor(alltracks$yyyymmdd)

str(alltracks)

#Calculating observation time included in the sample

#We will calculate the observation time with the calculation on lag. We will need to remove any with a

#lag >30min, which would be joining different tracks, so time in which there was no chimpanzee observation

lags<- alltracks[alltracks$lag <= 30,]

lags<- lags$lag/60

str(lags)

sum(lags)

# 1.7 Counting the number of relocations per day

df<-data.frame(alltracks$yyyymmdd)

pointsperday<-count(df,alltracks$yyyymmdd)

summary(pointsperday)# 1.8 Splitting by year to calculate annual home ranges

tracks.2017<- alltracks[alltracks$year=="2017",]

tracks.2018 <- alltracks[alltracks$year =="2018",]

###############################################################################

#### 2. Calculating the home range as a Minimum Convex Polygon (MCP) ######

###############################################################################

setwd("C:/Users/laura/Dropbox/Research/LoangoChimpanzeeProject/201909_intercommunity_study/2019refresh/202004_maps/") # 2.1 Calculating the MCPto.get=c(100, 99, 98, 95, 80, 75, 50)

MCP=vector("list", length(to.get))

names(MCP)=to.get

for(i in 1:length(to.get)){

xxALL=mcp(xyALL, percent=to.get[i])

MCP[[i]]=xxALL@polygons[[1]]@Polygons[[1]]@coords

}# 2.2 Calculating the area of each MCP isopleth (km2)

MCP.areas=unlist(lapply(MCP, areapl))

MCP.areas=MCP.areas/(1000^2)

names(MCP.areas)=to.get

MCP.areas# 2.3 Plotting the MCPs on the map of the study sitepar(mar=rep(0.2, 4))

plot(as.matrix(MCP[["100"]]), asp=1, xlab="", ylab="",xlim=c(548350, 557873),ylim=c(9765030, 9774195), axes=T, type="n", xaxt="n", yaxt="n", bty="n")

cols=c("mediumaquamarine", "gray0", "gray20", "gray40","gray60", "gray80", "gray95")

for(i in 1:length(to.get)){

polygon(MCP[[i]], border=NA, col=cols[i])

}

for(i in 1:length(savannah)){

polygon(savannah[[i]], border=NA, col=adjustcolor(col="yellow", alpha.f=0.4))

}

polygon(swamp[[10]], border=NA, col=adjustcolor(col="cadetblue4", alpha.f=0.4))

polygon(sea[[1]], border=NA, col=adjustcolor(col="lightsteelblue2", alpha.f=0.4))## 2.3.1 Adding the location of the Ozouga Research Camp to the map

camp.loc=c(x=553601, y=9765888)

points(x=camp.loc["x"], y=camp.loc["y"], pch=8, cex=1.2, lwd=1)## 2.3.2 Adding the scale

arrows(x0=par()[["usr"]][1]+1000, x1=par()[["usr"]][1]+2000, y0=par()[["usr"]][3]+500, y1=par()[["usr"]][3]+500, code=3, angle=90, length=0.05)

text(x=par()[["usr"]][1]+1500, y=par()[["usr"]][3]+700, labels="1 km",cex=1.5) ## 2.3.3 adding the legend

#(note that for the manuscript we ran the code of the legend independently

# from the rest of the map and then set all the figures together with the

#legend in PowerPoint)box.el=200

cols=c("mediumaquamarine", "gray0", "gray20", "gray40","gray60", "gray80", "gray95",

adjustcolor(col="yellow", alpha.f=0.4), adjustcolor(col="cadetblue4", alpha.f=0.4), adjustcolor(col="lightsteelblue2", alpha.f=0.4), "white", "black")

borders=rep("black", length(cols))

texts=c(paste(c(100, 99, 98, 95,80, 75, 50), "%", sep=""), "Savannah & Beach", "Swamp", "Sea", "Forest", "Camp")for(i in 1:length(texts)){

if(i<=11){

rect(xleft=par()[["usr"]][1]+0.5*box.el, xright=par()[["usr"]][1]+1.5*box.el,

ytop= par()[["usr"]][4]-box.el*0.5*i-box.el*(i-1),

ybottom=par()[["usr"]][4]-box.el*0.5*i-box.el*(i-1)-box.el,

col=cols[i], border="black")

}else{

points(x=par()[["usr"]][1]+box.el, y=par()[["usr"]][4]-box.el*0.5*i-box.el*(i-1)-box.el/2, pch=8)

}

text(x=par()[["usr"]][1]+1.5*box.el+50, y=par()[["usr"]][4]-box.el*0.5*i-box.el*(i-1)-box.el/2, labels=texts[i], adj=0, cex=0.8)

}

## 2.3.4 Adding the title to the map

text(x=par()[["usr"]][1]+5000, y=par()[["usr"]][3]+10000, labels="MCP", adj=0, cex=1.5)# 2.4 Saving the map

savePlot(file="C:/Users/laura/Dropbox/Research/LoangoChimpanzeeProject/201909_intercommunity_study/2019refresh/202004_maps/202009_MCP_home_range_Rekambo_201701_201904.png", type="png", device=dev.cur())

dev.copy2pdf(file="C:/Users/laura/Dropbox/Research/LoangoChimpanzeeProject/201909_intercommunity_study/2019refresh/202004_maps/202009_MCP_home_range_Rekambo_201701_201904.pdf")

###############################################################################

#### 3. Calculating the home range with Kernel Density Estimation (KDE) ######

###############################################################################

####### 3.1 KDE (href) ######

## 3.1.1 Calculating KDE (href)

k.data=SpatialPointsDataFrame(coords=tracks, data=data.frame(id=rep("a", nrow(tracks))))

xkernel=kernelUD(k.data, h="href", grid=100)xkernel[[1]]@h#this shows the value of h## 3.1.2 Setting the isopleths to calculate the areas of each

kernel.d=rev(c(50, 75, 80, 95,98,99))

kernel.areas=rep(0, length(kernel.d))

names(kernel.areas)=kernel.d

## 3.1.3 Plotting the KDE (href) on the map of the study site and calculating the areaspar(mar=rep(0.2, 4))

plot(getverticeshr(xkernel, percent=99), asp=1, xlab="", ylab="",xlim=c(548350, 557873),ylim=c(9765030, 9774195), axes=F, xaxt="n", yaxt="n", bty="n", border=NA)

cols=c("gray0", "gray20", "gray40","gray60", "gray80", "gray95")

for(k in 1:length(kernel.d)){

ver=getverticeshr(xkernel, percent=kernel.d[k])

for(i in 1:length(ver@polygons[[1]]@Polygons)){

polygon(ver@polygons[[1]]@Polygons[[i]]@coords, border=NA, col=cols[k])

kernel.areas[k]=kernel.areas[k]+areapl(ver@polygons[[1]]@Polygons[[i]]@coords)/(1000^2)

}

}

for(i in 1:length(savannah)){

polygon(savannah[[i]], border=NA, col=adjustcolor(col="yellow", alpha.f=0.4))

}

polygon(swamp[[10]], border=NA, col=adjustcolor(col="cadetblue4", alpha.f=0.4))

polygon(sea[[1]], border=NA, col=adjustcolor(col="lightsteelblue2", alpha.f=0.4))#### 3.1.3.1 Looking at the areas of each KDE isopleth (km2)

kernel.areas#### 3.1.3.2 Adding the location of Ozouga Research Camp to the map

camp.loc=c(x=553601, y=9765888)

points(x=camp.loc["x"], y=camp.loc["y"], pch=8, cex=1.2, lwd=1)#### 3.1.3.3 Adding the scale:arrows(x0=par()[["usr"]][1]+1000, x1=par()[["usr"]][1]+2000, y0=par()[["usr"]][3]+500, y1=par()[["usr"]][3]+500, code=3, angle=90, length=0.05)

text(x=par()[["usr"]][1]+1500, y=par()[["usr"]][3]+700, labels="1 km", cex=1.5)

#### 3.1.3.4 Adding the legend:

box.el=200

cols=c("gray20", "gray30", "gray60","gray80", "gray90", "gray95",

adjustcolor(col="yellow", alpha.f=0.4), adjustcolor(col="cadetblue4", alpha.f=0.4), adjustcolor(col="lightsteelblue2", alpha.f=0.4),"white", "black")

borders=rep("black", length(cols))

texts=c(paste(c(99,98, 95,80, 75, 50), "% ", sep=""), "Savannah & Beach", "Swamp", "Sea","Forest", "Camp") for(i in 1:length(texts)){

if(i<=10){

rect(xleft=par()[["usr"]][1]+0.5*box.el, xright=par()[["usr"]][1]+1.5*box.el,

ytop= par()[["usr"]][4]-box.el*0.5*i-box.el*(i-1),

ybottom=par()[["usr"]][4]-box.el*0.5*i-box.el*(i-1)-box.el,

col=cols[i], border="black")

}else{

points(x=par()[["usr"]][1]+box.el, y=par()[["usr"]][4]-box.el*0.5*i-box.el*(i-1)-box.el/2, pch=8)

}

text(x=par()[["usr"]][1]+1.5*box.el+50, y=par()[["usr"]][4]-box.el*0.5*i-box.el*(i-1)-box.el/2, labels=texts[i], adj=0, cex=0.8)

}

#### 3.1.3.5 Adding the title to the map

text(x=par()[["usr"]][1]+4500, y=par()[["usr"]][3]+10000, labels="KDE ", adj=0, cex=1.5)

## 3.1.4 Saving the map

savePlot(file="C:/Users/laura/Dropbox/Research/LoangoChimpanzeeProject/201909_intercommunity_study/2019refresh/202004_maps/202009_KDE_home_range_Rekambo_href_grid100_201701_201904.png", type="png", device=dev.cur())

dev.copy2pdf(file="C:/Users/laura/Dropbox/Research/LoangoChimpanzeeProject/201909_intercommunity_study/2019refresh/202004_maps/202009_KDE_home_range_Rekambo_href_grid100_201701_201904.pdf")####### 3.2 KDE (hLCSV) ######

###R session aborted in R and Rstuio when we tried to calculate KDE with hLSCV

## When I tried with less data (e.g. 2017 only), the session did not abort but received the error:

# "Error in .kernelUDs(SpatialPoints(x, proj4string = CRS(as.character(pfs1))), :

#'Calloc' could not allocate memory (2059563815 of 8 bytes)"

#I tried then with data of 1 month only and then the error was:

#In .kernelUDs(SpatialPoints(x, proj4string = CRS(as.character(pfs1))),

#The algorithm did not converge

#within the specified range of hlim: try to increase it

#This result didn''t change even if the limit was set to a million, which apparently is normal if the points are close together

# as they say here: https://ecosystems.psu.edu/research/labs/walter-lab/manual/home-range-estimation/link-to-pdf

# and here : https://animov.faunalia.narkive.com/NnG9nLKl/again-kernelud-and-lscv

#LSCV is better than href when relocations are very scarce (<1000)

# (e.g https://www.jstor.org/stable/3784605?casa_token=uzpe4OIFxFoAAAAA:MMgTtUujUcFjVcCWGAaHe4_E8Z5u-JKfJY7Gu2x_2gZm8grOLWVRerdVm3jysLzUGu9Ri7zjXrh3a0jP8dL9E2Xt1iNOx9Dic7iyWOx8rpAtNNmWJmoxZg&seq=1#metadata_info_tab_contents

#and https://www.jstor.org/stable/3802664?casa_token=4qerYWPXfTEAAAAA:_d7SdII6d8_4UBIyj1LJ02660v11e72CsV2KtmDMGMjBwMTgOBQGtpi9K1xgvRk2JZeQbdNCg_OmtOY6ubYtA7bmeuBQGJiIRo6kaH3WWrojoA6ljeIwNw&seq=1#metadata_info_tab_contents

#), but it fails for big sample sizes as in Hemson et al 2005 Are kernels the mustard? Data from global positioning

# system (GPS) collars suggests problems for kernel home-range analyses with least-squares cross-validation.

# Journal of Animal Ecology, 74: 455-463#The code that did not work is as follows:

# ### 3.2.1 Calculating KDE

# xkernel.LSCV=kernelUD(k.data, h="LSCV", grid=100,kern="bivnorm")

# ver.lscv=getverticeshr(xkernel.LSCV, percent=99)

# ### 3.2.2 Setting the isopleths to calculate the area of each

# kernel.d..LSCV=rev(c(50, 75, 80, 95,98,99))

# kernel.areas.LSCV=rep(0, length(kernel.d.LSCV))

# names(kernel.areas.LSCV)=kernel.d.LSCV

# ### 3.2.3 Plotting the KDE (hLSCV) on the map of the study site and calculating the areas

# par(mar=rep(0.2, 4))

# plot(getverticeshr(xkernel.LSCV, percent=99), asp=1, xlab="", ylab="",xlim=c(548350, 557873),ylim=c(9765030, 9774195), axes=T, xaxt="n", yaxt="n", bty="n", border=NA)

# cols=c("gray40", "gray50", "gray60","gray75", "gray85", "gray95")

# for(k in 1:length(kernel.d.LSCV)){

# ver=getverticeshr(xkernel.LSCV, percent=kernel.d.LSCV[k])

# for(i in 1:length(ver@polygons[[1]]@Polygons)){

# polygon(ver@polygons[[1]]@Polygons[[i]]@coords, border=NA, col=cols[k])

# kernel.areas.LSCV[k]=kernel.areas.LSCV[k]+areapl(ver@polygons[[1]]@Polygons[[i]]@coords)/(1000^2)

# }

# }

# for(i in 1:length(savannah)){

# polygon(savannah[[i]], border=NA, col=adjustcolor(col="yellow", alpha.f=0.4))

# }

# polygon(swamp[[10]], border=NA, col=adjustcolor(col="cadetblue4", alpha.f=0.4))

# polygon(sea[[1]], border=NA, col="lightsteelblue2")

#

#

# #### 3.2.3.1 Showing the calculated KDE areas of each isopleth (km2)

# kernel.areas.LSCV

#

# #### 3.2.3.2 Adding the location of Ozouga Research Camp to the map

# camp.loc=c(x=553601, y=9765888)

# points(x=camp.loc["x"], y=camp.loc["y"], pch=8, cex=1.2, lwd=1)

#

#

#

# #### 3.2.3.3 Adding the scale

# arrows(x0=par()[["usr"]][1]+3000, x1=par()[["usr"]][1]+4000, y0=par()[["usr"]][3]+500, y1=par()[["usr"]][3]+500, code=3, angle=90, length=0.05)

# text(x=par()[["usr"]][1]+3500, y=par()[["usr"]][3]+700, labels="1 km", cex=1.2)

#

#

# #### 3.2.3.4 Adding the legend

# box.el=200

# cols=c("gray40","gray50", "gray60","gray75", "gray85", "gray95",

# adjustcolor(col="yellow", alpha.f=0.4), adjustcolor(col="cadetblue4", alpha.f=0.4), "lightsteelblue2", "white","black")

# borders=rep("black", length(cols))

# texts=c(paste(c(99,95,80, 75, 50), "% KDE (hLSCV)", sep=""), "Savannah & Beach", "Swamp", "Sea", "Forest", "Camp")

#

# for(i in 1:length(texts)){

# if(i<=10){

# rect(xleft=par()[["usr"]][1]+12*box.el, xright=par()[["usr"]][1]+13*box.el,

# ytop= par()[["usr"]][4]-box.el*0.5*i-box.el*(i-1),

# ybottom=par()[["usr"]][4]-box.el*0.5*i-box.el*(i-1)-box.el,

# col=cols[i], border="black")

# }else{

# points(x=par()[["usr"]][1]+12.5*box.el, y=par()[["usr"]][4]-box.el*0.5*i-box.el*(i-1)-box.el/2, pch=8)

# }

# text(x=par()[["usr"]][1]+13.5*box.el+63, y=par()[["usr"]][4]-box.el*0.5*i-box.el*(i-1)-box.el/2, labels=texts[i], adj=0, cex=0.8)

# }

#### 3.2.3.4 Adding the title

# #text(x=par()[["usr"]][1]+4500, y=par()[["usr"]][3]+9000, labels="KDE (hLCSV)", adj=0, cex=1.5)

#

# ### 3.2.4 Saving the maps

# savePlot(file="C:/Users/laura/Dropbox/Research/LoangoChimpanzeeProject/201909_intercommunity_study/2019refresh/202004_maps/202009_KDE_home_range_Rekambo_hlscv_grid100_201701_201904.png", type="png", device=dev.cur())

#dev.copy2pdf(file="C:/Users/laura/Dropbox/Research/LoangoChimpanzeeProject/201909_intercommunity_study/2019refresh/202004_maps/202009_KDE_home_range_Rekambo_hlscv_grid100_201701_201904.png.pdf")

# ###############################################################################

#### 4. Calculating the home range with Biased Random Bridges (BRB) ######

################################################################################ 4.1 Preparing the data to create a ltraj object (a trayectory instead of the Spatial Points used in KDE and MCP)## 4.1.1 Date for ltraj needs to be in POSIXct format, so we have to transform it

date.bbmm <- as.POSIXct(strptime(alltracks$date,format="%d-%m-%Y %H:%M:%S"))## 4.1.2 Creating the ltraj object

ltraj=as.ltraj(xy = tracks, date = date.bbmm, id=F)# 4.2 Estimating D (diffusion coefficient)

BRB.likD(ltraj, Dr=c(0.1,10000), Tmax=120*60, Lmin=50, habitat = NULL, activity = NULL)#most likely D was 1.6# 4.3 Calculating the BRB

rek.brb <- BRB(ltraj, D=1.6, Tmax = 120*60, Lmin = 50, hmin=100,type="UD", grid = 100)# 4.4 Preparing the different isopleths to calculate their area

brb.cont=rev(c(50, 75, 80, 95,98,99))

brb.areas=rep(0, length(brb.cont))

names(brb.areas)=brb.cont# 4.4 Plotting the BRB home range over the map of the study site and calculation of the areas

par(mar=rep(0.2, 4))

plot(getverticeshr(rek.brb,percent=99,standardize = FALSE), asp=1, xlab="", ylab="",xlim=c(548350, 557873),ylim=c(9765030, 9774195), axes=F, xaxt="n", yaxt="n", bty="n", border=NA)

cols=c("gray0", "gray20", "gray40","gray60", "gray80", "gray95")

for(k in 1:length(brb.cont)){

ver.brb=getverticeshr(rek.brb, percent=brb.cont[k])

for(i in 1:length(ver.brb@polygons[[1]]@Polygons)){

polygon(ver.brb@polygons[[1]]@Polygons[[i]]@coords, border=NA, col=cols[k])

brb.areas[k]=brb.areas[k]+areapl(ver.brb@polygons[[1]]@Polygons[[i]]@coords)/(1000^2)

}

}

for(i in 1:length(savannah)){

polygon(savannah[[i]], border=NA, col=adjustcolor(col="yellow", alpha.f=0.4))

}

polygon(swamp[[10]], border=NA, col=adjustcolor(col="cadetblue4", alpha.f=0.4))

polygon(sea[[1]], border=NA, col= adjustcolor(col="lightsteelblue2", alpha.f=0.4))

## 4.4.1 Showing the area of each BRB isopleth

brb.areas## 4.4.2 Adding the location of the Ozouga Research Camp to the map

camp.loc=c(x=553601, y=9765888)

points(x=camp.loc["x"], y=camp.loc["y"], pch=8, cex=1.2, lwd=1)## 4.4.3 Adding the scale

arrows(x0=par()[["usr"]][1]+1000, x1=par()[["usr"]][1]+2000, y0=par()[["usr"]][3]+500, y1=par()[["usr"]][3]+500, code=3, angle=90, length=0.05)

text(x=par()[["usr"]][1]+1500, y=par()[["usr"]][3]+700, labels="1 km", cex=1.5)## 4.4.4 Adding the legend

box.el=200

cols=c("gray20", "gray30", "gray60","gray80", "gray90", "gray95",

adjustcolor(col="yellow", alpha.f=0.4), adjustcolor(col="cadetblue4", alpha.f=0.4), adjustcolor(col="lightsteelblue2", alpha.f=0.4),"white", "black")

borders=rep("black", length(cols))

texts=c(paste(c(99,98, 95,80, 75, 50), "% ", sep=""), "Savannah & Beach", "Swamp", "Sea","Forest", "Camp")for(i in 1:length(texts)){

if(i<=10){

rect(xleft=par()[["usr"]][1]+0.5*box.el, xright=par()[["usr"]][1]+1.5*box.el,

ytop= par()[["usr"]][4]-box.el*0.5*i-box.el*(i-1),

ybottom=par()[["usr"]][4]-box.el*0.5*i-box.el*(i-1)-box.el,

col=cols[i], border="black")

}else{

points(x=par()[["usr"]][1]+box.el, y=par()[["usr"]][4]-box.el*0.5*i-box.el*(i-1)-box.el/2, pch=8)

}

text(x=par()[["usr"]][1]+1.5*box.el+50, y=par()[["usr"]][4]-box.el*0.5*i-box.el*(i-1)-box.el/2, labels=texts[i], adj=0, cex=0.8)

}## 4.4.5 Adding the title to the map

text(x=par()[["usr"]][1]+4500, y=par()[["usr"]][3]+10000, labels="BRB", adj=0, cex=1.5)## 4.5 Saving the map

savePlot(file="C:/Users/laura/Dropbox/Research/LoangoChimpanzeeProject/201909_intercommunity_study/2019refresh/202004_maps/202009_BRB_Tmax120_Lmin50_hmin100_grid_100_201701_201904.png", type="png", device=dev.cur())

dev.copy2pdf(file="C:/Users/laura/Dropbox/Research/LoangoChimpanzeeProject/201909_intercommunity_study/2019refresh/202004_maps/202009_BRB_Tmax120_Lmin50_hmin100_grid_100_201701_201904.pdf")###############################################################################

#### 5. Comparing contours across the three different estimators ######

#######################################################################################

# 5.1 Comparing 99% contours

######### 5.1.1 Getting the contours of the KDE and BRB (99% MCP was obtained when calculating the home range)

ver.kde99=getverticeshr(xkernel, percent=99)

ver.brb99=getverticeshr(rek.brb, percent=99)# 5.1.2 Plotting the 99% contours over the study site map

par(mar=rep(0.2, 4))

plot(getverticeshr(rek.brb,percent=99,standardize = FALSE), asp=1, xlab="", ylab="",xlim=c(548350, 557873),ylim=c(9765030, 9774195), axes=F, xaxt="n", yaxt="n", bty="n", border=NA)

polygon(MCP[["99"]], border="green4", col=NA,lwd=4,lty=3)

for(i in 1:length(ver.kde99@polygons[[1]]@Polygons)){

polygon(ver.kde99@polygons[[1]]@Polygons[[i]]@coords, border="black", lwd=2,lty=2)

}

for(i in 1:length(ver.brb99@polygons[[1]]@Polygons)){

polygon(ver.brb99@polygons[[1]]@Polygons[[i]]@coords, border="blue", lwd=2)

}

for(i in 1:length(savannah)){

polygon(savannah[[i]], border=NA, col=adjustcolor(col="yellow", alpha.f=0.4))

}

polygon(swamp[[10]], border=NA, col=adjustcolor(col="cadetblue4", alpha.f=0.4))

polygon(sea[[1]], border=NA, col=adjustcolor(col="lightsteelblue2", alpha.f=0.4))## 5.1.2.1 Adding the location of the Ozouga Research Camp:

camp.loc=c(x=553601, y=9765888)

points(x=camp.loc["x"], y=camp.loc["y"], pch=8, cex=1.2, lwd=1)## 5.1.2.2 Adding the scale:

arrows(x0=par()[["usr"]][1]+1000, x1=par()[["usr"]][1]+2000, y0=par()[["usr"]][3]+500, y1=par()[["usr"]][3]+500, code=3, angle=90, length=0.05)

text(x=par()[["usr"]][1]+1500, y=par()[["usr"]][3]+700, labels="1 km", cex=1.5)

## 5.1.2.3 Adding the legend:

box.el=200

ltys=c(3,2,1)

lwds=c(4,2,2)

cols=c(NA,NA,NA,adjustcolor(col="yellow", alpha.f=0.4), adjustcolor(col="cadetblue4", alpha.f=0.4), adjustcolor(col="lightsteelblue2", alpha.f=0.4), "white", "black")

borders=c("green4", "black","blue", "black", "black","black" )

texts=c("MCP", "KDE", "BRB", "Savannah & Beach", "Swamp", "Sea","Forest", "Camp")

for(i in 1:length(texts)){

if(i<=3){

segments(x0=par()[["usr"]][1]+0.5*box.el, x1=par()[["usr"]][1]+2*box.el,

y0=par()[["usr"]][4]-box.el*0.5*i-box.el*(i-1)-box.el/2, y1=par()[["usr"]][4]-box.el*0.5*i-box.el*(i-1)-box.el/2, lwd=lwds[i], lty=ltys[i],col=borders[i])

}else if(i<=7){

rect(xleft=par()[["usr"]][1]+0.5*box.el, xright=par()[["usr"]][1]+1.5*box.el,

ytop= par()[["usr"]][4]-box.el*0.5*i-box.el*(i-1),

ybottom=par()[["usr"]][4]-box.el*0.5*i-box.el*(i-1)-box.el,

col=cols[i], border="black")

}else{

points(x=par()[["usr"]][1]+box.el, y=par()[["usr"]][4]-box.el*0.5*i-box.el*(i-1)-box.el/2, pch=8)

}

text(x=par()[["usr"]][1]+2.5*box.el+50, y=par()[["usr"]][4]-box.el*0.5*i-box.el*(i-1)-box.el/2, labels=texts[i], adj=0, cex=0.8)

}

## 5.1.2.4 Adding the map title

text(x=par()[["usr"]][1]+3500, y=par()[["usr"]][3]+10000, labels="99% Isopleths", adj=0, cex=1.5)# 5.1.3 Saving the map

savePlot(file="C:/Users/laura/Dropbox/Research/LoangoChimpanzeeProject/201909_intercommunity_study/2019refresh/202004_maps/202009_201701_201904_99_percent_contour_comparison.png", type="png", device=dev.cur())

dev.copy2pdf(file="C:/Users/laura/Dropbox/Research/LoangoChimpanzeeProject/201909_intercommunity_study/2019refresh/202004_maps/202009_201701_201904_99_percent_contour_comparison.pdf")

########

# 5.2 Comparing 75% contours

########

ver.kde75=getverticeshr(xkernel, percent=75)

ver.brb75=getverticeshr(rek.brb, percent=75)## 5.2.1 Plotting the 75% contours over the map of the study site

par(mar=rep(0.2, 4))

plot(getverticeshr(rek.brb,percent=99,standardize = FALSE), asp=1, xlab="", ylab="",xlim=c(548350, 557873),ylim=c(9765030, 9774195), axes=F, xaxt="n", yaxt="n", bty="n", border=NA)

polygon(MCP[["75"]], border="green4", col=NA, lwd=4,lty=3)

for(i in 1:length(ver.kde75@polygons[[1]]@Polygons)){

polygon(ver.kde75@polygons[[1]]@Polygons[[i]]@coords, border="black", lwd=2,lty=2)

}

for(i in 1:length(ver.brb75@polygons[[1]]@Polygons)){

polygon(ver.brb75@polygons[[1]]@Polygons[[i]]@coords, border="blue", lwd=2)

}

for(i in 1:length(savannah)){

polygon(savannah[[i]], border=NA, col=adjustcolor(col="yellow", alpha.f=0.4))

}

polygon(swamp[[10]], border=NA, col=adjustcolor(col="cadetblue4", alpha.f=0.4))

polygon(sea[[1]], border=NA, col=adjustcolor(col="lightsteelblue2", alpha.f=0.4))

### 5.2.2 Adding the location of the Ozouga Research Camp to the map

camp.loc=c(x=553601, y=9765888)

points(x=camp.loc["x"], y=camp.loc["y"], pch=8, cex=1.2, lwd=1)

### 5.2.3 Adding the scale

arrows(x0=par()[["usr"]][1]+1000, x1=par()[["usr"]][1]+2000, y0=par()[["usr"]][3]+500, y1=par()[["usr"]][3]+500, code=3, angle=90, length=0.05)

text(x=par()[["usr"]][1]+1500, y=par()[["usr"]][3]+700, labels="1 km", cex=1.5)

### 5.2.4 Adding the legend

box.el=200

ltys=c(3,2,1)

lwds=c(4,2,2)

cols=c(NA,NA,NA,adjustcolor(col="yellow", alpha.f=0.4), adjustcolor(col="cadetblue4", alpha.f=0.4), adjustcolor(col="lightsteelblue2", alpha.f=0.4), "white", "black")

borders=c("green4", "black","blue", "black", "black","black" )

texts=c("MCP", "KDE", "BRB", "Savannah & Beach", "Swamp", "Sea","Forest", "Camp")

for(i in 1:length(texts)){

if(i<=3){

segments(x0=par()[["usr"]][1]+0.5*box.el, x1=par()[["usr"]][1]+2*box.el,

y0=par()[["usr"]][4]-box.el*0.5*i-box.el*(i-1)-box.el/2, y1=par()[["usr"]][4]-box.el*0.5*i-box.el*(i-1)-box.el/2, lwd=lwds[i], lty=ltys[i],col=borders[i])

}else if(i<=7){

rect(xleft=par()[["usr"]][1]+0.5*box.el, xright=par()[["usr"]][1]+1.5*box.el,

ytop= par()[["usr"]][4]-box.el*0.5*i-box.el*(i-1),

ybottom=par()[["usr"]][4]-box.el*0.5*i-box.el*(i-1)-box.el,

col=cols[i], border="black")

}else{

points(x=par()[["usr"]][1]+box.el, y=par()[["usr"]][4]-box.el*0.5*i-box.el*(i-1)-box.el/2, pch=8)

}

text(x=par()[["usr"]][1]+2.5*box.el+50, y=par()[["usr"]][4]-box.el*0.5*i-box.el*(i-1)-box.el/2, labels=texts[i], adj=0, cex=0.8)

}

##5.2.5 Adding the title to the map

text(x=par()[["usr"]][1]+3500, y=par()[["usr"]][3]+10000, labels="75% Isopleths", adj=0, cex=1.5)#5.3 Saving the map

savePlot(file="C:/Users/laura/Dropbox/Research/LoangoChimpanzeeProject/201909_intercommunity_study/2019refresh/202004_maps/202008_201701_201904_75_percent_contour_comparison2.png", type="png", device=dev.cur())

dev.copy2pdf(file="C:/Users/laura/Dropbox/Research/LoangoChimpanzeeProject/201909_intercommunity_study/2019refresh/202004_maps/202008_201701_201904_75_percent_contour_comparison.pdf")########

# 5.3 Comparing 50% contours

########

ver.kde50=getverticeshr(xkernel, percent=50)

ver.brb50=getverticeshr(rek.brb, percent=50)## 5.3.1 Plotting the 50% contours over the map of the study site

par(mar=rep(0.2, 4))

plot(getverticeshr(rek.brb,percent=99,standardize = FALSE), asp=1, xlab="", ylab="",xlim=c(548350, 557873),ylim=c(9765030, 9774195), axes=F, xaxt="n", yaxt="n", bty="n", border=NA)

polygon(MCP[["50"]], border="green4", col=NA, lwd=4,lty=3)

for(i in 1:length(ver.kde50@polygons[[1]]@Polygons)){

polygon(ver.kde50@polygons[[1]]@Polygons[[i]]@coords, border="black", lwd=2,lty=2)

}

for(i in 1:length(ver.brb50@polygons[[1]]@Polygons)){

polygon(ver.brb50@polygons[[1]]@Polygons[[i]]@coords, border="blue", lwd=2)

}

for(i in 1:length(savannah)){

polygon(savannah[[i]], border=NA, col=adjustcolor(col="yellow", alpha.f=0.4))

}

polygon(swamp[[10]], border=NA, col=adjustcolor(col="cadetblue4", alpha.f=0.4))

polygon(sea[[1]], border=NA, col=adjustcolor(col="lightsteelblue2", alpha.f=0.4))## 5.3.2 Adding the location of the Ozouga Research Camp to the map

camp.loc=c(x=553601, y=9765888)

points(x=camp.loc["x"], y=camp.loc["y"], pch=8, cex=1.2, lwd=1)## 5.3.3 Adding the scale

arrows(x0=par()[["usr"]][1]+1000, x1=par()[["usr"]][1]+2000, y0=par()[["usr"]][3]+500, y1=par()[["usr"]][3]+500, code=3, angle=90, length=0.05)

text(x=par()[["usr"]][1]+1500, y=par()[["usr"]][3]+700, labels="1 km", cex=1.5)## 5.3.4 Adding the legend

box.el=200

ltys=c(3,2,1)

lwds=c(4,2,2)

cols=c(NA,NA,NA,adjustcolor(col="yellow", alpha.f=0.4), adjustcolor(col="cadetblue4", alpha.f=0.4), adjustcolor(col="lightsteelblue2", alpha.f=0.4), "white", "black")

borders=c("green4", "black","blue", "black", "black","black" )

texts=c("MCP", "KDE", "BRB", "Savannah & Beach", "Swamp", "Sea","Forest", "Camp")

for(i in 1:length(texts)){

if(i<=3){

segments(x0=par()[["usr"]][1]+0.5*box.el, x1=par()[["usr"]][1]+2*box.el,

y0=par()[["usr"]][4]-box.el*0.5*i-box.el*(i-1)-box.el/2, y1=par()[["usr"]][4]-box.el*0.5*i-box.el*(i-1)-box.el/2, lwd=lwds[i], lty=ltys[i],col=borders[i])

}else if(i<=7){

rect(xleft=par()[["usr"]][1]+0.5*box.el, xright=par()[["usr"]][1]+1.5*box.el,

ytop= par()[["usr"]][4]-box.el*0.5*i-box.el*(i-1),

ybottom=par()[["usr"]][4]-box.el*0.5*i-box.el*(i-1)-box.el,

col=cols[i], border="black")

}else{

points(x=par()[["usr"]][1]+box.el, y=par()[["usr"]][4]-box.el*0.5*i-box.el*(i-1)-box.el/2, pch=8)

}

text(x=par()[["usr"]][1]+2.5*box.el+50, y=par()[["usr"]][4]-box.el*0.5*i-box.el*(i-1)-box.el/2, labels=texts[i], adj=0, cex=0.8)

}

## 5.3.5 Adding the title to the map

text(x=par()[["usr"]][1]+3500, y=par()[["usr"]][3]+10000, labels="50% Isopleths", adj=0, cex=1.5)# 5.4 Saving the map

savePlot(file="C:/Users/laura/Dropbox/Research/LoangoChimpanzeeProject/201909_intercommunity_study/2019refresh/202004_maps/202009_201701_201904_50_percent_contour_comparison2.png", type="png", device=dev.cur())

dev.copy2pdf(file="C:/Users/laura/Dropbox/Research/LoangoChimpanzeeProject/201909_intercommunity_study/2019refresh/202004_maps/202009_201701_201904_50_percent_contour_comparison2.pdf")

###############################################################################

#### 6. Calculating the Area Under the Curve ######

###############################################################################

#The following R code comes originally from:

#Cumming GS, Cornélis D. Quantitative comparison and selection of home range metrics for telemetry

#data. Divers Distrib. 2012; 18(11):1057 - 65. doi: 10.1111/j.1472-4642.2012.00908.x

###########

# 6.1 AUC KDE (href)

############ 6.1.1 Computing volumes

udvol.k<-getvolumeUD(xkernel, standardize = FALSE)

udvol.kernel<-estUDm2spixdf(udvol.k) # conversion of volume contours to format SpatialPixelsDataFrame (necessary for count.cells)

## 6.1.2 Counting locations within volume raster

nlocrast_kernel<-count.points(xyALL,udvol.kernel)

kerneldata <- udvol.kernel@data # vector containing volume contour(=predicted) values

pointdata.kernel <- nlocrast_kernel@data$x

pointdata.kernel <- ifelse(pointdata.kernel>=1,1,0) # vector containing location (=actual) values ## 6.1.3 Calculating AUC

par(mar=rep(6, 4))

print(AUC_kernel <- colAUC(kerneldata, pointdata.kernel, plotROC=TRUE, alg=c("Wilcoxon","ROC"))) ###########

# 6.2 AUC BRB

############ 6.2.1 Computing volumes

udvol.brb<-getvolumeUD(rek.brb, standardize = TRUE)## 6.2.2 Counting locations within volume raster

nlocrast_brb<-count.points(xyALL,udvol.brb)

brbdata <- udvol.brb@data$n #vector containing volume contour(=predicted) values

pointdata.brb <- nlocrast_brb@data$x

pointdata.brb <- ifelse(pointdata.brb>=1,1,0) # vector containing location (=actual) values ## 6.2.3 Calculating AUC

par(mar=rep(6, 4))

print(AUC_brb <- colAUC(brbdata, pointdata.brb, plotROC=TRUE, alg=c("Wilcoxon","ROC")))

###########

# 6.3 AUC MCP

##########

## 6.3.1 Creating grid 100X100 (Modified from Cumming & Cornelis 2012 Divers Distrib.)

RESO <- 100 # grid resolution (m)

BUFF <- 100 # grid extent (m) (buffer around location extremes)

XMIN <- RESO*(round(((min(xyALL$long)-BUFF)/RESO),0))

YMIN <- RESO*(round(((min(xyALL$lat)-BUFF)/RESO),0))

XMAX <- XMIN+RESO*(round(((max(xyALL$long)+BUFF-XMIN)/RESO),0))

YMAX <- YMIN+RESO*(round(((max(xyALL$lat)+BUFF-YMIN)/RESO),0))

NRW <- ((YMAX-YMIN)/RESO)

NCL <- ((XMAX-XMIN)/RESO)## 6.3.2 Transforming the 100x100 grid into Spatial Pixels

refgrid<-raster(nrows=NRW, ncols=NCL, xmn=XMIN, xmx=XMAX, ymn=YMIN, ymx=YMAX)

refgrid<-as(refgrid,"SpatialPixels")## 6.3.3 Calculating 100% MCP (it was inside a function originally and cannot be found on its own)

mcp.rek<- mcp(xyALL)

## 6.3.4 Transforming the MCP home range into Spatial Pixel Data Frame fitted to the 100x100m grid

mcp.rek1<-hr.rast(mcp.rek, refgrid)## 6.3.5 Counting locations within volume rasters

nlocrast_mcp<-count.points(xyALL,mcp.rek1)

mcp.rek1@data$a[is.na(mcp.rek1@data$a)] <- 0 #Pixeles not included in MCP were reported as NA, giving problems later on. Reclassified to zero

mcpdata <- mcp.rek1@data$a # vector containing volume contour(=predicted) values

pointdata.mcp <- nlocrast_mcp@data$x

pointdata.mcp <- ifelse(pointdata.mcp>=1,1,0) # vector containing location (=actual) values ## 6.3.6 Calculating AUC (You will likely need to close the R Graphics window before running this or it will not work)

par(mar=rep(6,4))

print(AUC_mcp <- colAUC(mcpdata, pointdata.mcp, plotROC=TRUE, alg=c("Wilcoxon","ROC")))

#####################################################################################

#### 7. Comparing the BRB home range calculated with tmax=120 minutes VS tmax 30 minutes ######

###################################################################################### 7.1 Recalculating the home range by BRB using 30 minutes as Tmaxrek.brb.tmax30 <- BRB(ltraj, D=1.6, Tmax = 30*60, Lmin = 50, hmin=100,type="UD", grid = 100)

# 7.2 Plotting and calculating the BRB when Tmax=30

brb.cont.tmax30=rev(c(50, 75, 80, 95,98,99))

brb.areas.tmax30=rep(0, length(brb.cont.tmax30))

names(brb.areas.tmax30)=brb.cont.tmax30par(mar=rep(0.2, 4))

plot(getverticeshr(rek.brb.tmax30,percent=99,standardize = FALSE), asp=1, xlab="", ylab="",xlim=c(548350, 557873),ylim=c(9765030, 9774195), axes=F, xaxt="n", yaxt="n", bty="n", border=NA)

cols=c("gray0", "gray20", "gray40","gray60", "gray80", "gray95")

for(k in 1:length(brb.cont.tmax30)){

ver.brb.tmax30=getverticeshr(rek.brb.tmax30, percent=brb.cont.tmax30[k])

for(i in 1:length(ver.brb.tmax30@polygons[[1]]@Polygons)){

polygon(ver.brb.tmax30@polygons[[1]]@Polygons[[i]]@coords, border=NA, col=cols[k])

brb.areas.tmax30[k]=brb.areas.tmax30[k]+areapl(ver.brb.tmax30@polygons[[1]]@Polygons[[i]]@coords)/(1000^2)

}

}

for(i in 1:length(savannah)){

polygon(savannah[[i]], border=NA, col=adjustcolor(col="yellow", alpha.f=0.4))

}

polygon(swamp[[10]], border=NA, col=adjustcolor(col="cadetblue4", alpha.f=0.4))

polygon(sea[[1]], border=NA, col= adjustcolor(col="lightsteelblue2", alpha.f=0.4))

# 7.3 Showing the area of the resulting isopleths (km2)brb.areas.tmax30

# 7.4 Comparing 95% and 50% contours of BRB Tmax=30 and BRB Tmax=120

ver.brb95=getverticeshr(rek.brb, percent=95)

ver.brb50=getverticeshr(rek.brb, percent=75)

ver.brb95.tmax30=getverticeshr(rek.brb.tmax30, percent=95)

ver.brb50.tmax30=getverticeshr(rek.brb.tmax30, percent=75)

### 7.4.1 Plotting the 95% and 50% contours of both BRB

par(mar=rep(0.2, 4))

plot(getverticeshr(rek.brb,percent=99,standardize = FALSE), asp=1, xlab="", ylab="",xlim=c(548350, 557873),ylim=c(9765030, 9774195), axes=F, xaxt="n", yaxt="n", bty="n", border=NA)

for(i in 1:length(ver.brb95@polygons[[1]]@Polygons)){

polygon(ver.brb95@polygons[[1]]@Polygons[[i]]@coords, border="black", lwd=2, lty=1)

}

for(i in 1:length(ver.brb50@polygons[[1]]@Polygons)){

polygon(ver.brb50@polygons[[1]]@Polygons[[i]]@coords, border="black", lwd=2, lty=2)

}

for(i in 1:length(ver.brb95.tmax30@polygons[[1]]@Polygons)){

polygon(ver.brb95.tmax30@polygons[[1]]@Polygons[[i]]@coords, border="green4", lwd=2, lty=1)

}

for(i in 1:length(ver.brb50.tmax30@polygons[[1]]@Polygons)){

polygon(ver.brb50.tmax30@polygons[[1]]@Polygons[[i]]@coords, border="green4", lwd=2, lty=2)

}for(i in 1:length(savannah)){

polygon(savannah[[i]], border=NA, col=adjustcolor(col="yellow", alpha.f=0.4))

}

polygon(swamp[[10]], border=NA, col=adjustcolor(col="cadetblue4", alpha.f=0.4))

polygon(sea[[1]], border=NA, col=adjustcolor(col="lightsteelblue2", alpha.f=0.4))

### 7.4.2 Adding the location of the Ozouga Research Camp to the map

camp.loc=c(x=553601, y=9765888)

points(x=camp.loc["x"], y=camp.loc["y"], pch=8, cex=1.2, lwd=1)### 7.4.3 Adding the scale

arrows(x0=par()[["usr"]][1]+1000, x1=par()[["usr"]][1]+2000, y0=par()[["usr"]][3]+500, y1=par()[["usr"]][3]+500, code=3, angle=90, length=0.05)

text(x=par()[["usr"]][1]+1500, y=par()[["usr"]][3]+700, labels="1 km", cex=1.5)### 7.4.4 Adding the legend

box.el=200

cols=c(NA,NA,NA,NA,adjustcolor(col="yellow", alpha.f=0.4), adjustcolor(col="cadetblue4", alpha.f=0.4), adjustcolor(col="lightsteelblue2", alpha.f=0.4),"white", "black")

borders=c("black", "black","green4","green4","black", "black","black")

texts=c("BRB 95% (Tmax=120)", "BRB 75% (Tmax=120)", "BRB 95% (Tmax=30)", "BRB 75% (Tmax=30)","Savannah & Beach", "Swamp", "Sea","Forest", "Camp")

lty=c(1,2,1,2)for(i in 1:length(texts)){

if(i<=4){

segments(x0=par()[["usr"]][1]+0.5*box.el, x1=par()[["usr"]][1]+2*box.el,

y0=par()[["usr"]][4]-box.el*0.5*i-box.el*(i-1)-box.el/2, y1=par()[["usr"]][4]-box.el*0.5*i-box.el*(i-1)-box.el/2,lty=lty[i], lwd=2, col=borders[i])

}else if(i<=8){

rect(xleft=par()[["usr"]][1]+0.5*box.el, xright=par()[["usr"]][1]+1.5*box.el,

ytop= par()[["usr"]][4]-box.el*0.5*i-box.el*(i-1),

ybottom=par()[["usr"]][4]-box.el*0.5*i-box.el*(i-1)-box.el,

col=cols[i], border="black")

}else{

points(x=par()[["usr"]][1]+box.el, y=par()[["usr"]][4]-box.el*0.5*i-box.el*(i-1)-box.el/2, pch=8)

}

text(x=par()[["usr"]][1]+2.5*box.el+50, y=par()[["usr"]][4]-box.el*0.5*i-box.el*(i-1)-box.el/2, labels=texts[i], adj=0, cex=0.8)

}# 7.5 Saving the map

savePlot(file="C:/Users/laura/Dropbox/Research/LoangoChimpanzeeProject/201909_intercommunity_study/2019refresh/202004_maps/202007_201701_201904_contour_comparison_BRBtmax120_and_BRBtmax30.png", type="png", device=dev.cur())

dev.copy2pdf(file="C:/Users/laura/Dropbox/Research/LoangoChimpanzeeProject/201909_intercommunity_study/2019refresh/202004_maps/202007_201701_201904_contour_comparison_BRBtmax120_and_BRBtmax30.pdf")#############################################################################

#### 8. Calculating and mapping the home range of the Rekambo community in 2017

#######################################################################################

# 8.1 Preparing the data for analyses

#########

## 8.1.1 Preparing the coordinates

utm.coords.2017=WGS.to.UTM(long=tracks.2017$lon, lat=tracks.2017$lat, utm.zone=utm.zone(mean(tracks.2017$lon), mean(tracks.2017$lat)), avoid.negatives=T, return.zone=F)

tracks2017=utm.coords.2017xy2017=SpatialPoints(coords=tracks2017[, c("long", "lat")])## 8.1.2 Counting amount of data used in the calculationstracks.2017$yyyymmdd<-as.factor(tracks.2017$yyyymmdd)

str(tracks.2017)#No obs=No locations= 221981

#$yyyymmdd= No. obs. days=261## 8.1.3 Calculating observation time included in the sample

#We will calculate the observation time with the calculation on lag. We will need to remove any with a

#lag >30min, which would be joining different tracks, so time in which there was no chimpanzee observationlags2017<- tracks.2017[tracks.2017$lag <= 30,]

lags2017<- lags2017$lag/60

str(lags2017)

sum(lags2017)#2329.325h approx

## 8.1.4 Creating ltraj object to calculate BRB

date.bbmm.2017 <- as.POSIXct(strptime(tracks.2017$date,format="%d-%m-%Y %H:%M:%S"))

ltraj.2017=as.ltraj(xy =tracks2017, date =date.bbmm.2017, id=F)

############### 8.2 MCP 2017 ##############

## Calculating the MCPto.get=c(100, 99, 98, 95, 80, 75, 50)

MCP2017=vector("list", length(to.get))

names(MCP2017)=to.get

for(i in 1:length(to.get)){

xx2017=mcp(xy2017, percent=to.get[i])

MCP2017[[i]]=xx2017@polygons[[1]]@Polygons[[1]]@coords

} ## Calculating the areas of each MCP isopleth (2017)

MCP.areas.2017=unlist(lapply(MCP2017, areapl))

MCP.areas.2017=MCP.areas.2017/(1000^2)

names(MCP.areas.2017)=to.get

MCP.areas.2017

##Plotting the MCP 2017 on the map of the study site

par(mar=rep(0.2, 4))

plot(as.matrix(MCP2017[["100"]]), asp=1, xlab="", ylab="",xlim=c(548350, 557873),ylim=c(9765030, 9774195), axes=T, type="n", xaxt="n", yaxt="n", bty="n")

cols=c("mediumaquamarine", "gray0", "gray20", "gray40","gray60", "gray80", "gray95")

for(i in 1:length(to.get)){

polygon(MCP2017[[i]], border=NA, col=cols[i])

}

for(i in 1:length(savannah)){

polygon(savannah[[i]], border=NA, col=adjustcolor(col="yellow", alpha.f=0.4))

}

polygon(swamp[[10]], border=NA, col=adjustcolor(col="cadetblue4", alpha.f=0.4))

polygon(sea[[1]], border=NA, col=adjustcolor(col="lightsteelblue2", alpha.f=0.4))

# Adding the location of the Ozouga Research Camp to the map

camp.loc=c(x=553601, y=9765888)

points(x=camp.loc["x"], y=camp.loc["y"], pch=8, cex=1.2, lwd=1)# Adding the scale

arrows(x0=par()[["usr"]][1]+1000, x1=par()[["usr"]][1]+2000, y0=par()[["usr"]][3]+500, y1=par()[["usr"]][3]+500, code=3, angle=90, length=0.05)

text(x=par()[["usr"]][1]+1500, y=par()[["usr"]][3]+700, labels="1 km",cex=1.5)# Adding the legend

box.el=200

cols=c("mediumaquamarine", "gray0", "gray20", "gray40","gray60", "gray80", "gray95",

adjustcolor(col="yellow", alpha.f=0.4), adjustcolor(col="cadetblue4", alpha.f=0.4), adjustcolor(col="lightsteelblue2", alpha.f=0.4), "white", "black")

borders=rep("black", length(cols))

texts=c(paste(c(100, 99, 98, 95,80, 75, 50), "%", sep=""), "Savannah & Beach", "Swamp", "Sea", "Forest", "Camp")for(i in 1:length(texts)){

if(i<=11){

rect(xleft=par()[["usr"]][1]+0.5*box.el, xright=par()[["usr"]][1]+1.5*box.el,

ytop= par()[["usr"]][4]-box.el*0.5*i-box.el*(i-1),

ybottom=par()[["usr"]][4]-box.el*0.5*i-box.el*(i-1)-box.el,

col=cols[i], border="black")

}else{

points(x=par()[["usr"]][1]+box.el, y=par()[["usr"]][4]-box.el*0.5*i-box.el*(i-1)-box.el/2, pch=8)

}

text(x=par()[["usr"]][1]+1.5*box.el+50, y=par()[["usr"]][4]-box.el*0.5*i-box.el*(i-1)-box.el/2, labels=texts[i], adj=0, cex=0.8)

}

# Adding the title to the map

text(x=par()[["usr"]][1]+5000, y=par()[["usr"]][3]+10000, labels="MCP 2017", adj=0, cex=1.5)## 8.2.5 Saving the map

savePlot(file="C:/Users/laura/Dropbox/Research/LoangoChimpanzeeProject/201909_intercommunity_study/2019refresh/202004_maps/202009_MCP_home_range_Rekambo_2017.png", type="png", device=dev.cur())

dev.copy2pdf(file="C:/Users/laura/Dropbox/Research/LoangoChimpanzeeProject/201909_intercommunity_study/2019refresh/202004_maps/202009_MCP_home_range_Rekambo_2017.pdf")############### 8.3 KDE (href) 2017 ################ 8.3.1 Calculating KDE

k.data.2017=SpatialPointsDataFrame(coords=tracks2017, data=data.frame(id=rep("a", nrow(tracks2017))))

xkernel2017=kernelUD(k.data.2017, h="href", grid=100)xkernel2017[[1]]@h #showing the value of h## 8.3.2 Plotting and calculating the areas of the KDE 2017

kernel.d=rev(c(50, 75, 80, 95,98,99))

kernel.areas.2017=rep(0, length(kernel.d))

names(kernel.areas.2017)=kernel.d

par(mar=rep(0.2, 4))

plot(getverticeshr(xkernel2017, percent=99), asp=1, xlab="", ylab="",xlim=c(548350, 557873),ylim=c(9765030, 9774195), axes=F, xaxt="n", yaxt="n", bty="n", border=NA)

cols=c("gray0", "gray20", "gray40","gray60", "gray80", "gray95")

for(k in 1:length(kernel.d)){

ver=getverticeshr(xkernel2017, percent=kernel.d[k])

for(i in 1:length(ver@polygons[[1]]@Polygons)){

polygon(ver@polygons[[1]]@Polygons[[i]]@coords, border=NA, col=cols[k])

kernel.areas.2017[k]=kernel.areas.2017[k]+areapl(ver@polygons[[1]]@Polygons[[i]]@coords)/(1000^2)

}

}

for(i in 1:length(savannah)){

polygon(savannah[[i]], border=NA, col=adjustcolor(col="yellow", alpha.f=0.4))

}

polygon(swamp[[10]], border=NA, col=adjustcolor(col="cadetblue4", alpha.f=0.4))

polygon(sea[[1]], border=NA, col=adjustcolor(col="lightsteelblue2", alpha.f=0.4))# Calculate KDE areas (km2)

kernel.areas.2017# Adding the location of Ozouga Research Camp to the map

camp.loc=c(x=553601, y=9765888)

points(x=camp.loc["x"], y=camp.loc["y"], pch=8, cex=1.2, lwd=1)# Adding the scale

arrows(x0=par()[["usr"]][1]+1000, x1=par()[["usr"]][1]+2000, y0=par()[["usr"]][3]+500, y1=par()[["usr"]][3]+500, code=3, angle=90, length=0.05)

text(x=par()[["usr"]][1]+1500, y=par()[["usr"]][3]+700, labels="1 km", cex=1.5)

# Adding the legend

box.el=200

cols=c("gray20", "gray30", "gray60","gray80", "gray90", "gray95",

adjustcolor(col="yellow", alpha.f=0.4), adjustcolor(col="cadetblue4", alpha.f=0.4), adjustcolor(col="lightsteelblue2", alpha.f=0.4),"white", "black")

borders=rep("black", length(cols))

texts=c(paste(c(99,98, 95,80, 75, 50), "% ", sep=""), "Savannah & Beach", "Swamp", "Sea","Forest", "Camp")for(i in 1:length(texts)){

if(i<=10){

rect(xleft=par()[["usr"]][1]+0.5*box.el, xright=par()[["usr"]][1]+1.5*box.el,

ytop= par()[["usr"]][4]-box.el*0.5*i-box.el*(i-1),

ybottom=par()[["usr"]][4]-box.el*0.5*i-box.el*(i-1)-box.el,

col=cols[i], border="black")

}else{

points(x=par()[["usr"]][1]+box.el, y=par()[["usr"]][4]-box.el*0.5*i-box.el*(i-1)-box.el/2, pch=8)

}

text(x=par()[["usr"]][1]+1.5*box.el+50, y=par()[["usr"]][4]-box.el*0.5*i-box.el*(i-1)-box.el/2, labels=texts[i], adj=0, cex=0.8)

}

# Adding the title to the maptext(x=par()[["usr"]][1]+4500, y=par()[["usr"]][3]+10000, labels="KDE 2017", adj=0, cex=1.5)#Saving the map

savePlot(file="C:/Users/laura/Dropbox/Research/LoangoChimpanzeeProject/201909_intercommunity_study/2019refresh/202004_maps/202009_KDE_home_ramge_Rekambo_2017.png", type="png", device=dev.cur())

dev.copy2pdf(file="C:/Users/laura/Dropbox/Research/LoangoChimpanzeeProject/201909_intercommunity_study/2019refresh/202004_maps/202009_KDE_home_ramge_Rekambo_2017.pdf")

############### 8.4 BRB 2017 ############### Calculating the BRB

rek.brb.2017 <- BRB(ltraj.2017, D = 1.6, Tmax = 120*60, Lmin = 50, hmin=100,type="UD", grid = 100)

# Plotting the BRB of 2017 over the map of the site and calculating areas

brb.cont=rev(c(50, 75, 80, 95,98,99))

brb.areas.2017=rep(0, length(brb.cont))

names(brb.areas.2017)=brb.contpar(mar=rep(0.2, 4))

plot(getverticeshr(rek.brb.2017,percent=99,standardize = FALSE), asp=1, xlab="", ylab="",xlim=c(548350, 557873),ylim=c(9765030, 9774195), axes=F, xaxt="n", yaxt="n", bty="n", border=NA)

cols=c("gray0", "gray20", "gray40","gray60", "gray80", "gray95")

for(k in 1:length(brb.cont)){

ver.brb.2017=getverticeshr(rek.brb.2017, percent=brb.cont[k])

for(i in 1:length(ver.brb.2017@polygons[[1]]@Polygons)){

polygon(ver.brb.2017@polygons[[1]]@Polygons[[i]]@coords, border=NA, col=cols[k])

brb.areas.2017[k]=brb.areas.2017[k]+areapl(ver.brb.2017@polygons[[1]]@Polygons[[i]]@coords)/(1000^2)

}

}

for(i in 1:length(savannah)){

polygon(savannah[[i]], border=NA, col=adjustcolor(col="yellow", alpha.f=0.4))

}

polygon(swamp[[10]], border=NA, col=adjustcolor(col="cadetblue4", alpha.f=0.4))

polygon(sea[[1]], border=NA, col=adjustcolor(col="lightsteelblue2", alpha.f=0.4))

##Home range area (km2)

brb.areas.2017# Adding the location of Ozouga Research Camp to the map

camp.loc=c(x=553601, y=9765888)

points(x=camp.loc["x"], y=camp.loc["y"], pch=8, cex=1.2, lwd=1)# Adding the scale

arrows(x0=par()[["usr"]][1]+1000, x1=par()[["usr"]][1]+2000, y0=par()[["usr"]][3]+500, y1=par()[["usr"]][3]+500, code=3, angle=90, length=0.05)

text(x=par()[["usr"]][1]+1500, y=par()[["usr"]][3]+700, labels="1 km", cex=1.5)

# Adding the legend

box.el=200

cols=c("gray20", "gray30", "gray60","gray80", "gray90", "gray95",

adjustcolor(col="yellow", alpha.f=0.4), adjustcolor(col="cadetblue4", alpha.f=0.4), adjustcolor(col="lightsteelblue2", alpha.f=0.4),"white", "black")

borders=rep("black", length(cols))

texts=c(paste(c(99,98, 95,80, 75, 50), "% ", sep=""), "Savannah & Beach", "Swamp", "Sea","Forest", "Camp")for(i in 1:length(texts)){

if(i<=10){

rect(xleft=par()[["usr"]][1]+0.5*box.el, xright=par()[["usr"]][1]+1.5*box.el,

ytop= par()[["usr"]][4]-box.el*0.5*i-box.el*(i-1),

ybottom=par()[["usr"]][4]-box.el*0.5*i-box.el*(i-1)-box.el,

col=cols[i], border="black")

}else{

points(x=par()[["usr"]][1]+box.el, y=par()[["usr"]][4]-box.el*0.5*i-box.el*(i-1)-box.el/2, pch=8)

}

text(x=par()[["usr"]][1]+1.5*box.el+50, y=par()[["usr"]][4]-box.el*0.5*i-box.el*(i-1)-box.el/2, labels=texts[i], adj=0, cex=0.8)

}

# Adding the title to the map

text(x=par()[["usr"]][1]+4500, y=par()[["usr"]][3]+10000, labels="BRB 2017", adj=0, cex=1.5)#Saving the map

savePlot(file="C:/Users/laura/Dropbox/Research/LoangoChimpanzeeProject/201909_intercommunity_study/2019refresh/202004_maps/202008_BRB_Rekambo_2017.png", type="png", device=dev.cur())

dev.copy2pdf(file="C:/Users/laura/Dropbox/Research/LoangoChimpanzeeProject/201909_intercommunity_study/2019refresh/202004_maps/202008_BRB_Rekambo_2017.pdf")############### 8.5 Comparing the 95% and 50% contours of 2017 for the three estimators ##############

####

# 8.5.1. Comparing the 95% contours of 2017

###

ver.kde95.2017=getverticeshr(xkernel2017, percent=95)

ver.brb.2017=getverticeshr(rek.brb.2017, percent=95)##plot:

par(mar=rep(0.2, 4))

plot(getverticeshr(rek.brb.2017,percent=99,standardize = FALSE), asp=1, xlab="", ylab="",xlim=c(548350, 557873),ylim=c(9765030, 9774195), axes=F, xaxt="n", yaxt="n", bty="n", border=NA)

polygon(MCP2017[["95"]], border="green4", col=NA, lwd=4,lty=3)

for(i in 1:length(ver.kde95.2017@polygons[[1]]@Polygons)){

polygon(ver.kde95.2017@polygons[[1]]@Polygons[[i]]@coords, border="black", lwd=2,lty=2)

}

for(i in 1:length(ver.brb.2017@polygons[[1]]@Polygons)){

polygon(ver.brb.2017@polygons[[1]]@Polygons[[i]]@coords, border="blue", col=NA, lty=1, lwd=2)

}

for(i in 1:length(savannah)){

polygon(savannah[[i]], border=NA, col=adjustcolor(col="yellow", alpha.f=0.4))

}

polygon(swamp[[10]], border=NA, col=adjustcolor(col="cadetblue4", alpha.f=0.4))

polygon(sea[[1]], border=NA, col=adjustcolor(col="lightsteelblue2", alpha.f=0.4))

## Adding the location of the Ozouga Research Camp

camp.loc=c(x=553601, y=9765888)

points(x=camp.loc["x"], y=camp.loc["y"], pch=8, cex=1.2, lwd=1)## Adding the scale

arrows(x0=par()[["usr"]][1]+1000, x1=par()[["usr"]][1]+2000, y0=par()[["usr"]][3]+500, y1=par()[["usr"]][3]+500, code=3, angle=90, length=0.05)

text(x=par()[["usr"]][1]+1500, y=par()[["usr"]][3]+700, labels="1 km", cex=1.5)

## Adding the legend

box.el=200

ltys=c(3,2,1)

lwds=c(4,2,2)

cols=c(NA,NA,NA,adjustcolor(col="yellow", alpha.f=0.4), adjustcolor(col="cadetblue4", alpha.f=0.4), adjustcolor(col="lightsteelblue2", alpha.f=0.4), "white", "black")

borders=c("green4", "black","blue", "black", "black","black" )

texts=c("MCP", "KDE", "BRB", "Savannah & Beach", "Swamp", "Sea","Forest", "Camp")

for(i in 1:length(texts)){

if(i<=3){

segments(x0=par()[["usr"]][1]+0.5*box.el, x1=par()[["usr"]][1]+2*box.el,

y0=par()[["usr"]][4]-box.el*0.5*i-box.el*(i-1)-box.el/2, y1=par()[["usr"]][4]-box.el*0.5*i-box.el*(i-1)-box.el/2, lwd=lwds[i], lty=ltys[i],col=borders[i])

}else if(i<=7){

rect(xleft=par()[["usr"]][1]+0.5*box.el, xright=par()[["usr"]][1]+1.5*box.el,

ytop= par()[["usr"]][4]-box.el*0.5*i-box.el*(i-1),

ybottom=par()[["usr"]][4]-box.el*0.5*i-box.el*(i-1)-box.el,

col=cols[i], border="black")

}else{

points(x=par()[["usr"]][1]+box.el, y=par()[["usr"]][4]-box.el*0.5*i-box.el*(i-1)-box.el/2, pch=8)

}

text(x=par()[["usr"]][1]+2.5*box.el+50, y=par()[["usr"]][4]-box.el*0.5*i-box.el*(i-1)-box.el/2, labels=texts[i], adj=0, cex=0.8)

}

## Adding the map titletext(x=par()[["usr"]][1]+3000, y=par()[["usr"]][3]+10000, labels="95% Contours 2017", adj=0, cex=1.5)## Saving the map

savePlot(file="C:/Users/laura/Dropbox/Research/LoangoChimpanzeeProject/201909_intercommunity_study/2019refresh/202004_maps/202009_Rekambo_2017_95_percent_contour_comparison.png", type="png", device=dev.cur())

dev.copy2pdf(file="C:/Users/laura/Dropbox/Research/LoangoChimpanzeeProject/201909_intercommunity_study/2019refresh/202004_maps/202009_Rekambo_2017_95_percent_contour_comparison.pdf")####

##Comparing the 50% contours of 2017

#####

ver.kde50.2017=getverticeshr(xkernel2017, percent=50)

ver.brb50.2017=getverticeshr(rek.brb.2017, percent=50)##plot:

par(mar=rep(0.2, 4))

plot(getverticeshr(rek.brb.2017,percent=99,standardize = FALSE), asp=1, xlab="", ylab="",xlim=c(548350, 557873),ylim=c(9765030, 9774195), axes=F, xaxt="n", yaxt="n", bty="n", border=NA)

polygon(MCP2017[["50"]], border="green4", col=NA, lwd=4,lty=3)

for(i in 1:length(ver.kde50.2017@polygons[[1]]@Polygons)){

polygon(ver.kde50.2017@polygons[[1]]@Polygons[[i]]@coords,border="black", lwd=2,lty=2)

}

for(i in 1:length(ver.brb50.2017@polygons[[1]]@Polygons)){

polygon(ver.brb50.2017@polygons[[1]]@Polygons[[i]]@coords, border="blue", col=NA, lty=1, lwd=2)

}

for(i in 1:length(savannah)){

polygon(savannah[[i]], border=NA, col=adjustcolor(col="yellow", alpha.f=0.4))

}

polygon(swamp[[10]], border=NA, col=adjustcolor(col="cadetblue4", alpha.f=0.4))

polygon(sea[[1]], border=NA, col=adjustcolor(col="lightsteelblue2", alpha.f=0.4))

## Adding the location of the Ozouga Research Camp

camp.loc=c(x=553601, y=9765888)

points(x=camp.loc["x"], y=camp.loc["y"], pch=8, cex=1.2, lwd=1)## Adding the scale

arrows(x0=par()[["usr"]][1]+1000, x1=par()[["usr"]][1]+2000, y0=par()[["usr"]][3]+500, y1=par()[["usr"]][3]+500, code=3, angle=90, length=0.05)

text(x=par()[["usr"]][1]+1500, y=par()[["usr"]][3]+700, labels="1 km", cex=1.5)

## Adding the legend

box.el=200

ltys=c(3,2,1)

lwds=c(4,2,2)

cols=c(NA,NA,NA,adjustcolor(col="yellow", alpha.f=0.4), adjustcolor(col="cadetblue4", alpha.f=0.4), adjustcolor(col="lightsteelblue2", alpha.f=0.4), "white", "black")

borders=c("green4", "black","blue", "black", "black","black" )

texts=c("MCP", "KDE", "BRB", "Savannah & Beach", "Swamp", "Sea","Forest", "Camp")

for(i in 1:length(texts)){

if(i<=3){

segments(x0=par()[["usr"]][1]+0.5*box.el, x1=par()[["usr"]][1]+2*box.el,

y0=par()[["usr"]][4]-box.el*0.5*i-box.el*(i-1)-box.el/2, y1=par()[["usr"]][4]-box.el*0.5*i-box.el*(i-1)-box.el/2, lwd=lwds[i], lty=ltys[i],col=borders[i])

}else if(i<=7){

rect(xleft=par()[["usr"]][1]+0.5*box.el, xright=par()[["usr"]][1]+1.5*box.el,

ytop= par()[["usr"]][4]-box.el*0.5*i-box.el*(i-1),

ybottom=par()[["usr"]][4]-box.el*0.5*i-box.el*(i-1)-box.el,

col=cols[i], border="black")

}else{

points(x=par()[["usr"]][1]+box.el, y=par()[["usr"]][4]-box.el*0.5*i-box.el*(i-1)-box.el/2, pch=8)

}

text(x=par()[["usr"]][1]+2.5*box.el+50, y=par()[["usr"]][4]-box.el*0.5*i-box.el*(i-1)-box.el/2, labels=texts[i], adj=0, cex=0.8)

}

## Adding the map title

text(x=par()[["usr"]][1]+3500, y=par()[["usr"]][3]+10000, labels="50% Contours 2017", adj=0, cex=1.5)## Saving the map

savePlot(file="C:/Users/laura/Dropbox/Research/LoangoChimpanzeeProject/201909_intercommunity_study/2019refresh/202004_maps/202009_Rekambo_2017_50_percent_contour_comparison.png", type="png", device=dev.cur())

dev.copy2pdf(file="C:/Users/laura/Dropbox/Research/LoangoChimpanzeeProject/201909_intercommunity_study/2019refresh/202004_maps/202009_Rekambo_2017_50_percent_contour_comparison.pdf")

#########

# 8.6 Areas Under the Curve 2017

#########

#R code from

#Cumming GS, Cornélis D. Quantitative comparison and selection of home range metrics for telemetry

#data. Divers Distrib. 2012; 18(11):1057 - 65. doi: 10.1111/j.1472-4642.2012.00908.x###########

#AUC kernel

##########

#Volumes computation

udvol.k2017<-getvolumeUD(xkernel2017, standardize = FALSE)#KDEhref

udvol.kernel2017<-estUDm2spixdf(udvol.k2017) # conversion of volume contours to format SpatialPixelsDataFrame (necessary for count.cells)

#Counting locations within volume rasters

nlocrast_kernel2017<-count.points(xyALL,udvol.kernel2017)

#AUC calculation

kernel2017data <- udvol.kernel2017@data # vector containing volume contour(=predicted) values

pointdata.kernel2017 <- nlocrast_kernel2017@data$x

str(nlocrast_kernel2017)

pointdata.kernel2017 <- ifelse(pointdata.kernel2017>=1,1,0) # vector containing location (=actual) values

print(AUC_kernel2017 <- colAUC(kernel2017data, pointdata.kernel2017, plotROC=TRUE, alg=c("Wilcoxon","ROC")))

###########

#AUC BRB

##########

#Volumes computation

udvol.brb.2017<-getvolumeUD(rek.brb.2017, standardize = TRUE)#KDEhref

#plot(udvol.brb)#Counting locations within volume rasters

nlocrast_brb2017<-count.points(xy2017,udvol.brb.2017)

brb2017data <- udvol.brb.2017@data$n #vector containing volume contour(=predicted) values

pointdata.brb.2017 <- nlocrast_brb2017@data$x

pointdata.brb.2017 <- ifelse(pointdata.brb.2017>=1,1,0) # vector containing location (=actual) values

print(AUC_brb2017 <- colAUC(brb2017data, pointdata.brb.2017, plotROC=TRUE, alg=c("Wilcoxon","ROC"))) #########

###AUC MCP

#Creating grid 100X100

#Modified from Cumming & Cornelis 2012

#Values for grid

RESO <- 100 # grid resolution (m)

BUFF <- 100 # grid extent (m) (buffer around location extremes)

XMIN <- RESO*(round(((min(xyALL$long)-BUFF)/RESO),0))

YMIN <- RESO*(round(((min(xyALL$lat)-BUFF)/RESO),0))

XMAX <- XMIN+RESO*(round(((max(xyALL$long)+BUFF-XMIN)/RESO),0))

YMAX <- YMIN+RESO*(round(((max(xyALL$lat)+BUFF-YMIN)/RESO),0))

NRW <- ((YMAX-YMIN)/RESO)

NCL <- ((XMAX-XMIN)/RESO)#grid

refgrid<-raster(nrows=NRW, ncols=NCL, xmn=XMIN, xmx=XMAX, ymn=YMIN, ymx=YMAX)

refgrid<-as(refgrid,"SpatialPixels")

#Calculate MCP100(it was inside a funcon originaly)

mcp.rek2017<- mcp(xy2017)

#Trasform into raster (Spatial Pixel Data Frame)

mcp.rek1.2017<-hr.rast(mcp.rek2017, refgrid)

#Counting locations within volume rasters

nlocrast_mcp2017<-count.points(xy2017,mcp.rek1.2017)mcp.rek1.2017@data$a[is.na(mcp.rek1.2017@data$a)] <- 0 #Pixeles not included in MCP were reported as NA, giving problems later on. Reclassified to zero

mcp2017data <- mcp.rek1.2017@data$a # vector containing volume contour(=predicted) values

pointdata.mcp.2017 <- nlocrast_mcp2017@data$x

pointdata.mcp.2017 <- ifelse(pointdata.mcp.2017>=1,1,0) # vector containing location (=actual) values

print(AUC_mcp2017 <- colAUC(mcp2017data, pointdata.mcp.2017, plotROC=TRUE, alg=c("Wilcoxon","ROC"))) #############################################################################

#### 9. Calculating and mapping the home range of the Rekambo community in 2018

#######################################################################################

# 9.1 Preparing the data for analyses

##########Preparing coordinates

utm.coords.2018=WGS.to.UTM(long=tracks.2018$lon, lat=tracks.2018$lat, utm.zone=utm.zone(mean(tracks.2018$lon), mean(tracks.2018$lat)), avoid.negatives=T, return.zone=F)

tracks2018=utm.coords.2018xy2018=SpatialPoints(coords=tracks2018[, c("long", "lat")])#Counting amount of data used in the calculationstracks.2018$yyyymmdd<-as.factor(tracks.2018$yyyymmdd)str(tracks.2018)#No obs=No locations= 221981

#$burst levels=No tracks = 690????????

#$yyyymmdd= No. obs. days=261#Calculating observation time included in the sample

#We will calculate the observation time with the calculation on lag. We will need to remove any with a

#lag >30min, which would be joining different tracks, so time in which there was no chimpanzee observationlags2018<- tracks.2018[tracks.2018$lag <= 30,]

lags2018<- lags2018$lag/60

str(lags2018)

sum(lags2018)#2794.411h approx

#fixing the date to create the ltraj object

date.bbmm.2018 <- as.POSIXct(strptime(tracks.2018$date,format="%d-%m-%Y %H:%M:%S"))

#Creating ltraj object to calculate BRB

ltraj.2018=as.ltraj(xy =tracks2018, date =date.bbmm.2018, id=F)

any(is.na(ltraj.2018))

############## 9.2 MCP of the Rekambo community in 2018 ############# ##Calculating the MCPto.get=c(100, 99, 98, 95, 80, 75, 50)

MCP2018=vector("list", length(to.get))

names(MCP2018)=to.get

for(i in 1:length(to.get)){

xx2018=mcp(xy2018, percent=to.get[i])

MCP2018[[i]]=xx2018@polygons[[1]]@Polygons[[1]]@coords

} #Calculating the MCP areas

MCP.areas.2018=unlist(lapply(MCP2018, areapl))

MCP.areas.2018=MCP.areas.2018/(1000^2)

names(MCP.areas.2018)=to.get

MCP.areas.2018## Plotting the MCP of 2018 over the study site map

par(mar=rep(0.2, 4))

plot(as.matrix(MCP2018[["100"]]), asp=1, xlab="", ylab="",xlim=c(548350, 557873),ylim=c(9765030, 9774195), axes=T, type="n", xaxt="n", yaxt="n", bty="n")

cols=c("mediumaquamarine", "gray0", "gray20", "gray40","gray60", "gray80", "gray95")

for(i in 1:length(to.get)){

polygon(MCP2018[[i]], border=NA, col=cols[i])

}

for(i in 1:length(savannah)){

polygon(savannah[[i]], border=NA, col=adjustcolor(col="yellow", alpha.f=0.4))

}

polygon(swamp[[10]], border=NA, col=adjustcolor(col="cadetblue4", alpha.f=0.4))

polygon(sea[[1]], border=NA, col=adjustcolor(col="lightsteelblue2", alpha.f=0.4))# Adding the location of the Ozouga Research Camp to the map

camp.loc=c(x=553601, y=9765888)

points(x=camp.loc["x"], y=camp.loc["y"], pch=8, cex=1.2, lwd=1)

# Adding the scale

arrows(x0=par()[["usr"]][1]+1000, x1=par()[["usr"]][1]+2000, y0=par()[["usr"]][3]+500, y1=par()[["usr"]][3]+500, code=3, angle=90, length=0.05)

text(x=par()[["usr"]][1]+1500, y=par()[["usr"]][3]+700, labels="1 km",cex=1.5)# Adding the legend

box.el=200

cols=c("mediumaquamarine", "gray0", "gray20", "gray40","gray60", "gray80", "gray95",

adjustcolor(col="yellow", alpha.f=0.4), adjustcolor(col="cadetblue4", alpha.f=0.4), adjustcolor(col="lightsteelblue2", alpha.f=0.4), "white", "black")

borders=rep("black", length(cols))

texts=c(paste(c(100, 99, 98, 95,80, 75, 50), "%", sep=""), "Savannah & Beach", "Swamp", "Sea", "Forest", "Camp")for(i in 1:length(texts)){

if(i<=11){

rect(xleft=par()[["usr"]][1]+0.5*box.el, xright=par()[["usr"]][1]+1.5*box.el,

ytop= par()[["usr"]][4]-box.el*0.5*i-box.el*(i-1),

ybottom=par()[["usr"]][4]-box.el*0.5*i-box.el*(i-1)-box.el,

col=cols[i], border="black")

}else{

points(x=par()[["usr"]][1]+box.el, y=par()[["usr"]][4]-box.el*0.5*i-box.el*(i-1)-box.el/2, pch=8)

}

text(x=par()[["usr"]][1]+1.5*box.el+50, y=par()[["usr"]][4]-box.el*0.5*i-box.el*(i-1)-box.el/2, labels=texts[i], adj=0, cex=0.8)

}

#Adding the title to the map

text(x=par()[["usr"]][1]+4000, y=par()[["usr"]][3]+10000, labels="MCP 2018", adj=0, cex=1.5)#Saving the map

savePlot(file="C:/Users/laura/Dropbox/Research/LoangoChimpanzeeProject/201909_intercommunity_study/2019refresh/202004_maps/202009_MCP_home_range_Rekambo_2018.png", type="png", device=dev.cur())

dev.copy2pdf(file="C:/Users/laura/Dropbox/Research/LoangoChimpanzeeProject/201909_intercommunity_study/2019refresh/202004_maps/202009_MCP_home_range_Rekambo_2018.pdf")############

# 9.3 KDE 2018

############

# Calculating KDE

k.data.2018=SpatialPointsDataFrame(coords=tracks2018, data=data.frame(id=rep("a", nrow(tracks2018))))

xkernel2018=kernelUD(k.data.2018, h="href", grid=100)

xkernel2018[[1]]@h #showing h value

# Preparing to calculate KDE area

kernel.d=rev(c(50, 75, 80, 95,98,99))

kernel.areas.2018=rep(0, length(kernel.d))

names(kernel.areas.2018)=kernel.d

# Plotting the KDE of 2018 over the map of the study site and calculating KDE areas

par(mar=rep(0.2, 4))

plot(getverticeshr(xkernel2018, percent=99), asp=1, xlab="", ylab="",xlim=c(548350, 557873),ylim=c(9765030, 9774195), axes=F, xaxt="n", yaxt="n", bty="n", border=NA)

cols=c("gray0", "gray20", "gray40","gray60", "gray80", "gray95")

for(k in 1:length(kernel.d)){

ver=getverticeshr(xkernel2018, percent=kernel.d[k])

for(i in 1:length(ver@polygons[[1]]@Polygons)){

polygon(ver@polygons[[1]]@Polygons[[i]]@coords, border=NA, col=cols[k])

kernel.areas.2018[k]=kernel.areas.2018[k]+areapl(ver@polygons[[1]]@Polygons[[i]]@coords)/(1000^2)

}

}

for(i in 1:length(savannah)){

polygon(savannah[[i]], border=NA, col=adjustcolor(col="yellow", alpha.f=0.4))

}

polygon(swamp[[10]], border=NA, col=adjustcolor(col="cadetblue4", alpha.f=0.4))

polygon(sea[[1]], border=NA, col=adjustcolor(col="lightsteelblue2", alpha.f=0.4))##Show KDE areas (km2)

kernel.areas.2018# Adding the location of Ozouga Research Camp to the map

camp.loc=c(x=553601, y=9765888)

points(x=camp.loc["x"], y=camp.loc["y"], pch=8, cex=1.2, lwd=1)# Adding the scale

arrows(x0=par()[["usr"]][1]+1000, x1=par()[["usr"]][1]+2000, y0=par()[["usr"]][3]+500, y1=par()[["usr"]][3]+500, code=3, angle=90, length=0.05)

text(x=par()[["usr"]][1]+1500, y=par()[["usr"]][3]+700, labels="1 km", cex=1.5)

# Adding the legend

box.el=200

cols=c("gray20", "gray30", "gray60","gray80", "gray90", "gray95",

adjustcolor(col="yellow", alpha.f=0.4), adjustcolor(col="cadetblue4", alpha.f=0.4), adjustcolor(col="lightsteelblue2", alpha.f=0.4),"white", "black")

borders=rep("black", length(cols))

texts=c(paste(c(99,98, 95,80, 75, 50), "% ", sep=""), "Savannah & Beach", "Swamp", "Sea","Forest", "Camp")for(i in 1:length(texts)){

if(i<=10){

rect(xleft=par()[["usr"]][1]+0.5*box.el, xright=par()[["usr"]][1]+1.5*box.el,

ytop= par()[["usr"]][4]-box.el*0.5*i-box.el*(i-1),

ybottom=par()[["usr"]][4]-box.el*0.5*i-box.el*(i-1)-box.el,

col=cols[i], border="black")

}else{

points(x=par()[["usr"]][1]+box.el, y=par()[["usr"]][4]-box.el*0.5*i-box.el*(i-1)-box.el/2, pch=8)

}

text(x=par()[["usr"]][1]+1.5*box.el+50, y=par()[["usr"]][4]-box.el*0.5*i-box.el*(i-1)-box.el/2, labels=texts[i], adj=0, cex=0.8)

}

# Adding the title to the map

text(x=par()[["usr"]][1]+4500, y=par()[["usr"]][3]+10000, labels="KDE 2018", adj=0, cex=1.5)

# Saving the map

savePlot(file="C:/Users/laura/Dropbox/Research/LoangoChimpanzeeProject/201909_intercommunity_study/2019refresh/202004_maps/202009_KDE_home_ramge_Rekambo_2018.png", type="png", device=dev.cur())

dev.copy2pdf(file="C:/Users/laura/Dropbox/Research/LoangoChimpanzeeProject/201909_intercommunity_study/2019refresh/202004_maps/202009_KDE_home_ramge_Rekambo_2018.pdf")###########

# 9.4 BRB 2018

############ Calculating the BRB

rek.brb.2018 <- BRB(ltraj.2018, D = 1.6, Tmax = 120*60, Lmin = 50, hmin=100,type="UD", grid = 100)# Preparing to calculate the areas of the BRB

brb.cont=rev(c(50, 75, 80, 95,98,99))

brb.areas.2018=rep(0, length(brb.cont))

names(brb.areas.2018)=brb.cont#Plotting the BRB of 2018 over the map of the study site and calculating the BRB of 2018

par(mar=rep(0.2, 4))

plot(getverticeshr(rek.brb.2018,percent=99,standardize = FALSE), asp=1, xlab="", ylab="",xlim=c(548350, 557873),ylim=c(9765030, 9774195), axes=F, xaxt="n", yaxt="n", bty="n", border=NA)

cols=c("gray0", "gray20", "gray40","gray60", "gray80", "gray95")

for(k in 1:length(brb.cont)){

ver.brb.2018=getverticeshr(rek.brb.2018, percent=brb.cont[k])

for(i in 1:length(ver.brb.2018@polygons[[1]]@Polygons)){

polygon(ver.brb.2018@polygons[[1]]@Polygons[[i]]@coords, border=NA, col=cols[k])

brb.areas.2018[k]=brb.areas.2018[k]+areapl(ver.brb.2018@polygons[[1]]@Polygons[[i]]@coords)/(1000^2)

}

}

for(i in 1:length(savannah)){

polygon(savannah[[i]], border=NA, col=adjustcolor(col="yellow", alpha.f=0.4))

}

polygon(swamp[[10]], border=NA, col=adjustcolor(col="cadetblue4", alpha.f=0.4))

polygon(sea[[1]], border=NA, col=adjustcolor(col="lightsteelblue2", alpha.f=0.4))

##Showing the value of the areas of BRB 2018

brb.areas.2018# Adding the location of Ozouga Research Camp to the map

camp.loc=c(x=553601, y=9765888)

points(x=camp.loc["x"], y=camp.loc["y"], pch=8, cex=1.2, lwd=1)# Adding the scale

arrows(x0=par()[["usr"]][1]+1000, x1=par()[["usr"]][1]+2000, y0=par()[["usr"]][3]+500, y1=par()[["usr"]][3]+500, code=3, angle=90, length=0.05)

text(x=par()[["usr"]][1]+1500, y=par()[["usr"]][3]+700, labels="1 km", cex=1.5)

# Adding the legend

box.el=200

cols=c("gray20", "gray30", "gray60","gray80", "gray90", "gray95",

adjustcolor(col="yellow", alpha.f=0.4), adjustcolor(col="cadetblue4", alpha.f=0.4), adjustcolor(col="lightsteelblue2", alpha.f=0.4),"white", "black")

borders=rep("black", length(cols))

texts=c(paste(c(99,98, 95,80, 75, 50), "% ", sep=""), "Savannah & Beach", "Swamp", "Sea","Forest", "Camp")for(i in 1:length(texts)){

if(i<=10){

rect(xleft=par()[["usr"]][1]+0.5*box.el, xright=par()[["usr"]][1]+1.5*box.el,

ytop= par()[["usr"]][4]-box.el*0.5*i-box.el*(i-1),

ybottom=par()[["usr"]][4]-box.el*0.5*i-box.el*(i-1)-box.el,

col=cols[i], border="black")

}else{

points(x=par()[["usr"]][1]+box.el, y=par()[["usr"]][4]-box.el*0.5*i-box.el*(i-1)-box.el/2, pch=8)

}

text(x=par()[["usr"]][1]+1.5*box.el+50, y=par()[["usr"]][4]-box.el*0.5*i-box.el*(i-1)-box.el/2, labels=texts[i], adj=0, cex=0.8)

}

# Adding the title to the map

text(x=par()[["usr"]][1]+4500, y=par()[["usr"]][3]+10000, labels="BRB 2018", adj=0, cex=1.5)# Saving the map

savePlot(file="C:/Users/laura/Dropbox/Research/LoangoChimpanzeeProject/201909_intercommunity_study/2019refresh/202004_maps/202009_BRB_Rekambo_2018.png", type="png", device=dev.cur())

dev.copy2pdf(file="C:/Users/laura/Dropbox/Research/LoangoChimpanzeeProject/201909_intercommunity_study/2019refresh/202004_maps/202009_BRB_Rekambo_2018.pdf")############### 9.5 Comparing the 95% and 50% contours of 2018 for the three estimators ######################

### 9.5.1 Comparing 95% contours of 2018

########

ver.kde95.2018=getverticeshr(xkernel2018, percent=95)

ver.brb.2018=getverticeshr(rek.brb.2018, percent=95)## Plotting

par(mar=rep(0.2, 4))

plot(getverticeshr(rek.brb.2018,percent=99,standardize = FALSE), asp=1, xlab="", ylab="",xlim=c(548350, 557873),ylim=c(9765030, 9774195), axes=F, xaxt="n", yaxt="n", bty="n", border=NA)

polygon(MCP2018[["95"]], border="green4", col=NA, lty=3, lwd=4)

for(i in 1:length(ver.kde95.2018@polygons[[1]]@Polygons)){

polygon(ver.kde95.2018@polygons[[1]]@Polygons[[i]]@coords, border="black", lwd=2,lty=2)

}

for(i in 1:length(ver.brb.2018@polygons[[1]]@Polygons)){

polygon(ver.brb.2018@polygons[[1]]@Polygons[[i]]@coords, border="blue", col=NA, lty=1, lwd=2)

}

for(i in 1:length(savannah)){

polygon(savannah[[i]], border=NA, col=adjustcolor(col="yellow", alpha.f=0.4))

}

polygon(swamp[[10]], border=NA, col=adjustcolor(col="cadetblue4", alpha.f=0.4))

polygon(sea[[1]], border=NA, col=adjustcolor(col="lightsteelblue2", alpha.f=0.4))

# Adding the location of the Ozouga Research Camp:

camp.loc=c(x=553601, y=9765888)

points(x=camp.loc["x"], y=camp.loc["y"], pch=8, cex=1.2, lwd=1)# Adding the scale:

arrows(x0=par()[["usr"]][1]+1000, x1=par()[["usr"]][1]+2000, y0=par()[["usr"]][3]+500, y1=par()[["usr"]][3]+500, code=3, angle=90, length=0.05)

text(x=par()[["usr"]][1]+1500, y=par()[["usr"]][3]+700, labels="1 km", cex=1.5)

# Adding the legend:

box.el=200

ltys=c(3,2,1)

lwds=c(4,2,2)

cols=c(NA,NA,NA,adjustcolor(col="yellow", alpha.f=0.4), adjustcolor(col="cadetblue4", alpha.f=0.4), adjustcolor(col="lightsteelblue2", alpha.f=0.4), "white", "black")

borders=c("green4", "black","blue", "black", "black","black" )

texts=c("MCP", "KDE", "BRB", "Savannah & Beach", "Swamp", "Sea","Forest", "Camp")

for(i in 1:length(texts)){

if(i<=3){

segments(x0=par()[["usr"]][1]+0.5*box.el, x1=par()[["usr"]][1]+2*box.el,

y0=par()[["usr"]][4]-box.el*0.5*i-box.el*(i-1)-box.el/2, y1=par()[["usr"]][4]-box.el*0.5*i-box.el*(i-1)-box.el/2, lwd=lwds[i], lty=ltys[i],col=borders[i])

}else if(i<=7){

rect(xleft=par()[["usr"]][1]+0.5*box.el, xright=par()[["usr"]][1]+1.5*box.el,

ytop= par()[["usr"]][4]-box.el*0.5*i-box.el*(i-1),

ybottom=par()[["usr"]][4]-box.el*0.5*i-box.el*(i-1)-box.el,

col=cols[i], border="black")

}else{

points(x=par()[["usr"]][1]+box.el, y=par()[["usr"]][4]-box.el*0.5*i-box.el*(i-1)-box.el/2, pch=8)

}

text(x=par()[["usr"]][1]+2.5*box.el+50, y=par()[["usr"]][4]-box.el*0.5*i-box.el*(i-1)-box.el/2, labels=texts[i], adj=0, cex=0.8)

}

## Adding the map title

text(x=par()[["usr"]][1]+3000, y=par()[["usr"]][3]+10000, labels="95% Contours 2018", adj=0, cex=1.5)# Saving the map

savePlot(file="C:/Users/laura/Dropbox/Research/LoangoChimpanzeeProject/201909_intercommunity_study/2019refresh/202004_maps/202009_Rekambo_2018_95_percent_contour_comparison.png", type="png", device=dev.cur())

dev.copy2pdf(file="C:/Users/laura/Dropbox/Research/LoangoChimpanzeeProject/201909_intercommunity_study/2019refresh/202004_maps/202009_Rekambo_2018_95_percent_contour_comparison.pdf")

########

### 9.5.2 Comparing 50% contours of 2018

########

ver.kde50.2018=getverticeshr(xkernel2018, percent=50)

ver.brb50.2018=getverticeshr(rek.brb.2018, percent=50)# Plotting

par(mar=rep(0.2, 4))

plot(getverticeshr(rek.brb.2018,percent=99,standardize = FALSE), asp=1, xlab="", ylab="",xlim=c(548350, 557873),ylim=c(9765030, 9774195), axes=F, xaxt="n", yaxt="n", bty="n", border=NA)

polygon(MCP2018[["50"]], border="green4", col=NA, lty=3, lwd=3)

for(i in 1:length(ver.kde50.2018@polygons[[1]]@Polygons)){

polygon(ver.kde50.2018@polygons[[1]]@Polygons[[i]]@coords, border="black", lwd=2,lty=2)

}

for(i in 1:length(ver.brb50.2018@polygons[[1]]@Polygons)){

polygon(ver.brb50.2018@polygons[[1]]@Polygons[[i]]@coords, border="blue", col=NA, lty=1, lwd=2)

}

for(i in 1:length(savannah)){

polygon(savannah[[i]], border=NA, col=adjustcolor(col="yellow", alpha.f=0.4))

}

polygon(swamp[[10]], border=NA, col=adjustcolor(col="cadetblue4", alpha.f=0.4))

polygon(sea[[1]], border=NA, col=adjustcolor(col="lightsteelblue2", alpha.f=0.4))

# Adding the location of the Ozouga Research Camp:

camp.loc=c(x=553601, y=9765888)

points(x=camp.loc["x"], y=camp.loc["y"], pch=8, cex=1.2, lwd=1)

# Adding the scale:

arrows(x0=par()[["usr"]][1]+1000, x1=par()[["usr"]][1]+2000, y0=par()[["usr"]][3]+500, y1=par()[["usr"]][3]+500, code=3, angle=90, length=0.05)

text(x=par()[["usr"]][1]+1500, y=par()[["usr"]][3]+700, labels="1 km", cex=1.5)# Adding the legend:

box.el=200

ltys=c(3,2,1)

lwds=c(4,2,2)

cols=c(NA,NA,NA,adjustcolor(col="yellow", alpha.f=0.4), adjustcolor(col="cadetblue4", alpha.f=0.4), adjustcolor(col="lightsteelblue2", alpha.f=0.4), "white", "black")

borders=c("green4", "black","blue", "black", "black","black" )

texts=c("MCP", "KDE", "BRB", "Savannah & Beach", "Swamp", "Sea","Forest", "Camp")

for(i in 1:length(texts)){

if(i<=3){

segments(x0=par()[["usr"]][1]+0.5*box.el, x1=par()[["usr"]][1]+2*box.el,

y0=par()[["usr"]][4]-box.el*0.5*i-box.el*(i-1)-box.el/2, y1=par()[["usr"]][4]-box.el*0.5*i-box.el*(i-1)-box.el/2, lwd=lwds[i], lty=ltys[i],col=borders[i])

}else if(i<=7){

rect(xleft=par()[["usr"]][1]+0.5*box.el, xright=par()[["usr"]][1]+1.5*box.el,

ytop= par()[["usr"]][4]-box.el*0.5*i-box.el*(i-1),

ybottom=par()[["usr"]][4]-box.el*0.5*i-box.el*(i-1)-box.el,

col=cols[i], border="black")

}else{

points(x=par()[["usr"]][1]+box.el, y=par()[["usr"]][4]-box.el*0.5*i-box.el*(i-1)-box.el/2, pch=8)

}

text(x=par()[["usr"]][1]+2.5*box.el+50, y=par()[["usr"]][4]-box.el*0.5*i-box.el*(i-1)-box.el/2, labels=texts[i], adj=0, cex=0.8)

}

## Adding the map title

text(x=par()[["usr"]][1]+3500, y=par()[["usr"]][3]+10000, labels="50% Contours 2018", adj=0, cex=1.5)

# Saving the map

savePlot(file="C:/Users/laura/Dropbox/Research/LoangoChimpanzeeProject/201909_intercommunity_study/2019refresh/202004_maps/202009_Rekambo_2018_50_percent_contour_comparison.png", type="png", device=dev.cur())

dev.copy2pdf(file="C:/Users/laura/Dropbox/Research/LoangoChimpanzeeProject/201909_intercommunity_study/2019refresh/202004_maps/202009_Rekambo_2018_50_percent_contour_comparison.pdf")

##############

# 9.6 Area Under the Curve 2018

##############

#R code from

#Cumming GS, Cornélis D. Quantitative comparison and selection of home range metrics for telemetry

#data. Divers Distrib. 2012; 18(11):1057 - 65. doi: 10.1111/j.1472-4642.2012.00908.x

###########

#AUC KDE

##########

#Volumes computation

udvol.k2018<-getvolumeUD(xkernel2018, standardize = FALSE)#KDEhref

udvol.kernel2018<-estUDm2spixdf(udvol.k2018) # conversion of volume contours to format SpatialPixelsDataFrame (necessary for count.cells)#Counting locations within volume rasters

nlocrast_kernel2018<-count.points(xyALL,udvol.kernel2018)

kernel2018data <- udvol.kernel2018@data # vector containing volume contour(=predicted) values

pointdata.kernel2018 <- nlocrast_kernel2018@data$x

pointdata.kernel2018 <- ifelse(pointdata.kernel2018>=1,1,0) # vector containing location (=actual) values

#Calculating AUC

par(mar=rep(6,4))

print(AUC_kernel2018 <- colAUC(kernel2018data, pointdata.kernel2018, plotROC=TRUE, alg=c("Wilcoxon","ROC")))

###########

#AUC BRB

##########

#Volumes computation

udvol.brb.2018<-getvolumeUD(rek.brb.2018, standardize = TRUE)#KDEhref

#plot(udvol.brb)

#Counting locations within volume rasters

nlocrast_brb2018<-count.points(xy2018,udvol.brb.2018)

brb2018data <- udvol.brb.2018@data$n #vector containing volume contour(=predicted) values

pointdata.brb.2018 <- nlocrast_brb2018@data$x

pointdata.brb.2018 <- ifelse(pointdata.brb.2018>=1,1,0) # vector containing location (=actual) values

#Calculating AUC

par(mar=rep(6,4))

print(AUC_brb2018 <- colAUC(brb2018data, pointdata.brb.2018, plotROC=TRUE, alg=c("Wilcoxon","ROC")))

#########

###AUC MCP

#########

#Calculate MCP100(it was inside a function originally)

mcp.rek2018<- mcp(xy2018)

#Trasform into raster (Spatial Pixel Data Frame)

mcp.rek1.2018<-hr.rast(mcp.rek2018, refgrid)

#Counting locations within volume rasters

nlocrast_mcp2018<-count.points(xy2018,mcp.rek1.2018)

mcp.rek1.2018@data$a[is.na(mcp.rek1.2018@data$a)] <- 0 #Pixeles not included in MCP were reported as NA, giving problems later on. Reclassified to zero

mcp2018data <- mcp.rek1.2018@data$a # vector containing volume contour(=predicted) values

pointdata.mcp.2018 <- nlocrast_mcp2018@data$x

pointdata.mcp.2018 <- ifelse(pointdata.mcp.2018>=1,1,0) # vector containing location (=actual) values

#Calculating AUC

par(mar=rep(6,4))

print(AUC_mcp2018 <- colAUC(mcp2018data, pointdata.mcp.2018, plotROC=TRUE, alg=c("Wilcoxon","ROC")))

#####################################################################

#### 10. Effects of relocation subsampling in MCP and KDE ##########

#####################################################################

########################

# 10.1 Preparing the data for the sub-sampling

########################

###20181030 needs to be dropped because it only has 2 data points, so it cannot be subsampled with over 2 points per day

alltracks = alltracks[!alltracks$yyyymmdd==20181030 ,]

alltracks$yyyymmdd<-as.factor(alltracks$yyyymmdd)

########################

# 10.2 Calculating MCP and KDE with one sample per day (x10 trials)

########################

####

##Trial 1

####

set.seed(1)#Seeds were set so the random numbers chosen were always the same one each time R was run, so the result could be replicable

tracks_1perday_trial1<-alltracks %>% group_by(yyyymmdd) %>% sample_n(size = 1)

##Preparing the coordinates for the calculations

utm_1perday_trial1=WGS.to.UTM(long=tracks_1perday_trial1$lon, lat=tracks_1perday_trial1$lat, utm.zone=utm.zone(mean(tracks_1perday_trial1$lon), mean(tracks_1perday_trial1$lat)), avoid.negatives=T, return.zone=F)

xy_1perday_trial1=SpatialPoints(coords=utm_1perday_trial1[, c("long", "lat")])

to.get=c(100, 99, 98, 95, 80, 75, 50)

MCP_1perday_trial1=vector("list", length(to.get))

names(MCP_1perday_trial1)=to.get

for(i in 1:length(to.get)){

xx_1perday_trial1=mcp(xy_1perday_trial1, percent=to.get[i])

MCP_1perday_trial1[[i]]=xx_1perday_trial1@polygons[[1]]@Polygons[[1]]@coords

}

MCP.areas_1perday_trial1=unlist(lapply(MCP_1perday_trial1, areapl))

MCP.areas_1perday_trial1=MCP.areas_1perday_trial1/(1000^2)

names(MCP.areas_1perday_trial1)=to.get

MCP.areas_1perday_trial1

k.data_1perday_trial1=SpatialPointsDataFrame(coords=utm_1perday_trial1, data=data.frame(id=rep("a", nrow(utm_1perday_trial1))))

xkernel_1perday_trial1=kernelUD(k.data_1perday_trial1, h="href", grid=100, extent=1)

##plot:

par(mar=rep(0.2, 4))

plot(as.matrix(MCP_1perday_trial1[["100"]]), asp=1, xlab="", ylab="",xlim=c(548350, 557873),ylim=c(9765030, 9774195), axes=T, type="n", xaxt="n", yaxt="n", bty="n")

cols=c("mediumaquamarine", "gray20", "gray30", "gray60","gray80", "gray90", "gray95")

for(i in 1:length(to.get)){

polygon(MCP_1perday_trial1[[i]], border=NA, col=cols[i])

}

for(i in 1:length(savannah)){

polygon(savannah[[i]], border=NA, col=adjustcolor(col="yellow", alpha.f=0.4))

}

polygon(swamp[[10]], border=NA, col=grey(level=0.4, alpha=0.4))

polygon(sea[[1]], border=NA, col="lightsteelblue2")

#add kernel:

kernel.d_1perday_trial1=rev(c(50, 75, 80, 95,98,99))

kernel.areas_1perday_trial1=rep(0, length(kernel.d_1perday_trial1))

names(kernel.areas_1perday_trial1)=kernel.d_1perday_trial1

ltys=c(1, 2,3,4,5,6)

lwds=c(1, 1,2,2,3,3)

for(k in 1:length(kernel.d_1perday_trial1)){

ver=getverticeshr(xkernel_1perday_trial1, percent=kernel.d_1perday_trial1[k])

for(i in 1:length(ver@polygons[[1]]@Polygons)){

polygon(ver@polygons[[1]]@Polygons[[i]]@coords, border=grey(level=0.25), col=NA, lty=ltys[k],lwd=lwds[k])

kernel.areas_1perday_trial1[k]=kernel.areas_1perday_trial1[k]+areapl(ver@polygons[[1]]@Polygons[[i]]@coords)/(1000^2)

}

}

kernel.areas_1perday_trial1

####

##Trial 2

####

set.seed(2)

tracks_1perday_trial2<-alltracks %>% group_by(yyyymmdd) %>% sample_n(size = 1)

##Preparing the coordinates for the calculations

utm_1perday_trial2=WGS.to.UTM(long=tracks_1perday_trial2$lon, lat=tracks_1perday_trial2$lat, utm.zone=utm.zone(mean(tracks_1perday_trial2$lon), mean(tracks_1perday_trial2$lat)), avoid.negatives=T, return.zone=F)

xy_1perday_trial2=SpatialPoints(coords=utm_1perday_trial2[, c("long", "lat")])

to.get=c(100, 99, 98, 95, 80, 75, 50)

MCP_1perday_trial2=vector("list", length(to.get))

names(MCP_1perday_trial2)=to.get

for(i in 1:length(to.get)){

xx_1perday_trial2=mcp(xy_1perday_trial2, percent=to.get[i])

MCP_1perday_trial2[[i]]=xx_1perday_trial2@polygons[[1]]@Polygons[[1]]@coords

}

MCP.areas_1perday_trial2=unlist(lapply(MCP_1perday_trial2, areapl))

MCP.areas_1perday_trial2=MCP.areas_1perday_trial2/(1000^2)

names(MCP.areas_1perday_trial2)=to.get

MCP.areas_1perday_trial2

k.data_1perday_trial2=SpatialPointsDataFrame(coords=utm_1perday_trial2, data=data.frame(id=rep("a", nrow(utm_1perday_trial2))))

xkernel_1perday_trial2=kernelUD(k.data_1perday_trial2, h="href", grid=100, extent=1)

##plot:

par(mar=rep(0.2, 4))

plot(as.matrix(MCP_1perday_trial2[["100"]]), asp=1, xlab="", ylab="",xlim=c(548350, 557873),ylim=c(9765030, 9774195), axes=T, type="n", xaxt="n", yaxt="n", bty="n")

cols=c("mediumaquamarine", "gray20", "gray30", "gray60","gray80", "gray90", "gray95")

for(i in 1:length(to.get)){

polygon(MCP_1perday_trial2[[i]], border=NA, col=cols[i])

}

for(i in 1:length(savannah)){

polygon(savannah[[i]], border=NA, col=adjustcolor(col="yellow", alpha.f=0.4))

}

polygon(swamp[[10]], border=NA, col=grey(level=0.4, alpha=0.4))

polygon(sea[[1]], border=NA, col="lightsteelblue2")

#add kernel:

kernel.d_1perday_trial2=rev(c(50, 75, 80, 95,98,99))

kernel.areas_1perday_trial2=rep(0, length(kernel.d_1perday_trial2))

names(kernel.areas_1perday_trial2)=kernel.d_1perday_trial2

ltys=c(1, 2,3,4,5,6)

lwds=c(1, 1,2,2,3,3)

for(k in 1:length(kernel.d_1perday_trial2)){

ver=getverticeshr(xkernel_1perday_trial2, percent=kernel.d_1perday_trial2[k])

for(i in 1:length(ver@polygons[[1]]@Polygons)){

polygon(ver@polygons[[1]]@Polygons[[i]]@coords, border=grey(level=0.25), col=NA, lty=ltys[k],lwd=lwds[k])

kernel.areas_1perday_trial2[k]=kernel.areas_1perday_trial2[k]+areapl(ver@polygons[[1]]@Polygons[[i]]@coords)/(1000^2)

}

}

kernel.areas_1perday_trial2

####

##Trial 3

####

set.seed(3)

tracks_1perday_trial3<-alltracks %>% group_by(yyyymmdd) %>% sample_n(size = 1)

##Preparing the coordinates for the calculations

utm_1perday_trial3=WGS.to.UTM(long=tracks_1perday_trial3$lon, lat=tracks_1perday_trial3$lat, utm.zone=utm.zone(mean(tracks_1perday_trial3$lon), mean(tracks_1perday_trial3$lat)), avoid.negatives=T, return.zone=F)

xy_1perday_trial3=SpatialPoints(coords=utm_1perday_trial3[, c("long", "lat")])

to.get=c(100, 99, 98, 95, 80, 75, 50)

MCP_1perday_trial3=vector("list", length(to.get))

names(MCP_1perday_trial3)=to.get

for(i in 1:length(to.get)){

xx_1perday_trial3=mcp(xy_1perday_trial3, percent=to.get[i])

MCP_1perday_trial3[[i]]=xx_1perday_trial3@polygons[[1]]@Polygons[[1]]@coords

}

MCP.areas_1perday_trial3=unlist(lapply(MCP_1perday_trial3, areapl))

MCP.areas_1perday_trial3=MCP.areas_1perday_trial3/(1000^2)

names(MCP.areas_1perday_trial3)=to.get

MCP.areas_1perday_trial3

k.data_1perday_trial3=SpatialPointsDataFrame(coords=utm_1perday_trial3, data=data.frame(id=rep("a", nrow(utm_1perday_trial3))))

xkernel_1perday_trial3=kernelUD(k.data_1perday_trial3, h="href", grid=100, extent=1)

##plot:

par(mar=rep(0.2, 4))

plot(as.matrix(MCP_1perday_trial3[["100"]]), asp=1, xlab="", ylab="",xlim=c(548350, 557873),ylim=c(9765030, 9774195), axes=T, type="n", xaxt="n", yaxt="n", bty="n")

cols=c("mediumaquamarine", "gray20", "gray30", "gray60","gray80", "gray90", "gray95")

for(i in 1:length(to.get)){

polygon(MCP_1perday_trial3[[i]], border=NA, col=cols[i])

}

for(i in 1:length(savannah)){

polygon(savannah[[i]], border=NA, col=adjustcolor(col="yellow", alpha.f=0.4))

}

polygon(swamp[[10]], border=NA, col=grey(level=0.4, alpha=0.4))

polygon(sea[[1]], border=NA, col="lightsteelblue2")

#add kernel:

kernel.d_1perday_trial3=rev(c(50, 75, 80, 95,98,99))

kernel.areas_1perday_trial3=rep(0, length(kernel.d_1perday_trial3))

names(kernel.areas_1perday_trial3)=kernel.d_1perday_trial3

ltys=c(1, 2,3,4,5,6)

lwds=c(1, 1,2,2,3,3)

for(k in 1:length(kernel.d_1perday_trial3)){

ver=getverticeshr(xkernel_1perday_trial3, percent=kernel.d_1perday_trial3[k])

for(i in 1:length(ver@polygons[[1]]@Polygons)){

polygon(ver@polygons[[1]]@Polygons[[i]]@coords, border=grey(level=0.25), col=NA, lty=ltys[k],lwd=lwds[k])

kernel.areas_1perday_trial3[k]=kernel.areas_1perday_trial3[k]+areapl(ver@polygons[[1]]@Polygons[[i]]@coords)/(1000^2)

}

}

kernel.areas_1perday_trial3

####

##Trial 4

####

set.seed(4)

tracks_1perday_trial4<-alltracks %>% group_by(yyyymmdd) %>% sample_n(size = 1)##Preparing the coordinates for the calculationsutm_1perday_trial4=WGS.to.UTM(long=tracks_1perday_trial4$lon, lat=tracks_1perday_trial4$lat, utm.zone=utm.zone(mean(tracks_1perday_trial4$lon), mean(tracks_1perday_trial4$lat)), avoid.negatives=T, return.zone=F)

xy_1perday_trial4=SpatialPoints(coords=utm_1perday_trial4[, c("long", "lat")])

to.get=c(100, 99, 98, 95, 80, 75, 50)

MCP_1perday_trial4=vector("list", length(to.get))

names(MCP_1perday_trial4)=to.get

for(i in 1:length(to.get)){

xx_1perday_trial4=mcp(xy_1perday_trial4, percent=to.get[i])

MCP_1perday_trial4[[i]]=xx_1perday_trial4@polygons[[1]]@Polygons[[1]]@coords

}

MCP.areas_1perday_trial4=unlist(lapply(MCP_1perday_trial4, areapl))

MCP.areas_1perday_trial4=MCP.areas_1perday_trial4/(1000^2)

names(MCP.areas_1perday_trial4)=to.get

MCP.areas_1perday_trial4

k.data_1perday_trial4=SpatialPointsDataFrame(coords=utm_1perday_trial4, data=data.frame(id=rep("a", nrow(utm_1perday_trial4))))

xkernel_1perday_trial4=kernelUD(k.data_1perday_trial4, h="href", grid=100, extent=1)

##plot:

par(mar=rep(0.2, 4))

plot(as.matrix(MCP_1perday_trial4[["100"]]), asp=1, xlab="", ylab="",xlim=c(548350, 557873),ylim=c(9765030, 9774195), axes=T, type="n", xaxt="n", yaxt="n", bty="n")

cols=c("mediumaquamarine", "gray20", "gray30", "gray60","gray80", "gray90", "gray95")

for(i in 1:length(to.get)){

polygon(MCP_1perday_trial4[[i]], border=NA, col=cols[i])

}

for(i in 1:length(savannah)){

polygon(savannah[[i]], border=NA, col=adjustcolor(col="yellow", alpha.f=0.4))

}

polygon(swamp[[10]], border=NA, col=grey(level=0.4, alpha=0.4))

polygon(sea[[1]], border=NA, col="lightsteelblue2")

#add kernel:

kernel.d_1perday_trial4=rev(c(50, 75, 80, 95,98,99))

kernel.areas_1perday_trial4=rep(0, length(kernel.d_1perday_trial4))

names(kernel.areas_1perday_trial4)=kernel.d_1perday_trial4

ltys=c(1, 2,3,4,5,6)

lwds=c(1, 1,2,2,3,3)

for(k in 1:length(kernel.d_1perday_trial4)){

ver=getverticeshr(xkernel_1perday_trial4, percent=kernel.d_1perday_trial4[k])

for(i in 1:length(ver@polygons[[1]]@Polygons)){

polygon(ver@polygons[[1]]@Polygons[[i]]@coords, border=grey(level=0.25), col=NA, lty=ltys[k],lwd=lwds[k])

kernel.areas_1perday_trial4[k]=kernel.areas_1perday_trial4[k]+areapl(ver@polygons[[1]]@Polygons[[i]]@coords)/(1000^2)

}

}

kernel.areas_1perday_trial4

####

##Trial 5

####

set.seed(5)

tracks_1perday_trial5<-alltracks %>% group_by(yyyymmdd) %>% sample_n(size = 1)

##Preparing the coordinates for the calculations

utm_1perday_trial5=WGS.to.UTM(long=tracks_1perday_trial5$lon, lat=tracks_1perday_trial5$lat, utm.zone=utm.zone(mean(tracks_1perday_trial5$lon), mean(tracks_1perday_trial5$lat)), avoid.negatives=T, return.zone=F)

xy_1perday_trial5=SpatialPoints(coords=utm_1perday_trial5[, c("long", "lat")])

to.get=c(100, 99, 98, 95, 80, 75, 50)

MCP_1perday_trial5=vector("list", length(to.get))

names(MCP_1perday_trial5)=to.get

for(i in 1:length(to.get)){

xx_1perday_trial5=mcp(xy_1perday_trial5, percent=to.get[i])

MCP_1perday_trial5[[i]]=xx_1perday_trial5@polygons[[1]]@Polygons[[1]]@coords

}

MCP.areas_1perday_trial5=unlist(lapply(MCP_1perday_trial5, areapl))

MCP.areas_1perday_trial5=MCP.areas_1perday_trial5/(1000^2)

names(MCP.areas_1perday_trial5)=to.get

MCP.areas_1perday_trial5

k.data_1perday_trial5=SpatialPointsDataFrame(coords=utm_1perday_trial5, data=data.frame(id=rep("a", nrow(utm_1perday_trial5))))

xkernel_1perday_trial5=kernelUD(k.data_1perday_trial5, h="href", grid=100, extent=1)

##plot:

par(mar=rep(0.2, 4))

plot(as.matrix(MCP_1perday_trial5[["100"]]), asp=1, xlab="", ylab="",xlim=c(548350, 557873),ylim=c(9765030, 9774195), axes=T, type="n", xaxt="n", yaxt="n", bty="n")

cols=c("mediumaquamarine", "gray20", "gray30", "gray60","gray80", "gray90", "gray95")

for(i in 1:length(to.get)){

polygon(MCP_1perday_trial5[[i]], border=NA, col=cols[i])

}

for(i in 1:length(savannah)){

polygon(savannah[[i]], border=NA, col=adjustcolor(col="yellow", alpha.f=0.4))

}

polygon(swamp[[10]], border=NA, col=grey(level=0.4, alpha=0.4))

polygon(sea[[1]], border=NA, col="lightsteelblue2")

#add kernel:

kernel.d_1perday_trial5=rev(c(50, 75, 80, 95,98,99))

kernel.areas_1perday_trial5=rep(0, length(kernel.d_1perday_trial5))

names(kernel.areas_1perday_trial5)=kernel.d_1perday_trial5

ltys=c(1, 2,3,4,5,6)

lwds=c(1, 1,2,2,3,3)

for(k in 1:length(kernel.d_1perday_trial5)){

ver=getverticeshr(xkernel_1perday_trial5, percent=kernel.d_1perday_trial5[k])

for(i in 1:length(ver@polygons[[1]]@Polygons)){

polygon(ver@polygons[[1]]@Polygons[[i]]@coords, border=grey(level=0.25), col=NA, lty=ltys[k],lwd=lwds[k])

kernel.areas_1perday_trial5[k]=kernel.areas_1perday_trial5[k]+areapl(ver@polygons[[1]]@Polygons[[i]]@coords)/(1000^2)

}

}

kernel.areas_1perday_trial5

####

##Trial 6

####

set.seed(6)

tracks_1perday_trial6<-alltracks %>% group_by(yyyymmdd) %>% sample_n(size = 1)

##Preparing the coordinates for the calculations

utm_1perday_trial6=WGS.to.UTM(long=tracks_1perday_trial6$lon, lat=tracks_1perday_trial6$lat, utm.zone=utm.zone(mean(tracks_1perday_trial6$lon), mean(tracks_1perday_trial6$lat)), avoid.negatives=T, return.zone=F)

xy_1perday_trial6=SpatialPoints(coords=utm_1perday_trial6[, c("long", "lat")])

to.get=c(100, 99, 98, 95, 80, 75, 50)

MCP_1perday_trial6=vector("list", length(to.get))

names(MCP_1perday_trial6)=to.get

for(i in 1:length(to.get)){

xx_1perday_trial6=mcp(xy_1perday_trial6, percent=to.get[i])

MCP_1perday_trial6[[i]]=xx_1perday_trial6@polygons[[1]]@Polygons[[1]]@coords

}

MCP.areas_1perday_trial6=unlist(lapply(MCP_1perday_trial6, areapl))

MCP.areas_1perday_trial6=MCP.areas_1perday_trial6/(1000^2)

names(MCP.areas_1perday_trial6)=to.get

MCP.areas_1perday_trial6k.data_1perday_trial6=SpatialPointsDataFrame(coords=utm_1perday_trial6, data=data.frame(id=rep("a", nrow(utm_1perday_trial6))))

xkernel_1perday_trial6=kernelUD(k.data_1perday_trial6, h="href", grid=100, extent=1)

##plot:

par(mar=rep(0.2, 4))

plot(as.matrix(MCP_1perday_trial6[["100"]]), asp=1, xlab="", ylab="",xlim=c(548350, 557873),ylim=c(9765030, 9774195), axes=T, type="n", xaxt="n", yaxt="n", bty="n")

cols=c("mediumaquamarine", "gray20", "gray30", "gray60","gray80", "gray90", "gray95")

for(i in 1:length(to.get)){

polygon(MCP_1perday_trial6[[i]], border=NA, col=cols[i])

}

for(i in 1:length(savannah)){

polygon(savannah[[i]], border=NA, col=adjustcolor(col="yellow", alpha.f=0.4))

}

polygon(swamp[[10]], border=NA, col=grey(level=0.4, alpha=0.4))

polygon(sea[[1]], border=NA, col="lightsteelblue2")

#add kernel:

kernel.d_1perday_trial6=rev(c(50, 75, 80, 95,98,99))

kernel.areas_1perday_trial6=rep(0, length(kernel.d_1perday_trial6))

names(kernel.areas_1perday_trial6)=kernel.d_1perday_trial6

ltys=c(1, 2,3,4,5,6)

lwds=c(1, 1,2,2,3,3)

for(k in 1:length(kernel.d_1perday_trial6)){

ver=getverticeshr(xkernel_1perday_trial6, percent=kernel.d_1perday_trial6[k])

for(i in 1:length(ver@polygons[[1]]@Polygons)){

polygon(ver@polygons[[1]]@Polygons[[i]]@coords, border=grey(level=0.25), col=NA, lty=ltys[k],lwd=lwds[k])

kernel.areas_1perday_trial6[k]=kernel.areas_1perday_trial6[k]+areapl(ver@polygons[[1]]@Polygons[[i]]@coords)/(1000^2)

}

}

kernel.areas_1perday_trial6

####

##Trial 7

####

set.seed(7)

tracks_1perday_trial7<-alltracks %>% group_by(yyyymmdd) %>% sample_n(size = 1)

##Preparing the coordinates for the calculations

utm_1perday_trial7=WGS.to.UTM(long=tracks_1perday_trial7$lon, lat=tracks_1perday_trial7$lat, utm.zone=utm.zone(mean(tracks_1perday_trial7$lon), mean(tracks_1perday_trial7$lat)), avoid.negatives=T, return.zone=F)

xy_1perday_trial7=SpatialPoints(coords=utm_1perday_trial7[, c("long", "lat")])

to.get=c(100, 99, 98, 95, 80, 75, 50)

MCP_1perday_trial7=vector("list", length(to.get))

names(MCP_1perday_trial7)=to.get

for(i in 1:length(to.get)){

xx_1perday_trial7=mcp(xy_1perday_trial7, percent=to.get[i])

MCP_1perday_trial7[[i]]=xx_1perday_trial7@polygons[[1]]@Polygons[[1]]@coords

}

MCP.areas_1perday_trial7=unlist(lapply(MCP_1perday_trial7, areapl))

MCP.areas_1perday_trial7=MCP.areas_1perday_trial7/(1000^2)

names(MCP.areas_1perday_trial7)=to.get

MCP.areas_1perday_trial7

k.data_1perday_trial7=SpatialPointsDataFrame(coords=utm_1perday_trial7, data=data.frame(id=rep("a", nrow(utm_1perday_trial7))))

xkernel_1perday_trial7=kernelUD(k.data_1perday_trial7, h="href", grid=100, extent=1)

##plot:

par(mar=rep(0.2, 4))

plot(as.matrix(MCP_1perday_trial7[["100"]]), asp=1, xlab="", ylab="",xlim=c(548350, 557873),ylim=c(9765030, 9774195), axes=T, type="n", xaxt="n", yaxt="n", bty="n")

cols=c("mediumaquamarine", "gray20", "gray30", "gray60","gray80", "gray90", "gray95")

for(i in 1:length(to.get)){

polygon(MCP_1perday_trial7[[i]], border=NA, col=cols[i])

}

for(i in 1:length(savannah)){

polygon(savannah[[i]], border=NA, col=adjustcolor(col="yellow", alpha.f=0.4))

}

polygon(swamp[[10]], border=NA, col=grey(level=0.4, alpha=0.4))

polygon(sea[[1]], border=NA, col="lightsteelblue2")

#add kernel:

kernel.d_1perday_trial7=rev(c(50, 75, 80, 95,98,99))

kernel.areas_1perday_trial7=rep(0, length(kernel.d_1perday_trial7))

names(kernel.areas_1perday_trial7)=kernel.d_1perday_trial7

ltys=c(1, 2,3,4,5,6)

lwds=c(1, 1,2,2,3,3)

for(k in 1:length(kernel.d_1perday_trial7)){

ver=getverticeshr(xkernel_1perday_trial7, percent=kernel.d_1perday_trial7[k])

for(i in 1:length(ver@polygons[[1]]@Polygons)){

polygon(ver@polygons[[1]]@Polygons[[i]]@coords, border=grey(level=0.25), col=NA, lty=ltys[k],lwd=lwds[k])

kernel.areas_1perday_trial7[k]=kernel.areas_1perday_trial7[k]+areapl(ver@polygons[[1]]@Polygons[[i]]@coords)/(1000^2)

}

}

kernel.areas_1perday_trial7

####

##Trial 8

####

set.seed(8)

tracks_1perday_trial8<-alltracks %>% group_by(yyyymmdd) %>% sample_n(size = 1)

##Preparing the coordinates for the calculations

utm_1perday_trial8=WGS.to.UTM(long=tracks_1perday_trial8$lon, lat=tracks_1perday_trial8$lat, utm.zone=utm.zone(mean(tracks_1perday_trial8$lon), mean(tracks_1perday_trial8$lat)), avoid.negatives=T, return.zone=F)

xy_1perday_trial8=SpatialPoints(coords=utm_1perday_trial8[, c("long", "lat")])

to.get=c(100, 99, 98, 95, 80, 75, 50)

MCP_1perday_trial8=vector("list", length(to.get))

names(MCP_1perday_trial8)=to.get

for(i in 1:length(to.get)){

xx_1perday_trial8=mcp(xy_1perday_trial8, percent=to.get[i])

MCP_1perday_trial8[[i]]=xx_1perday_trial8@polygons[[1]]@Polygons[[1]]@coords

}

MCP.areas_1perday_trial8=unlist(lapply(MCP_1perday_trial8, areapl))

MCP.areas_1perday_trial8=MCP.areas_1perday_trial8/(1000^2)

names(MCP.areas_1perday_trial8)=to.get

MCP.areas_1perday_trial8

k.data_1perday_trial8=SpatialPointsDataFrame(coords=utm_1perday_trial8, data=data.frame(id=rep("a", nrow(utm_1perday_trial8))))

xkernel_1perday_trial8=kernelUD(k.data_1perday_trial8, h="href", grid=100, extent=1)

##plot:

par(mar=rep(0.2, 4))

plot(as.matrix(MCP_1perday_trial8[["100"]]), asp=1, xlab="", ylab="",xlim=c(548350, 557873),ylim=c(9765030, 9774195), axes=T, type="n", xaxt="n", yaxt="n", bty="n")

cols=c("mediumaquamarine", "gray20", "gray30", "gray60","gray80", "gray90", "gray95")

for(i in 1:length(to.get)){

polygon(MCP_1perday_trial8[[i]], border=NA, col=cols[i])

}

for(i in 1:length(savannah)){

polygon(savannah[[i]], border=NA, col=adjustcolor(col="yellow", alpha.f=0.4))

}

polygon(swamp[[10]], border=NA, col=grey(level=0.4, alpha=0.4))

polygon(sea[[1]], border=NA, col="lightsteelblue2")

#add kernel:

kernel.d_1perday_trial8=rev(c(50, 75, 80, 95,98,99))

kernel.areas_1perday_trial8=rep(0, length(kernel.d_1perday_trial8))

names(kernel.areas_1perday_trial8)=kernel.d_1perday_trial8

ltys=c(1, 2,3,4,5,6)

lwds=c(1, 1,2,2,3,3)

for(k in 1:length(kernel.d_1perday_trial8)){

ver=getverticeshr(xkernel_1perday_trial8, percent=kernel.d_1perday_trial8[k])

for(i in 1:length(ver@polygons[[1]]@Polygons)){

polygon(ver@polygons[[1]]@Polygons[[i]]@coords, border=grey(level=0.25), col=NA, lty=ltys[k],lwd=lwds[k])

kernel.areas_1perday_trial8[k]=kernel.areas_1perday_trial8[k]+areapl(ver@polygons[[1]]@Polygons[[i]]@coords)/(1000^2)

}

}

kernel.areas_1perday_trial8

####

##Trial 9

####

set.seed(9)

tracks_1perday_trial9<-alltracks %>% group_by(yyyymmdd) %>% sample_n(size = 1)

##Preparing the coordinates for the calculations

utm_1perday_trial9=WGS.to.UTM(long=tracks_1perday_trial9$lon, lat=tracks_1perday_trial9$lat, utm.zone=utm.zone(mean(tracks_1perday_trial9$lon), mean(tracks_1perday_trial9$lat)), avoid.negatives=T, return.zone=F)

xy_1perday_trial9=SpatialPoints(coords=utm_1perday_trial9[, c("long", "lat")])

to.get=c(100, 99, 98, 95, 80, 75, 50)

MCP_1perday_trial9=vector("list", length(to.get))

names(MCP_1perday_trial9)=to.get

for(i in 1:length(to.get)){

xx_1perday_trial9=mcp(xy_1perday_trial9, percent=to.get[i])

MCP_1perday_trial9[[i]]=xx_1perday_trial9@polygons[[1]]@Polygons[[1]]@coords

}

MCP.areas_1perday_trial9=unlist(lapply(MCP_1perday_trial9, areapl))

MCP.areas_1perday_trial9=MCP.areas_1perday_trial9/(1000^2)

names(MCP.areas_1perday_trial9)=to.get

MCP.areas_1perday_trial9

k.data_1perday_trial9=SpatialPointsDataFrame(coords=utm_1perday_trial9, data=data.frame(id=rep("a", nrow(utm_1perday_trial9))))

xkernel_1perday_trial9=kernelUD(k.data_1perday_trial9, h="href", grid=100, extent=1)

##plot:

par(mar=rep(0.2, 4))

plot(as.matrix(MCP_1perday_trial9[["100"]]), asp=1, xlab="", ylab="",xlim=c(548350, 557873),ylim=c(9765030, 9774195), axes=T, type="n", xaxt="n", yaxt="n", bty="n")

cols=c("mediumaquamarine", "gray20", "gray30", "gray60","gray80", "gray90", "gray95")

for(i in 1:length(to.get)){

polygon(MCP_1perday_trial9[[i]], border=NA, col=cols[i])

}

for(i in 1:length(savannah)){

polygon(savannah[[i]], border=NA, col=adjustcolor(col="yellow", alpha.f=0.4))

}

polygon(swamp[[10]], border=NA, col=grey(level=0.4, alpha=0.4))

polygon(sea[[1]], border=NA, col="lightsteelblue2")

#add kernel:

kernel.d_1perday_trial9=rev(c(50, 75, 80, 95,98,99))

kernel.areas_1perday_trial9=rep(0, length(kernel.d_1perday_trial9))

names(kernel.areas_1perday_trial9)=kernel.d_1perday_trial9

ltys=c(1, 2,3,4,5,6)

lwds=c(1, 1,2,2,3,3)

for(k in 1:length(kernel.d_1perday_trial9)){

ver=getverticeshr(xkernel_1perday_trial9, percent=kernel.d_1perday_trial9[k])

for(i in 1:length(ver@polygons[[1]]@Polygons)){

polygon(ver@polygons[[1]]@Polygons[[i]]@coords, border=grey(level=0.25), col=NA, lty=ltys[k],lwd=lwds[k])

kernel.areas_1perday_trial9[k]=kernel.areas_1perday_trial9[k]+areapl(ver@polygons[[1]]@Polygons[[i]]@coords)/(1000^2)

}

}

kernel.areas_1perday_trial9

####

##Trial 10

####

set.seed(10)

tracks_1perday_trial10<-alltracks %>% group_by(yyyymmdd) %>% sample_n(size = 1)

##Preparing the coordinates for the calculations

utm_1perday_trial10=WGS.to.UTM(long=tracks_1perday_trial10$lon, lat=tracks_1perday_trial10$lat, utm.zone=utm.zone(mean(tracks_1perday_trial10$lon), mean(tracks_1perday_trial10$lat)), avoid.negatives=T, return.zone=F)

xy_1perday_trial10=SpatialPoints(coords=utm_1perday_trial10[, c("long", "lat")])

to.get=c(100, 99, 98, 95, 80, 75, 50)

MCP_1perday_trial10=vector("list", length(to.get))

names(MCP_1perday_trial10)=to.get

for(i in 1:length(to.get)){

xx_1perday_trial10=mcp(xy_1perday_trial10, percent=to.get[i])

MCP_1perday_trial10[[i]]=xx_1perday_trial10@polygons[[1]]@Polygons[[1]]@coords

}

MCP.areas_1perday_trial10=unlist(lapply(MCP_1perday_trial10, areapl))

MCP.areas_1perday_trial10=MCP.areas_1perday_trial10/(1000^2)

names(MCP.areas_1perday_trial10)=to.get

MCP.areas_1perday_trial10

k.data_1perday_trial10=SpatialPointsDataFrame(coords=utm_1perday_trial10, data=data.frame(id=rep("a", nrow(utm_1perday_trial10))))

xkernel_1perday_trial10=kernelUD(k.data_1perday_trial10, h="href", grid=100, extent=1)

##plot:

par(mar=rep(0.2, 4))

plot(as.matrix(MCP_1perday_trial10[["100"]]), asp=1, xlab="", ylab="",xlim=c(548350, 557873),ylim=c(9765030, 9774195), axes=T, type="n", xaxt="n", yaxt="n", bty="n")

cols=c("mediumaquamarine", "gray20", "gray30", "gray60","gray80", "gray90", "gray95")

for(i in 1:length(to.get)){

polygon(MCP_1perday_trial10[[i]], border=NA, col=cols[i])

}

for(i in 1:length(savannah)){

polygon(savannah[[i]], border=NA, col=adjustcolor(col="yellow", alpha.f=0.4))

}

polygon(swamp[[10]], border=NA, col=grey(level=0.4, alpha=0.4))

polygon(sea[[1]], border=NA, col="lightsteelblue2")

#add kernel:

kernel.d_1perday_trial10=rev(c(50, 75, 80, 95,98,99))

kernel.areas_1perday_trial10=rep(0, length(kernel.d_1perday_trial10))

names(kernel.areas_1perday_trial10)=kernel.d_1perday_trial10

ltys=c(1, 2,3,4,5,6)

lwds=c(1, 1,2,2,3,3)

for(k in 1:length(kernel.d_1perday_trial10)){

ver=getverticeshr(xkernel_1perday_trial10, percent=kernel.d_1perday_trial10[k])

for(i in 1:length(ver@polygons[[1]]@Polygons)){

polygon(ver@polygons[[1]]@Polygons[[i]]@coords, border=grey(level=0.25), col=NA, lty=ltys[k],lwd=lwds[k])

kernel.areas_1perday_trial10[k]=kernel.areas_1perday_trial10[k]+areapl(ver@polygons[[1]]@Polygons[[i]]@coords)/(1000^2)

}

}

kernel.areas_1perday_trial10

class(kernel.areas_1perday_trial10)

########################

# 10.3 Calculating MCP and KDE with three samples per day (x10 trials)

########################

####

##Trial 1

####

set.seed(1)

tracks_3perday_trial1<-alltracks %>% group_by(yyyymmdd) %>% sample_n(size = 3)

##Preparing the coordinates for the calculations

utm_3perday_trial1=WGS.to.UTM(long=tracks_3perday_trial1$lon, lat=tracks_3perday_trial1$lat, utm.zone=utm.zone(mean(tracks_3perday_trial1$lon), mean(tracks_3perday_trial1$lat)), avoid.negatives=T, return.zone=F)

xy_3perday_trial1=SpatialPoints(coords=utm_3perday_trial1[, c("long", "lat")])

to.get=c(100, 99, 98, 95, 80, 75, 50)

MCP_3perday_trial1=vector("list", length(to.get))

names(MCP_3perday_trial1)=to.get

for(i in 1:length(to.get)){

xx_3perday_trial1=mcp(xy_3perday_trial1, percent=to.get[i])

MCP_3perday_trial1[[i]]=xx_3perday_trial1@polygons[[1]]@Polygons[[1]]@coords

}

MCP.areas_3perday_trial1=unlist(lapply(MCP_3perday_trial1, areapl))

MCP.areas_3perday_trial1=MCP.areas_3perday_trial1/(1000^2)

names(MCP.areas_3perday_trial1)=to.get

MCP.areas_3perday_trial1

k.data_3perday_trial1=SpatialPointsDataFrame(coords=utm_3perday_trial1, data=data.frame(id=rep("a", nrow(utm_3perday_trial1))))

xkernel_3perday_trial1=kernelUD(k.data_3perday_trial1, h="href", grid=100, extent=1)

##plot:

par(mar=rep(0.2, 4))

plot(as.matrix(MCP_3perday_trial1[["100"]]), asp=1, xlab="", ylab="",xlim=c(548350, 557873),ylim=c(9765030, 9774195), axes=T, type="n", xaxt="n", yaxt="n", bty="n")

cols=c("mediumaquamarine", "gray20", "gray30", "gray60","gray80", "gray90", "gray95")

for(i in 1:length(to.get)){

polygon(MCP_3perday_trial1[[i]], border=NA, col=cols[i])

}

for(i in 1:length(savannah)){

polygon(savannah[[i]], border=NA, col=adjustcolor(col="yellow", alpha.f=0.4))

}

polygon(swamp[[10]], border=NA, col=grey(level=0.4, alpha=0.4))

polygon(sea[[1]], border=NA, col="lightsteelblue2")

#add kernel:

kernel.d_3perday_trial1=rev(c(50, 75, 80, 95,98,99))

kernel.areas_3perday_trial1=rep(0, length(kernel.d_3perday_trial1))

names(kernel.areas_3perday_trial1)=kernel.d_3perday_trial1

ltys=c(1, 2,3,4,5,6)

lwds=c(1, 1,2,2,3,3)

for(k in 1:length(kernel.d_3perday_trial1)){

ver=getverticeshr(xkernel_3perday_trial1, percent=kernel.d_3perday_trial1[k])

for(i in 1:length(ver@polygons[[1]]@Polygons)){

polygon(ver@polygons[[1]]@Polygons[[i]]@coords, border=grey(level=0.25), col=NA, lty=ltys[k],lwd=lwds[k])

kernel.areas_3perday_trial1[k]=kernel.areas_3perday_trial1[k]+areapl(ver@polygons[[1]]@Polygons[[i]]@coords)/(1000^2)

}

}

kernel.areas_3perday_trial1

####

##Trial 2

####

set.seed(2)

tracks_3perday_trial2<-alltracks %>% group_by(yyyymmdd) %>% sample_n(size = 3)

##Preparing the coordinates for the calculations

utm_3perday_trial2=WGS.to.UTM(long=tracks_3perday_trial2$lon, lat=tracks_3perday_trial2$lat, utm.zone=utm.zone(mean(tracks_3perday_trial2$lon), mean(tracks_3perday_trial2$lat)), avoid.negatives=T, return.zone=F)

xy_3perday_trial2=SpatialPoints(coords=utm_3perday_trial2[, c("long", "lat")])

to.get=c(100, 99, 98, 95, 80, 75, 50)

MCP_3perday_trial2=vector("list", length(to.get))

names(MCP_3perday_trial2)=to.get

for(i in 1:length(to.get)){

xx_3perday_trial2=mcp(xy_3perday_trial2, percent=to.get[i])

MCP_3perday_trial2[[i]]=xx_3perday_trial2@polygons[[1]]@Polygons[[1]]@coords

}

MCP.areas_3perday_trial2=unlist(lapply(MCP_3perday_trial2, areapl))

MCP.areas_3perday_trial2=MCP.areas_3perday_trial2/(1000^2)

names(MCP.areas_3perday_trial2)=to.get

MCP.areas_3perday_trial2

k.data_3perday_trial2=SpatialPointsDataFrame(coords=utm_3perday_trial2, data=data.frame(id=rep("a", nrow(utm_3perday_trial2))))

xkernel_3perday_trial2=kernelUD(k.data_3perday_trial2, h="href", grid=100, extent=1)

##plot:

par(mar=rep(0.2, 4))

plot(as.matrix(MCP_3perday_trial2[["100"]]), asp=1, xlab="", ylab="",xlim=c(548350, 557873),ylim=c(9765030, 9774195), axes=T, type="n", xaxt="n", yaxt="n", bty="n")

cols=c("mediumaquamarine", "gray20", "gray30", "gray60","gray80", "gray90", "gray95")

for(i in 1:length(to.get)){

polygon(MCP_3perday_trial2[[i]], border=NA, col=cols[i])

}

for(i in 1:length(savannah)){

polygon(savannah[[i]], border=NA, col=adjustcolor(col="yellow", alpha.f=0.4))

}

polygon(swamp[[10]], border=NA, col=grey(level=0.4, alpha=0.4))

polygon(sea[[1]], border=NA, col="lightsteelblue2")

#add kernel:

kernel.d_3perday_trial2=rev(c(50, 75, 80, 95,98,99))

kernel.areas_3perday_trial2=rep(0, length(kernel.d_3perday_trial2))

names(kernel.areas_3perday_trial2)=kernel.d_3perday_trial2

ltys=c(1, 2,3,4,5,6)

lwds=c(1, 1,2,2,3,3)

for(k in 1:length(kernel.d_3perday_trial2)){

ver=getverticeshr(xkernel_3perday_trial2, percent=kernel.d_3perday_trial2[k])

for(i in 1:length(ver@polygons[[1]]@Polygons)){

polygon(ver@polygons[[1]]@Polygons[[i]]@coords, border=grey(level=0.25), col=NA, lty=ltys[k],lwd=lwds[k])

kernel.areas_3perday_trial2[k]=kernel.areas_3perday_trial2[k]+areapl(ver@polygons[[1]]@Polygons[[i]]@coords)/(1000^2)

}

}

kernel.areas_3perday_trial2

####

##Trial 3

####

set.seed(3)

tracks_3perday_trial3<-alltracks %>% group_by(yyyymmdd) %>% sample_n(size = 3)##Preparing the coordinates for the calculationsutm_3perday_trial3=WGS.to.UTM(long=tracks_3perday_trial3$lon, lat=tracks_3perday_trial3$lat, utm.zone=utm.zone(mean(tracks_3perday_trial3$lon), mean(tracks_3perday_trial3$lat)), avoid.negatives=T, return.zone=F)

xy_3perday_trial3=SpatialPoints(coords=utm_3perday_trial3[, c("long", "lat")])

to.get=c(100, 99, 98, 95, 80, 75, 50)

MCP_3perday_trial3=vector("list", length(to.get))

names(MCP_3perday_trial3)=to.get

for(i in 1:length(to.get)){

xx_3perday_trial3=mcp(xy_3perday_trial3, percent=to.get[i])

MCP_3perday_trial3[[i]]=xx_3perday_trial3@polygons[[1]]@Polygons[[1]]@coords

}

MCP.areas_3perday_trial3=unlist(lapply(MCP_3perday_trial3, areapl))

MCP.areas_3perday_trial3=MCP.areas_3perday_trial3/(1000^2)

names(MCP.areas_3perday_trial3)=to.get

MCP.areas_3perday_trial3

k.data_3perday_trial3=SpatialPointsDataFrame(coords=utm_3perday_trial3, data=data.frame(id=rep("a", nrow(utm_3perday_trial3))))

xkernel_3perday_trial3=kernelUD(k.data_3perday_trial3, h="href", grid=100, extent=1)

##plot:

par(mar=rep(0.2, 4))

plot(as.matrix(MCP_3perday_trial3[["100"]]), asp=1, xlab="", ylab="",xlim=c(548350, 557873),ylim=c(9765030, 9774195), axes=T, type="n", xaxt="n", yaxt="n", bty="n")

cols=c("mediumaquamarine", "gray20", "gray30", "gray60","gray80", "gray90", "gray95")

for(i in 1:length(to.get)){

polygon(MCP_3perday_trial3[[i]], border=NA, col=cols[i])

}

for(i in 1:length(savannah)){

polygon(savannah[[i]], border=NA, col=adjustcolor(col="yellow", alpha.f=0.4))

}

polygon(swamp[[10]], border=NA, col=grey(level=0.4, alpha=0.4))

polygon(sea[[1]], border=NA, col="lightsteelblue2")

#add kernel:

kernel.d_3perday_trial3=rev(c(50, 75, 80, 95,98,99))

kernel.areas_3perday_trial3=rep(0, length(kernel.d_3perday_trial3))

names(kernel.areas_3perday_trial3)=kernel.d_3perday_trial3

ltys=c(1, 2,3,4,5,6)

lwds=c(1, 1,2,2,3,3)

for(k in 1:length(kernel.d_3perday_trial3)){

ver=getverticeshr(xkernel_3perday_trial3, percent=kernel.d_3perday_trial3[k])

for(i in 1:length(ver@polygons[[1]]@Polygons)){

polygon(ver@polygons[[1]]@Polygons[[i]]@coords, border=grey(level=0.25), col=NA, lty=ltys[k],lwd=lwds[k])

kernel.areas_3perday_trial3[k]=kernel.areas_3perday_trial3[k]+areapl(ver@polygons[[1]]@Polygons[[i]]@coords)/(1000^2)

}

}

kernel.areas_3perday_trial3

####

##Trial 4

####

set.seed(4)

tracks_3perday_trial4<-alltracks %>% group_by(yyyymmdd) %>% sample_n(size = 3)

##Preparing the coordinates for the calculations

utm_3perday_trial4=WGS.to.UTM(long=tracks_3perday_trial4$lon, lat=tracks_3perday_trial4$lat, utm.zone=utm.zone(mean(tracks_3perday_trial4$lon), mean(tracks_3perday_trial4$lat)), avoid.negatives=T, return.zone=F)

xy_3perday_trial4=SpatialPoints(coords=utm_3perday_trial4[, c("long", "lat")])

to.get=c(100, 99, 98, 95, 80, 75, 50)

MCP_3perday_trial4=vector("list", length(to.get))

names(MCP_3perday_trial4)=to.get

for(i in 1:length(to.get)){

xx_3perday_trial4=mcp(xy_3perday_trial4, percent=to.get[i])

MCP_3perday_trial4[[i]]=xx_3perday_trial4@polygons[[1]]@Polygons[[1]]@coords

}

MCP.areas_3perday_trial4=unlist(lapply(MCP_3perday_trial4, areapl))

MCP.areas_3perday_trial4=MCP.areas_3perday_trial4/(1000^2)

names(MCP.areas_3perday_trial4)=to.get

MCP.areas_3perday_trial4

k.data_3perday_trial4=SpatialPointsDataFrame(coords=utm_3perday_trial4, data=data.frame(id=rep("a", nrow(utm_3perday_trial4))))

xkernel_3perday_trial4=kernelUD(k.data_3perday_trial4, h="href", grid=100, extent=1)

##plot:

par(mar=rep(0.2, 4))

plot(as.matrix(MCP_3perday_trial4[["100"]]), asp=1, xlab="", ylab="",xlim=c(548350, 557873),ylim=c(9765030, 9774195), axes=T, type="n", xaxt="n", yaxt="n", bty="n")

cols=c("mediumaquamarine", "gray20", "gray30", "gray60","gray80", "gray90", "gray95")

for(i in 1:length(to.get)){

polygon(MCP_3perday_trial4[[i]], border=NA, col=cols[i])

}

for(i in 1:length(savannah)){

polygon(savannah[[i]], border=NA, col=adjustcolor(col="yellow", alpha.f=0.4))

}

polygon(swamp[[10]], border=NA, col=grey(level=0.4, alpha=0.4))

polygon(sea[[1]], border=NA, col="lightsteelblue2")

#add kernel:

kernel.d_3perday_trial4=rev(c(50, 75, 80, 95,98,99))

kernel.areas_3perday_trial4=rep(0, length(kernel.d_3perday_trial4))

names(kernel.areas_3perday_trial4)=kernel.d_3perday_trial4

ltys=c(1, 2,3,4,5,6)

lwds=c(1, 1,2,2,3,3)

for(k in 1:length(kernel.d_3perday_trial4)){

ver=getverticeshr(xkernel_3perday_trial4, percent=kernel.d_3perday_trial4[k])

for(i in 1:length(ver@polygons[[1]]@Polygons)){

polygon(ver@polygons[[1]]@Polygons[[i]]@coords, border=grey(level=0.25), col=NA, lty=ltys[k],lwd=lwds[k])

kernel.areas_3perday_trial4[k]=kernel.areas_3perday_trial4[k]+areapl(ver@polygons[[1]]@Polygons[[i]]@coords)/(1000^2)

}

}

kernel.areas_3perday_trial4

####

##Trial 5

####

set.seed(5)

tracks_3perday_trial5<-alltracks %>% group_by(yyyymmdd) %>% sample_n(size = 3)

##Preparing the coordinates for the calculations

utm_3perday_trial5=WGS.to.UTM(long=tracks_3perday_trial5$lon, lat=tracks_3perday_trial5$lat, utm.zone=utm.zone(mean(tracks_3perday_trial5$lon), mean(tracks_3perday_trial5$lat)), avoid.negatives=T, return.zone=F)

xy_3perday_trial5=SpatialPoints(coords=utm_3perday_trial5[, c("long", "lat")])

to.get=c(100, 99, 98, 95, 80, 75, 50)

MCP_3perday_trial5=vector("list", length(to.get))

names(MCP_3perday_trial5)=to.get

for(i in 1:length(to.get)){

xx_3perday_trial5=mcp(xy_3perday_trial5, percent=to.get[i])

MCP_3perday_trial5[[i]]=xx_3perday_trial5@polygons[[1]]@Polygons[[1]]@coords

}

MCP.areas_3perday_trial5=unlist(lapply(MCP_3perday_trial5, areapl))

MCP.areas_3perday_trial5=MCP.areas_3perday_trial5/(1000^2)

names(MCP.areas_3perday_trial5)=to.get

MCP.areas_3perday_trial5

k.data_3perday_trial5=SpatialPointsDataFrame(coords=utm_3perday_trial5, data=data.frame(id=rep("a", nrow(utm_3perday_trial5))))

xkernel_3perday_trial5=kernelUD(k.data_3perday_trial5, h="href", grid=100, extent=1)

##plot:

par(mar=rep(0.2, 4))

plot(as.matrix(MCP_3perday_trial5[["100"]]), asp=1, xlab="", ylab="",xlim=c(548350, 557873),ylim=c(9765030, 9774195), axes=T, type="n", xaxt="n", yaxt="n", bty="n")

cols=c("mediumaquamarine", "gray20", "gray30", "gray60","gray80", "gray90", "gray95")

for(i in 1:length(to.get)){

polygon(MCP_3perday_trial5[[i]], border=NA, col=cols[i])

}

for(i in 1:length(savannah)){

polygon(savannah[[i]], border=NA, col=adjustcolor(col="yellow", alpha.f=0.4))

}

polygon(swamp[[10]], border=NA, col=grey(level=0.4, alpha=0.4))

polygon(sea[[1]], border=NA, col="lightsteelblue2")

#add kernel:

kernel.d_3perday_trial5=rev(c(50, 75, 80, 95,98,99))

kernel.areas_3perday_trial5=rep(0, length(kernel.d_3perday_trial5))

names(kernel.areas_3perday_trial5)=kernel.d_3perday_trial5

ltys=c(1, 2,3,4,5,6)

lwds=c(1, 1,2,2,3,3)

for(k in 1:length(kernel.d_3perday_trial5)){

ver=getverticeshr(xkernel_3perday_trial5, percent=kernel.d_3perday_trial5[k])

for(i in 1:length(ver@polygons[[1]]@Polygons)){

polygon(ver@polygons[[1]]@Polygons[[i]]@coords, border=grey(level=0.25), col=NA, lty=ltys[k],lwd=lwds[k])

kernel.areas_3perday_trial5[k]=kernel.areas_3perday_trial5[k]+areapl(ver@polygons[[1]]@Polygons[[i]]@coords)/(1000^2)

}

}

kernel.areas_3perday_trial5

####

##Trial 6

####

set.seed(6)

tracks_3perday_trial6<-alltracks %>% group_by(yyyymmdd) %>% sample_n(size = 3)

##Preparing the coordinates for the calculations

utm_3perday_trial6=WGS.to.UTM(long=tracks_3perday_trial6$lon, lat=tracks_3perday_trial6$lat, utm.zone=utm.zone(mean(tracks_3perday_trial6$lon), mean(tracks_3perday_trial6$lat)), avoid.negatives=T, return.zone=F)

xy_3perday_trial6=SpatialPoints(coords=utm_3perday_trial6[, c("long", "lat")])

to.get=c(100, 99, 98, 95, 80, 75, 50)

MCP_3perday_trial6=vector("list", length(to.get))

names(MCP_3perday_trial6)=to.get

for(i in 1:length(to.get)){

xx_3perday_trial6=mcp(xy_3perday_trial6, percent=to.get[i])

MCP_3perday_trial6[[i]]=xx_3perday_trial6@polygons[[1]]@Polygons[[1]]@coords

}

MCP.areas_3perday_trial6=unlist(lapply(MCP_3perday_trial6, areapl))

MCP.areas_3perday_trial6=MCP.areas_3perday_trial6/(1000^2)

names(MCP.areas_3perday_trial6)=to.get

MCP.areas_3perday_trial6

k.data_3perday_trial6=SpatialPointsDataFrame(coords=utm_3perday_trial6, data=data.frame(id=rep("a", nrow(utm_3perday_trial6))))

xkernel_3perday_trial6=kernelUD(k.data_3perday_trial6, h="href", grid=100, extent=1)

##plot:

par(mar=rep(0.2, 4))

plot(as.matrix(MCP_3perday_trial6[["100"]]), asp=1, xlab="", ylab="",xlim=c(548350, 557873),ylim=c(9765030, 9774195), axes=T, type="n", xaxt="n", yaxt="n", bty="n")

cols=c("mediumaquamarine", "gray20", "gray30", "gray60","gray80", "gray90", "gray95")

for(i in 1:length(to.get)){

polygon(MCP_3perday_trial6[[i]], border=NA, col=cols[i])

}

for(i in 1:length(savannah)){

polygon(savannah[[i]], border=NA, col=adjustcolor(col="yellow", alpha.f=0.4))

}

polygon(swamp[[10]], border=NA, col=grey(level=0.4, alpha=0.4))

polygon(sea[[1]], border=NA, col="lightsteelblue2")

#add kernel:

kernel.d_3perday_trial6=rev(c(50, 75, 80, 95,98,99))

kernel.areas_3perday_trial6=rep(0, length(kernel.d_3perday_trial6))

names(kernel.areas_3perday_trial6)=kernel.d_3perday_trial6

ltys=c(1, 2,3,4,5,6)

lwds=c(1, 1,2,2,3,3)

for(k in 1:length(kernel.d_3perday_trial6)){

ver=getverticeshr(xkernel_3perday_trial6, percent=kernel.d_3perday_trial6[k])

for(i in 1:length(ver@polygons[[1]]@Polygons)){

polygon(ver@polygons[[1]]@Polygons[[i]]@coords, border=grey(level=0.25), col=NA, lty=ltys[k],lwd=lwds[k])

kernel.areas_3perday_trial6[k]=kernel.areas_3perday_trial6[k]+areapl(ver@polygons[[1]]@Polygons[[i]]@coords)/(1000^2)

}

}

kernel.areas_3perday_trial6

####

##Trial 7

####

set.seed(7)

tracks_3perday_trial7<-alltracks %>% group_by(yyyymmdd) %>% sample_n(size = 3)

##Preparing the coordinates for the calculations

utm_3perday_trial7=WGS.to.UTM(long=tracks_3perday_trial7$lon, lat=tracks_3perday_trial7$lat, utm.zone=utm.zone(mean(tracks_3perday_trial7$lon), mean(tracks_3perday_trial7$lat)), avoid.negatives=T, return.zone=F)

xy_3perday_trial7=SpatialPoints(coords=utm_3perday_trial7[, c("long", "lat")])

to.get=c(100, 99, 98, 95, 80, 75, 50)

MCP_3perday_trial7=vector("list", length(to.get))

names(MCP_3perday_trial7)=to.get

for(i in 1:length(to.get)){

xx_3perday_trial7=mcp(xy_3perday_trial7, percent=to.get[i])

MCP_3perday_trial7[[i]]=xx_3perday_trial7@polygons[[1]]@Polygons[[1]]@coords

}

MCP.areas_3perday_trial7=unlist(lapply(MCP_3perday_trial7, areapl))

MCP.areas_3perday_trial7=MCP.areas_3perday_trial7/(1000^2)

names(MCP.areas_3perday_trial7)=to.get

MCP.areas_3perday_trial7

k.data_3perday_trial7=SpatialPointsDataFrame(coords=utm_3perday_trial7, data=data.frame(id=rep("a", nrow(utm_3perday_trial7))))

xkernel_3perday_trial7=kernelUD(k.data_3perday_trial7, h="href", grid=100, extent=1)

##plot:

par(mar=rep(0.2, 4))

plot(as.matrix(MCP_3perday_trial7[["100"]]), asp=1, xlab="", ylab="",xlim=c(548350, 557873),ylim=c(9765030, 9774195), axes=T, type="n", xaxt="n", yaxt="n", bty="n")

cols=c("mediumaquamarine", "gray20", "gray30", "gray60","gray80", "gray90", "gray95")

for(i in 1:length(to.get)){

polygon(MCP_3perday_trial7[[i]], border=NA, col=cols[i])

}

for(i in 1:length(savannah)){

polygon(savannah[[i]], border=NA, col=adjustcolor(col="yellow", alpha.f=0.4))

}

polygon(swamp[[10]], border=NA, col=grey(level=0.4, alpha=0.4))

polygon(sea[[1]], border=NA, col="lightsteelblue2")

#add kernel:

kernel.d_3perday_trial7=rev(c(50, 75, 80, 95,98,99))

kernel.areas_3perday_trial7=rep(0, length(kernel.d_3perday_trial7))

names(kernel.areas_3perday_trial7)=kernel.d_3perday_trial7

ltys=c(1, 2,3,4,5,6)

lwds=c(1, 1,2,2,3,3)

for(k in 1:length(kernel.d_3perday_trial7)){

ver=getverticeshr(xkernel_3perday_trial7, percent=kernel.d_3perday_trial7[k])

for(i in 1:length(ver@polygons[[1]]@Polygons)){

polygon(ver@polygons[[1]]@Polygons[[i]]@coords, border=grey(level=0.25), col=NA, lty=ltys[k],lwd=lwds[k])

kernel.areas_3perday_trial7[k]=kernel.areas_3perday_trial7[k]+areapl(ver@polygons[[1]]@Polygons[[i]]@coords)/(1000^2)

}

}

kernel.areas_3perday_trial7

####

##Trial 8

####

set.seed(8)

tracks_3perday_trial8<-alltracks %>% group_by(yyyymmdd) %>% sample_n(size = 3)

##Preparing the coordinates for the calculations

utm_3perday_trial8=WGS.to.UTM(long=tracks_3perday_trial8$lon, lat=tracks_3perday_trial8$lat, utm.zone=utm.zone(mean(tracks_3perday_trial8$lon), mean(tracks_3perday_trial8$lat)), avoid.negatives=T, return.zone=F)

xy_3perday_trial8=SpatialPoints(coords=utm_3perday_trial8[, c("long", "lat")])

to.get=c(100, 99, 98, 95, 80, 75, 50)

MCP_3perday_trial8=vector("list", length(to.get))

names(MCP_3perday_trial8)=to.get

for(i in 1:length(to.get)){

xx_3perday_trial8=mcp(xy_3perday_trial8, percent=to.get[i])

MCP_3perday_trial8[[i]]=xx_3perday_trial8@polygons[[1]]@Polygons[[1]]@coords

}

MCP.areas_3perday_trial8=unlist(lapply(MCP_3perday_trial8, areapl))

MCP.areas_3perday_trial8=MCP.areas_3perday_trial8/(1000^2)

names(MCP.areas_3perday_trial8)=to.get

MCP.areas_3perday_trial8

k.data_3perday_trial8=SpatialPointsDataFrame(coords=utm_3perday_trial8, data=data.frame(id=rep("a", nrow(utm_3perday_trial8))))

xkernel_3perday_trial8=kernelUD(k.data_3perday_trial8, h="href", grid=100, extent=1)

##plot:

par(mar=rep(0.2, 4))

plot(as.matrix(MCP_3perday_trial8[["100"]]), asp=1, xlab="", ylab="",xlim=c(548350, 557873),ylim=c(9765030, 9774195), axes=T, type="n", xaxt="n", yaxt="n", bty="n")

cols=c("mediumaquamarine", "gray20", "gray30", "gray60","gray80", "gray90", "gray95")

for(i in 1:length(to.get)){

polygon(MCP_3perday_trial8[[i]], border=NA, col=cols[i])

}

for(i in 1:length(savannah)){

polygon(savannah[[i]], border=NA, col=adjustcolor(col="yellow", alpha.f=0.4))

}

polygon(swamp[[10]], border=NA, col=grey(level=0.4, alpha=0.4))

polygon(sea[[1]], border=NA, col="lightsteelblue2")

#add kernel:

kernel.d_3perday_trial8=rev(c(50, 75, 80, 95,98,99))

kernel.areas_3perday_trial8=rep(0, length(kernel.d_3perday_trial8))

names(kernel.areas_3perday_trial8)=kernel.d_3perday_trial8

ltys=c(1, 2,3,4,5,6)

lwds=c(1, 1,2,2,3,3)

for(k in 1:length(kernel.d_3perday_trial8)){

ver=getverticeshr(xkernel_3perday_trial8, percent=kernel.d_3perday_trial8[k])

for(i in 1:length(ver@polygons[[1]]@Polygons)){

polygon(ver@polygons[[1]]@Polygons[[i]]@coords, border=grey(level=0.25), col=NA, lty=ltys[k],lwd=lwds[k])

kernel.areas_3perday_trial8[k]=kernel.areas_3perday_trial8[k]+areapl(ver@polygons[[1]]@Polygons[[i]]@coords)/(1000^2)

}

}

kernel.areas_3perday_trial8

####

##Trial 9

####

set.seed(9)

tracks_3perday_trial9<-alltracks %>% group_by(yyyymmdd) %>% sample_n(size = 3)

##Preparing the coordinates for the calculations

utm_3perday_trial9=WGS.to.UTM(long=tracks_3perday_trial9$lon, lat=tracks_3perday_trial9$lat, utm.zone=utm.zone(mean(tracks_3perday_trial9$lon), mean(tracks_3perday_trial9$lat)), avoid.negatives=T, return.zone=F)

xy_3perday_trial9=SpatialPoints(coords=utm_3perday_trial9[, c("long", "lat")])

to.get=c(100, 99, 98, 95, 80, 75, 50)

MCP_3perday_trial9=vector("list", length(to.get))

names(MCP_3perday_trial9)=to.get

for(i in 1:length(to.get)){

xx_3perday_trial9=mcp(xy_3perday_trial9, percent=to.get[i])

MCP_3perday_trial9[[i]]=xx_3perday_trial9@polygons[[1]]@Polygons[[1]]@coords

}

MCP.areas_3perday_trial9=unlist(lapply(MCP_3perday_trial9, areapl))

MCP.areas_3perday_trial9=MCP.areas_3perday_trial9/(1000^2)

names(MCP.areas_3perday_trial9)=to.get

MCP.areas_3perday_trial9

k.data_3perday_trial9=SpatialPointsDataFrame(coords=utm_3perday_trial9, data=data.frame(id=rep("a", nrow(utm_3perday_trial9))))

xkernel_3perday_trial9=kernelUD(k.data_3perday_trial9, h="href", grid=100, extent=1)

##plot:

par(mar=rep(0.2, 4))

plot(as.matrix(MCP_3perday_trial9[["100"]]), asp=1, xlab="", ylab="",xlim=c(548350, 557873),ylim=c(9765030, 9774195), axes=T, type="n", xaxt="n", yaxt="n", bty="n")

cols=c("mediumaquamarine", "gray20", "gray30", "gray60","gray80", "gray90", "gray95")

for(i in 1:length(to.get)){

polygon(MCP_3perday_trial9[[i]], border=NA, col=cols[i])

}

for(i in 1:length(savannah)){

polygon(savannah[[i]], border=NA, col=adjustcolor(col="yellow", alpha.f=0.4))

}

polygon(swamp[[10]], border=NA, col=grey(level=0.4, alpha=0.4))

polygon(sea[[1]], border=NA, col="lightsteelblue2")

#add kernel:

kernel.d_3perday_trial9=rev(c(50, 75, 80, 95,98,99))

kernel.areas_3perday_trial9=rep(0, length(kernel.d_3perday_trial9))

names(kernel.areas_3perday_trial9)=kernel.d_3perday_trial9

ltys=c(1, 2,3,4,5,6)

lwds=c(1, 1,2,2,3,3)

for(k in 1:length(kernel.d_3perday_trial9)){

ver=getverticeshr(xkernel_3perday_trial9, percent=kernel.d_3perday_trial9[k])

for(i in 1:length(ver@polygons[[1]]@Polygons)){

polygon(ver@polygons[[1]]@Polygons[[i]]@coords, border=grey(level=0.25), col=NA, lty=ltys[k],lwd=lwds[k])

kernel.areas_3perday_trial9[k]=kernel.areas_3perday_trial9[k]+areapl(ver@polygons[[1]]@Polygons[[i]]@coords)/(1000^2)

}

}

kernel.areas_3perday_trial9

####

##Trial 10

####

set.seed(10)

tracks_3perday_trial10<-alltracks %>% group_by(yyyymmdd) %>% sample_n(size = 3)

##Preparing the coordinates for the calculations

utm_3perday_trial10=WGS.to.UTM(long=tracks_3perday_trial10$lon, lat=tracks_3perday_trial10$lat, utm.zone=utm.zone(mean(tracks_3perday_trial10$lon), mean(tracks_3perday_trial10$lat)), avoid.negatives=T, return.zone=F)

xy_3perday_trial10=SpatialPoints(coords=utm_3perday_trial10[, c("long", "lat")])

to.get=c(100, 99, 98, 95, 80, 75, 50)

MCP_3perday_trial10=vector("list", length(to.get))

names(MCP_3perday_trial10)=to.get

for(i in 1:length(to.get)){

xx_3perday_trial10=mcp(xy_3perday_trial10, percent=to.get[i])

MCP_3perday_trial10[[i]]=xx_3perday_trial10@polygons[[1]]@Polygons[[1]]@coords

}

MCP.areas_3perday_trial10=unlist(lapply(MCP_3perday_trial10, areapl))

MCP.areas_3perday_trial10=MCP.areas_3perday_trial10/(1000^2)

names(MCP.areas_3perday_trial10)=to.get

MCP.areas_3perday_trial10

k.data_3perday_trial10=SpatialPointsDataFrame(coords=utm_3perday_trial10, data=data.frame(id=rep("a", nrow(utm_3perday_trial10))))

xkernel_3perday_trial10=kernelUD(k.data_3perday_trial10, h="href", grid=100, extent=1)

##plot:

par(mar=rep(0.2, 4))

plot(as.matrix(MCP_3perday_trial10[["100"]]), asp=1, xlab="", ylab="",xlim=c(548350, 557873),ylim=c(9765030, 9774195), axes=T, type="n", xaxt="n", yaxt="n", bty="n")

cols=c("mediumaquamarine", "gray20", "gray30", "gray60","gray80", "gray90", "gray95")

for(i in 1:length(to.get)){

polygon(MCP_3perday_trial10[[i]], border=NA, col=cols[i])

}

for(i in 1:length(savannah)){

polygon(savannah[[i]], border=NA, col=adjustcolor(col="yellow", alpha.f=0.4))

}

polygon(swamp[[10]], border=NA, col=grey(level=0.4, alpha=0.4))

polygon(sea[[1]], border=NA, col="lightsteelblue2")

#add kernel:

kernel.d_3perday_trial10=rev(c(50, 75, 80, 95,98,99))

kernel.areas_3perday_trial10=rep(0, length(kernel.d_3perday_trial10))

names(kernel.areas_3perday_trial10)=kernel.d_3perday_trial10

ltys=c(1, 2,3,4,5,6)

lwds=c(1, 1,2,2,3,3)

for(k in 1:length(kernel.d_3perday_trial10)){

ver=getverticeshr(xkernel_3perday_trial10, percent=kernel.d_3perday_trial10[k])

for(i in 1:length(ver@polygons[[1]]@Polygons)){

polygon(ver@polygons[[1]]@Polygons[[i]]@coords, border=grey(level=0.25), col=NA, lty=ltys[k],lwd=lwds[k])

kernel.areas_3perday_trial10[k]=kernel.areas_3perday_trial10[k]+areapl(ver@polygons[[1]]@Polygons[[i]]@coords)/(1000^2)

}

}

kernel.areas_3perday_trial10

########################

# 10.4 Calculating MCP and KDE with twelve samples per day (x10 trials)

############################

##Trial 1

####

set.seed(1)

tracks_12perday_trial1<-alltracks %>% group_by(yyyymmdd) %>% sample_n(size = 12)

##Preparing the coordinates for the calculations

utm_12perday_trial1=WGS.to.UTM(long=tracks_12perday_trial1$lon, lat=tracks_12perday_trial1$lat, utm.zone=utm.zone(mean(tracks_12perday_trial1$lon), mean(tracks_12perday_trial1$lat)), avoid.negatives=T, return.zone=F)

xy_12perday_trial1=SpatialPoints(coords=utm_12perday_trial1[, c("long", "lat")])

to.get=c(100, 99, 98, 95, 80, 75, 50)

MCP_12perday_trial1=vector("list", length(to.get))

names(MCP_12perday_trial1)=to.get

for(i in 1:length(to.get)){

xx_12perday_trial1=mcp(xy_12perday_trial1, percent=to.get[i])

MCP_12perday_trial1[[i]]=xx_12perday_trial1@polygons[[1]]@Polygons[[1]]@coords

}

MCP.areas_12perday_trial1=unlist(lapply(MCP_12perday_trial1, areapl))

MCP.areas_12perday_trial1=MCP.areas_12perday_trial1/(1000^2)

names(MCP.areas_12perday_trial1)=to.get

MCP.areas_12perday_trial1

k.data_12perday_trial1=SpatialPointsDataFrame(coords=utm_12perday_trial1, data=data.frame(id=rep("a", nrow(utm_12perday_trial1))))

xkernel_12perday_trial1=kernelUD(k.data_12perday_trial1, h="href", grid=100, extent=1)

##plot:

par(mar=rep(0.2, 4))

plot(as.matrix(MCP_12perday_trial1[["100"]]), asp=1, xlab="", ylab="",xlim=c(548350, 557873),ylim=c(9765030, 9774195), axes=T, type="n", xaxt="n", yaxt="n", bty="n")

cols=c("mediumaquamarine", "gray20", "gray30", "gray60","gray80", "gray90", "gray95")

for(i in 1:length(to.get)){

polygon(MCP_12perday_trial1[[i]], border=NA, col=cols[i])

}

for(i in 1:length(savannah)){

polygon(savannah[[i]], border=NA, col=adjustcolor(col="yellow", alpha.f=0.4))

}

polygon(swamp[[10]], border=NA, col=grey(level=0.4, alpha=0.4))

polygon(sea[[1]], border=NA, col="lightsteelblue2")

#add kernel:

kernel.d_12perday_trial1=rev(c(50, 75, 80, 95,98,99))

kernel.areas_12perday_trial1=rep(0, length(kernel.d_12perday_trial1))

names(kernel.areas_12perday_trial1)=kernel.d_12perday_trial1

ltys=c(1, 2,3,4,5,6)

lwds=c(1, 1,2,2,3,3)

for(k in 1:length(kernel.d_12perday_trial1)){

ver=getverticeshr(xkernel_12perday_trial1, percent=kernel.d_12perday_trial1[k])

for(i in 1:length(ver@polygons[[1]]@Polygons)){

polygon(ver@polygons[[1]]@Polygons[[i]]@coords, border=grey(level=0.25), col=NA, lty=ltys[k],lwd=lwds[k])

kernel.areas_12perday_trial1[k]=kernel.areas_12perday_trial1[k]+areapl(ver@polygons[[1]]@Polygons[[i]]@coords)/(1000^2)

}

}

kernel.areas_12perday_trial1

####

##Trial 2

####

set.seed(2)

tracks_12perday_trial2<-alltracks %>% group_by(yyyymmdd) %>% sample_n(size = 12)

##Preparing the coordinates for the calculations

utm_12perday_trial2=WGS.to.UTM(long=tracks_12perday_trial2$lon, lat=tracks_12perday_trial2$lat, utm.zone=utm.zone(mean(tracks_12perday_trial2$lon), mean(tracks_12perday_trial2$lat)), avoid.negatives=T, return.zone=F)

xy_12perday_trial2=SpatialPoints(coords=utm_12perday_trial2[, c("long", "lat")])

to.get=c(100, 99, 98, 95, 80, 75, 50)

MCP_12perday_trial2=vector("list", length(to.get))

names(MCP_12perday_trial2)=to.get

for(i in 1:length(to.get)){

xx_12perday_trial2=mcp(xy_12perday_trial2, percent=to.get[i])

MCP_12perday_trial2[[i]]=xx_12perday_trial2@polygons[[1]]@Polygons[[1]]@coords

}

MCP.areas_12perday_trial2=unlist(lapply(MCP_12perday_trial2, areapl))

MCP.areas_12perday_trial2=MCP.areas_12perday_trial2/(1000^2)

names(MCP.areas_12perday_trial2)=to.get

MCP.areas_12perday_trial2

k.data_12perday_trial2=SpatialPointsDataFrame(coords=utm_12perday_trial2, data=data.frame(id=rep("a", nrow(utm_12perday_trial2))))

xkernel_12perday_trial2=kernelUD(k.data_12perday_trial2, h="href", grid=100, extent=1)

##plot:

par(mar=rep(0.2, 4))

plot(as.matrix(MCP_12perday_trial2[["100"]]), asp=1, xlab="", ylab="",xlim=c(548350, 557873),ylim=c(9765030, 9774195), axes=T, type="n", xaxt="n", yaxt="n", bty="n")

cols=c("mediumaquamarine", "gray20", "gray30", "gray60","gray80", "gray90", "gray95")

for(i in 1:length(to.get)){

polygon(MCP_12perday_trial2[[i]], border=NA, col=cols[i])

}

for(i in 1:length(savannah)){

polygon(savannah[[i]], border=NA, col=adjustcolor(col="yellow", alpha.f=0.4))

}

polygon(swamp[[10]], border=NA, col=grey(level=0.4, alpha=0.4))

polygon(sea[[1]], border=NA, col="lightsteelblue2")

#add kernel:

kernel.d_12perday_trial2=rev(c(50, 75, 80, 95,98,99))

kernel.areas_12perday_trial2=rep(0, length(kernel.d_12perday_trial2))

names(kernel.areas_12perday_trial2)=kernel.d_12perday_trial2

ltys=c(1, 2,3,4,5,6)

lwds=c(1, 1,2,2,3,3)

for(k in 1:length(kernel.d_12perday_trial2)){

ver=getverticeshr(xkernel_12perday_trial2, percent=kernel.d_12perday_trial2[k])

for(i in 1:length(ver@polygons[[1]]@Polygons)){

polygon(ver@polygons[[1]]@Polygons[[i]]@coords, border=grey(level=0.25), col=NA, lty=ltys[k],lwd=lwds[k])

kernel.areas_12perday_trial2[k]=kernel.areas_12perday_trial2[k]+areapl(ver@polygons[[1]]@Polygons[[i]]@coords)/(1000^2)

}

}

kernel.areas_12perday_trial2

####

##Trial 3

####

set.seed(3)

tracks_12perday_trial3<-alltracks %>% group_by(yyyymmdd) %>% sample_n(size = 12)

##Preparing the coordinates for the calculations

utm_12perday_trial3=WGS.to.UTM(long=tracks_12perday_trial3$lon, lat=tracks_12perday_trial3$lat, utm.zone=utm.zone(mean(tracks_12perday_trial3$lon), mean(tracks_12perday_trial3$lat)), avoid.negatives=T, return.zone=F)

xy_12perday_trial3=SpatialPoints(coords=utm_12perday_trial3[, c("long", "lat")])

to.get=c(100, 99, 98, 95, 80, 75, 50)

MCP_12perday_trial3=vector("list", length(to.get))

names(MCP_12perday_trial3)=to.get

for(i in 1:length(to.get)){

xx_12perday_trial3=mcp(xy_12perday_trial3, percent=to.get[i])

MCP_12perday_trial3[[i]]=xx_12perday_trial3@polygons[[1]]@Polygons[[1]]@coords

}

MCP.areas_12perday_trial3=unlist(lapply(MCP_12perday_trial3, areapl))

MCP.areas_12perday_trial3=MCP.areas_12perday_trial3/(1000^2)

names(MCP.areas_12perday_trial3)=to.get

MCP.areas_12perday_trial3

k.data_12perday_trial3=SpatialPointsDataFrame(coords=utm_12perday_trial3, data=data.frame(id=rep("a", nrow(utm_12perday_trial3))))

xkernel_12perday_trial3=kernelUD(k.data_12perday_trial3, h="href", grid=100, extent=1)

##plot:

par(mar=rep(0.2, 4))

plot(as.matrix(MCP_12perday_trial3[["100"]]), asp=1, xlab="", ylab="",xlim=c(548350, 557873),ylim=c(9765030, 9774195), axes=T, type="n", xaxt="n", yaxt="n", bty="n")

cols=c("mediumaquamarine", "gray20", "gray30", "gray60","gray80", "gray90", "gray95")

for(i in 1:length(to.get)){

polygon(MCP_12perday_trial3[[i]], border=NA, col=cols[i])

}

for(i in 1:length(savannah)){

polygon(savannah[[i]], border=NA, col=adjustcolor(col="yellow", alpha.f=0.4))

}

polygon(swamp[[10]], border=NA, col=grey(level=0.4, alpha=0.4))

polygon(sea[[1]], border=NA, col="lightsteelblue2")

#add kernel:

kernel.d_12perday_trial3=rev(c(50, 75, 80, 95,98,99))

kernel.areas_12perday_trial3=rep(0, length(kernel.d_12perday_trial3))

names(kernel.areas_12perday_trial3)=kernel.d_12perday_trial3

ltys=c(1, 2,3,4,5,6)

lwds=c(1, 1,2,2,3,3)

for(k in 1:length(kernel.d_12perday_trial3)){

ver=getverticeshr(xkernel_12perday_trial3, percent=kernel.d_12perday_trial3[k])

for(i in 1:length(ver@polygons[[1]]@Polygons)){

polygon(ver@polygons[[1]]@Polygons[[i]]@coords, border=grey(level=0.25), col=NA, lty=ltys[k],lwd=lwds[k])

kernel.areas_12perday_trial3[k]=kernel.areas_12perday_trial3[k]+areapl(ver@polygons[[1]]@Polygons[[i]]@coords)/(1000^2)

}

}

kernel.areas_12perday_trial3

####

##Trial 4

####

set.seed(4)

tracks_12perday_trial4<-alltracks %>% group_by(yyyymmdd) %>% sample_n(size = 12)

##Preparing the coordinates for the calculations

utm_12perday_trial4=WGS.to.UTM(long=tracks_12perday_trial4$lon, lat=tracks_12perday_trial4$lat, utm.zone=utm.zone(mean(tracks_12perday_trial4$lon), mean(tracks_12perday_trial4$lat)), avoid.negatives=T, return.zone=F)

xy_12perday_trial4=SpatialPoints(coords=utm_12perday_trial4[, c("long", "lat")])

to.get=c(100, 99, 98, 95, 80, 75, 50)

MCP_12perday_trial4=vector("list", length(to.get))

names(MCP_12perday_trial4)=to.get

for(i in 1:length(to.get)){

xx_12perday_trial4=mcp(xy_12perday_trial4, percent=to.get[i])

MCP_12perday_trial4[[i]]=xx_12perday_trial4@polygons[[1]]@Polygons[[1]]@coords

}

MCP.areas_12perday_trial4=unlist(lapply(MCP_12perday_trial4, areapl))

MCP.areas_12perday_trial4=MCP.areas_12perday_trial4/(1000^2)

names(MCP.areas_12perday_trial4)=to.get

MCP.areas_12perday_trial4k.data_12perday_trial4=SpatialPointsDataFrame(coords=utm_12perday_trial4, data=data.frame(id=rep("a", nrow(utm_12perday_trial4))))

xkernel_12perday_trial4=kernelUD(k.data_12perday_trial4, h="href", grid=100, extent=1)

##plot:

par(mar=rep(0.2, 4))

plot(as.matrix(MCP_12perday_trial4[["100"]]), asp=1, xlab="", ylab="",xlim=c(548350, 557873),ylim=c(9765030, 9774195), axes=T, type="n", xaxt="n", yaxt="n", bty="n")

cols=c("mediumaquamarine", "gray20", "gray30", "gray60","gray80", "gray90", "gray95")

for(i in 1:length(to.get)){

polygon(MCP_12perday_trial4[[i]], border=NA, col=cols[i])

}

for(i in 1:length(savannah)){

polygon(savannah[[i]], border=NA, col=adjustcolor(col="yellow", alpha.f=0.4))

}

polygon(swamp[[10]], border=NA, col=grey(level=0.4, alpha=0.4))

polygon(sea[[1]], border=NA, col="lightsteelblue2")

#add kernel:

kernel.d_12perday_trial4=rev(c(50, 75, 80, 95,98,99))

kernel.areas_12perday_trial4=rep(0, length(kernel.d_12perday_trial4))

names(kernel.areas_12perday_trial4)=kernel.d_12perday_trial4

ltys=c(1, 2,3,4,5,6)

lwds=c(1, 1,2,2,3,3)

for(k in 1:length(kernel.d_12perday_trial4)){

ver=getverticeshr(xkernel_12perday_trial4, percent=kernel.d_12perday_trial4[k])

for(i in 1:length(ver@polygons[[1]]@Polygons)){

polygon(ver@polygons[[1]]@Polygons[[i]]@coords, border=grey(level=0.25), col=NA, lty=ltys[k],lwd=lwds[k])

kernel.areas_12perday_trial4[k]=kernel.areas_12perday_trial4[k]+areapl(ver@polygons[[1]]@Polygons[[i]]@coords)/(1000^2)

}

}

kernel.areas_12perday_trial4

####

##Trial 5

####

set.seed(5)

tracks_12perday_trial5<-alltracks %>% group_by(yyyymmdd) %>% sample_n(size = 12)

##Preparing the coordinates for the calculations

utm_12perday_trial5=WGS.to.UTM(long=tracks_12perday_trial5$lon, lat=tracks_12perday_trial5$lat, utm.zone=utm.zone(mean(tracks_12perday_trial5$lon), mean(tracks_12perday_trial5$lat)), avoid.negatives=T, return.zone=F)

xy_12perday_trial5=SpatialPoints(coords=utm_12perday_trial5[, c("long", "lat")])

to.get=c(100, 99, 98, 95, 80, 75, 50)

MCP_12perday_trial5=vector("list", length(to.get))

names(MCP_12perday_trial5)=to.get

for(i in 1:length(to.get)){

xx_12perday_trial5=mcp(xy_12perday_trial5, percent=to.get[i])

MCP_12perday_trial5[[i]]=xx_12perday_trial5@polygons[[1]]@Polygons[[1]]@coords

}

MCP.areas_12perday_trial5=unlist(lapply(MCP_12perday_trial5, areapl))

MCP.areas_12perday_trial5=MCP.areas_12perday_trial5/(1000^2)

names(MCP.areas_12perday_trial5)=to.get

MCP.areas_12perday_trial5

k.data_12perday_trial5=SpatialPointsDataFrame(coords=utm_12perday_trial5, data=data.frame(id=rep("a", nrow(utm_12perday_trial5))))

xkernel_12perday_trial5=kernelUD(k.data_12perday_trial5, h="href", grid=100, extent=1)

##plot:

par(mar=rep(0.2, 4))

plot(as.matrix(MCP_12perday_trial5[["100"]]), asp=1, xlab="", ylab="",xlim=c(548350, 557873),ylim=c(9765030, 9774195), axes=T, type="n", xaxt="n", yaxt="n", bty="n")

cols=c("mediumaquamarine", "gray20", "gray30", "gray60","gray80", "gray90", "gray95")

for(i in 1:length(to.get)){

polygon(MCP_12perday_trial5[[i]], border=NA, col=cols[i])

}

for(i in 1:length(savannah)){

polygon(savannah[[i]], border=NA, col=adjustcolor(col="yellow", alpha.f=0.4))

}

polygon(swamp[[10]], border=NA, col=grey(level=0.4, alpha=0.4))

polygon(sea[[1]], border=NA, col="lightsteelblue2")

#add kernel:

kernel.d_12perday_trial5=rev(c(50, 75, 80, 95,98,99))

kernel.areas_12perday_trial5=rep(0, length(kernel.d_12perday_trial5))

names(kernel.areas_12perday_trial5)=kernel.d_12perday_trial5

ltys=c(1, 2,3,4,5,6)

lwds=c(1, 1,2,2,3,3)

for(k in 1:length(kernel.d_12perday_trial5)){

ver=getverticeshr(xkernel_12perday_trial5, percent=kernel.d_12perday_trial5[k])

for(i in 1:length(ver@polygons[[1]]@Polygons)){

polygon(ver@polygons[[1]]@Polygons[[i]]@coords, border=grey(level=0.25), col=NA, lty=ltys[k],lwd=lwds[k])

kernel.areas_12perday_trial5[k]=kernel.areas_12perday_trial5[k]+areapl(ver@polygons[[1]]@Polygons[[i]]@coords)/(1000^2)

}

}

kernel.areas_12perday_trial5

####

##Trial 6

####

set.seed(6)

tracks_12perday_trial6<-alltracks %>% group_by(yyyymmdd) %>% sample_n(size = 12)

##Preparing the coordinates for the calculations

utm_12perday_trial6=WGS.to.UTM(long=tracks_12perday_trial6$lon, lat=tracks_12perday_trial6$lat, utm.zone=utm.zone(mean(tracks_12perday_trial6$lon), mean(tracks_12perday_trial6$lat)), avoid.negatives=T, return.zone=F)

xy_12perday_trial6=SpatialPoints(coords=utm_12perday_trial6[, c("long", "lat")])

to.get=c(100, 99, 98, 95, 80, 75, 50)

MCP_12perday_trial6=vector("list", length(to.get))

names(MCP_12perday_trial6)=to.get

for(i in 1:length(to.get)){

xx_12perday_trial6=mcp(xy_12perday_trial6, percent=to.get[i])

MCP_12perday_trial6[[i]]=xx_12perday_trial6@polygons[[1]]@Polygons[[1]]@coords

}

MCP.areas_12perday_trial6=unlist(lapply(MCP_12perday_trial6, areapl))

MCP.areas_12perday_trial6=MCP.areas_12perday_trial6/(1000^2)

names(MCP.areas_12perday_trial6)=to.get

MCP.areas_12perday_trial6

k.data_12perday_trial6=SpatialPointsDataFrame(coords=utm_12perday_trial6, data=data.frame(id=rep("a", nrow(utm_12perday_trial6))))

xkernel_12perday_trial6=kernelUD(k.data_12perday_trial6, h="href", grid=100, extent=1)

##plot:

par(mar=rep(0.2, 4))

plot(as.matrix(MCP_12perday_trial6[["100"]]), asp=1, xlab="", ylab="",xlim=c(548350, 557873),ylim=c(9765030, 9774195), axes=T, type="n", xaxt="n", yaxt="n", bty="n")

cols=c("mediumaquamarine", "gray20", "gray30", "gray60","gray80", "gray90", "gray95")

for(i in 1:length(to.get)){

polygon(MCP_12perday_trial6[[i]], border=NA, col=cols[i])

}

for(i in 1:length(savannah)){

polygon(savannah[[i]], border=NA, col=adjustcolor(col="yellow", alpha.f=0.4))

}

polygon(swamp[[10]], border=NA, col=grey(level=0.4, alpha=0.4))

polygon(sea[[1]], border=NA, col="lightsteelblue2")

#add kernel:

kernel.d_12perday_trial6=rev(c(50, 75, 80, 95,98,99))

kernel.areas_12perday_trial6=rep(0, length(kernel.d_12perday_trial6))

names(kernel.areas_12perday_trial6)=kernel.d_12perday_trial6

ltys=c(1, 2,3,4,5,6)

lwds=c(1, 1,2,2,3,3)

for(k in 1:length(kernel.d_12perday_trial6)){

ver=getverticeshr(xkernel_12perday_trial6, percent=kernel.d_12perday_trial6[k])

for(i in 1:length(ver@polygons[[1]]@Polygons)){

polygon(ver@polygons[[1]]@Polygons[[i]]@coords, border=grey(level=0.25), col=NA, lty=ltys[k],lwd=lwds[k])

kernel.areas_12perday_trial6[k]=kernel.areas_12perday_trial6[k]+areapl(ver@polygons[[1]]@Polygons[[i]]@coords)/(1000^2)

}

}

kernel.areas_12perday_trial6

####

##Trial 7

####

set.seed(7)

tracks_12perday_trial7<-alltracks %>% group_by(yyyymmdd) %>% sample_n(size = 12)

##Preparing the coordinates for the calculations

utm_12perday_trial7=WGS.to.UTM(long=tracks_12perday_trial7$lon, lat=tracks_12perday_trial7$lat, utm.zone=utm.zone(mean(tracks_12perday_trial7$lon), mean(tracks_12perday_trial7$lat)), avoid.negatives=T, return.zone=F)

xy_12perday_trial7=SpatialPoints(coords=utm_12perday_trial7[, c("long", "lat")])

to.get=c(100, 99, 98, 95, 80, 75, 50)

MCP_12perday_trial7=vector("list", length(to.get))

names(MCP_12perday_trial7)=to.get

for(i in 1:length(to.get)){

xx_12perday_trial7=mcp(xy_12perday_trial7, percent=to.get[i])

MCP_12perday_trial7[[i]]=xx_12perday_trial7@polygons[[1]]@Polygons[[1]]@coords

}

MCP.areas_12perday_trial7=unlist(lapply(MCP_12perday_trial7, areapl))

MCP.areas_12perday_trial7=MCP.areas_12perday_trial7/(1000^2)

names(MCP.areas_12perday_trial7)=to.get

MCP.areas_12perday_trial7

k.data_12perday_trial7=SpatialPointsDataFrame(coords=utm_12perday_trial7, data=data.frame(id=rep("a", nrow(utm_12perday_trial7))))

xkernel_12perday_trial7=kernelUD(k.data_12perday_trial7, h="href", grid=100, extent=1)

##plot:

par(mar=rep(0.2, 4))

plot(as.matrix(MCP_12perday_trial7[["100"]]), asp=1, xlab="", ylab="",xlim=c(548350, 557873),ylim=c(9765030, 9774195), axes=T, type="n", xaxt="n", yaxt="n", bty="n")

cols=c("mediumaquamarine", "gray20", "gray30", "gray60","gray80", "gray90", "gray95")

for(i in 1:length(to.get)){

polygon(MCP_12perday_trial7[[i]], border=NA, col=cols[i])

}

for(i in 1:length(savannah)){

polygon(savannah[[i]], border=NA, col=adjustcolor(col="yellow", alpha.f=0.4))

}

polygon(swamp[[10]], border=NA, col=grey(level=0.4, alpha=0.4))

polygon(sea[[1]], border=NA, col="lightsteelblue2")

#add kernel:

kernel.d_12perday_trial7=rev(c(50, 75, 80, 95,98,99))

kernel.areas_12perday_trial7=rep(0, length(kernel.d_12perday_trial7))

names(kernel.areas_12perday_trial7)=kernel.d_12perday_trial7

ltys=c(1, 2,3,4,5,6)

lwds=c(1, 1,2,2,3,3)

for(k in 1:length(kernel.d_12perday_trial7)){

ver=getverticeshr(xkernel_12perday_trial7, percent=kernel.d_12perday_trial7[k])

for(i in 1:length(ver@polygons[[1]]@Polygons)){

polygon(ver@polygons[[1]]@Polygons[[i]]@coords, border=grey(level=0.25), col=NA, lty=ltys[k],lwd=lwds[k])

kernel.areas_12perday_trial7[k]=kernel.areas_12perday_trial7[k]+areapl(ver@polygons[[1]]@Polygons[[i]]@coords)/(1000^2)

}

}

kernel.areas_12perday_trial7

####

##Trial 8

####

set.seed(8)

tracks_12perday_trial8<-alltracks %>% group_by(yyyymmdd) %>% sample_n(size = 12)

##Preparing the coordinates for the calculations

utm_12perday_trial8=WGS.to.UTM(long=tracks_12perday_trial8$lon, lat=tracks_12perday_trial8$lat, utm.zone=utm.zone(mean(tracks_12perday_trial8$lon), mean(tracks_12perday_trial8$lat)), avoid.negatives=T, return.zone=F)

xy_12perday_trial8=SpatialPoints(coords=utm_12perday_trial8[, c("long", "lat")])

to.get=c(100, 99, 98, 95, 80, 75, 50)

MCP_12perday_trial8=vector("list", length(to.get))

names(MCP_12perday_trial8)=to.get

for(i in 1:length(to.get)){

xx_12perday_trial8=mcp(xy_12perday_trial8, percent=to.get[i])

MCP_12perday_trial8[[i]]=xx_12perday_trial8@polygons[[1]]@Polygons[[1]]@coords

}

MCP.areas_12perday_trial8=unlist(lapply(MCP_12perday_trial8, areapl))

MCP.areas_12perday_trial8=MCP.areas_12perday_trial8/(1000^2)

names(MCP.areas_12perday_trial8)=to.get

MCP.areas_12perday_trial8k.data_12perday_trial8=SpatialPointsDataFrame(coords=utm_12perday_trial8, data=data.frame(id=rep("a", nrow(utm_12perday_trial8))))

xkernel_12perday_trial8=kernelUD(k.data_12perday_trial8, h="href", grid=100, extent=1)

##plot:

par(mar=rep(0.2, 4))

plot(as.matrix(MCP_12perday_trial8[["100"]]), asp=1, xlab="", ylab="",xlim=c(548350, 557873),ylim=c(9765030, 9774195), axes=T, type="n", xaxt="n", yaxt="n", bty="n")

cols=c("mediumaquamarine", "gray20", "gray30", "gray60","gray80", "gray90", "gray95")

for(i in 1:length(to.get)){

polygon(MCP_12perday_trial8[[i]], border=NA, col=cols[i])

}

for(i in 1:length(savannah)){

polygon(savannah[[i]], border=NA, col=adjustcolor(col="yellow", alpha.f=0.4))

}

polygon(swamp[[10]], border=NA, col=grey(level=0.4, alpha=0.4))

polygon(sea[[1]], border=NA, col="lightsteelblue2")

#add kernel:

kernel.d_12perday_trial8=rev(c(50, 75, 80, 95,98,99))

kernel.areas_12perday_trial8=rep(0, length(kernel.d_12perday_trial8))

names(kernel.areas_12perday_trial8)=kernel.d_12perday_trial8

ltys=c(1, 2,3,4,5,6)

lwds=c(1, 1,2,2,3,3)

for(k in 1:length(kernel.d_12perday_trial8)){

ver=getverticeshr(xkernel_12perday_trial8, percent=kernel.d_12perday_trial8[k])

for(i in 1:length(ver@polygons[[1]]@Polygons)){

polygon(ver@polygons[[1]]@Polygons[[i]]@coords, border=grey(level=0.25), col=NA, lty=ltys[k],lwd=lwds[k])

kernel.areas_12perday_trial8[k]=kernel.areas_12perday_trial8[k]+areapl(ver@polygons[[1]]@Polygons[[i]]@coords)/(1000^2)

}

}

kernel.areas_12perday_trial8

####

##Trial 9

####

set.seed(9)

tracks_12perday_trial9<-alltracks %>% group_by(yyyymmdd) %>% sample_n(size = 12)

##Preparing the coordinates for the calculations

utm_12perday_trial9=WGS.to.UTM(long=tracks_12perday_trial9$lon, lat=tracks_12perday_trial9$lat, utm.zone=utm.zone(mean(tracks_12perday_trial9$lon), mean(tracks_12perday_trial9$lat)), avoid.negatives=T, return.zone=F)

xy_12perday_trial9=SpatialPoints(coords=utm_12perday_trial9[, c("long", "lat")])

to.get=c(100, 99, 98, 95, 80, 75, 50)

MCP_12perday_trial9=vector("list", length(to.get))

names(MCP_12perday_trial9)=to.get

for(i in 1:length(to.get)){

xx_12perday_trial9=mcp(xy_12perday_trial9, percent=to.get[i])

MCP_12perday_trial9[[i]]=xx_12perday_trial9@polygons[[1]]@Polygons[[1]]@coords

}

MCP.areas_12perday_trial9=unlist(lapply(MCP_12perday_trial9, areapl))

MCP.areas_12perday_trial9=MCP.areas_12perday_trial9/(1000^2)

names(MCP.areas_12perday_trial9)=to.get

MCP.areas_12perday_trial9

k.data_12perday_trial9=SpatialPointsDataFrame(coords=utm_12perday_trial9, data=data.frame(id=rep("a", nrow(utm_12perday_trial9))))

xkernel_12perday_trial9=kernelUD(k.data_12perday_trial9, h="href", grid=100, extent=1)

##plot:

par(mar=rep(0.2, 4))

plot(as.matrix(MCP_12perday_trial9[["100"]]), asp=1, xlab="", ylab="",xlim=c(548350, 557873),ylim=c(9765030, 9774195), axes=T, type="n", xaxt="n", yaxt="n", bty="n")

cols=c("mediumaquamarine", "gray20", "gray30", "gray60","gray80", "gray90", "gray95")

for(i in 1:length(to.get)){

polygon(MCP_12perday_trial9[[i]], border=NA, col=cols[i])

}

for(i in 1:length(savannah)){

polygon(savannah[[i]], border=NA, col=adjustcolor(col="yellow", alpha.f=0.4))

}

polygon(swamp[[10]], border=NA, col=grey(level=0.4, alpha=0.4))

polygon(sea[[1]], border=NA, col="lightsteelblue2")

#add kernel:

kernel.d_12perday_trial9=rev(c(50, 75, 80, 95,98,99))

kernel.areas_12perday_trial9=rep(0, length(kernel.d_12perday_trial9))

names(kernel.areas_12perday_trial9)=kernel.d_12perday_trial9

ltys=c(1, 2,3,4,5,6)

lwds=c(1, 1,2,2,3,3)

for(k in 1:length(kernel.d_12perday_trial9)){

ver=getverticeshr(xkernel_12perday_trial9, percent=kernel.d_12perday_trial9[k])

for(i in 1:length(ver@polygons[[1]]@Polygons)){

polygon(ver@polygons[[1]]@Polygons[[i]]@coords, border=grey(level=0.25), col=NA, lty=ltys[k],lwd=lwds[k])

kernel.areas_12perday_trial9[k]=kernel.areas_12perday_trial9[k]+areapl(ver@polygons[[1]]@Polygons[[i]]@coords)/(1000^2)

}

}

kernel.areas_12perday_trial9

####

##Trial 10

####

set.seed(10)

tracks_12perday_trial10<-alltracks %>% group_by(yyyymmdd) %>% sample_n(size = 12)

##Preparing the coordinates for the calculations

utm_12perday_trial10=WGS.to.UTM(long=tracks_12perday_trial10$lon, lat=tracks_12perday_trial10$lat, utm.zone=utm.zone(mean(tracks_12perday_trial10$lon), mean(tracks_12perday_trial10$lat)), avoid.negatives=T, return.zone=F)

xy_12perday_trial10=SpatialPoints(coords=utm_12perday_trial10[, c("long", "lat")])

to.get=c(100, 99, 98, 95, 80, 75, 50)

MCP_12perday_trial10=vector("list", length(to.get))

names(MCP_12perday_trial10)=to.get

for(i in 1:length(to.get)){

xx_12perday_trial10=mcp(xy_12perday_trial10, percent=to.get[i])

MCP_12perday_trial10[[i]]=xx_12perday_trial10@polygons[[1]]@Polygons[[1]]@coords

}

MCP.areas_12perday_trial10=unlist(lapply(MCP_12perday_trial10, areapl))

MCP.areas_12perday_trial10=MCP.areas_12perday_trial10/(1000^2)

names(MCP.areas_12perday_trial10)=to.get

MCP.areas_12perday_trial10

k.data_12perday_trial10=SpatialPointsDataFrame(coords=utm_12perday_trial10, data=data.frame(id=rep("a", nrow(utm_12perday_trial10))))

xkernel_12perday_trial10=kernelUD(k.data_12perday_trial10, h="href", grid=100, extent=1)

##plot:

par(mar=rep(0.2, 4))

plot(as.matrix(MCP_12perday_trial10[["100"]]), asp=1, xlab="", ylab="",xlim=c(548350, 557873),ylim=c(9765030, 9774195), axes=T, type="n", xaxt="n", yaxt="n", bty="n")

cols=c("mediumaquamarine", "gray20", "gray30", "gray60","gray80", "gray90", "gray95")

for(i in 1:length(to.get)){

polygon(MCP_12perday_trial10[[i]], border=NA, col=cols[i])

}

for(i in 1:length(savannah)){

polygon(savannah[[i]], border=NA, col=adjustcolor(col="yellow", alpha.f=0.4))

}

polygon(swamp[[10]], border=NA, col=grey(level=0.4, alpha=0.4))

polygon(sea[[1]], border=NA, col="lightsteelblue2")

#add kernel:

kernel.d_12perday_trial10=rev(c(50, 75, 80, 95,98,99))

kernel.areas_12perday_trial10=rep(0, length(kernel.d_12perday_trial10))

names(kernel.areas_12perday_trial10)=kernel.d_12perday_trial10

ltys=c(1, 2,3,4,5,6)

lwds=c(1, 1,2,2,3,3)

for(k in 1:length(kernel.d_12perday_trial10)){

ver=getverticeshr(xkernel_12perday_trial10, percent=kernel.d_12perday_trial10[k])

for(i in 1:length(ver@polygons[[1]]@Polygons)){

polygon(ver@polygons[[1]]@Polygons[[i]]@coords, border=grey(level=0.25), col=NA, lty=ltys[k],lwd=lwds[k])

kernel.areas_12perday_trial10[k]=kernel.areas_12perday_trial10[k]+areapl(ver@polygons[[1]]@Polygons[[i]]@coords)/(1000^2)

}

}

kernel.areas_12perday_trial10

########################

# 10.5 Comparing KDE across the three different subsampling schemes

########################

kernel_areas<-as.data.frame(

rbind(kernel.areas_1perday_trial1,

kernel.areas_1perday_trial2,

kernel.areas_1perday_trial3,

kernel.areas_1perday_trial4,

kernel.areas_1perday_trial5,

kernel.areas_1perday_trial6,

kernel.areas_1perday_trial7,

kernel.areas_1perday_trial8,

kernel.areas_1perday_trial9,

kernel.areas_1perday_trial10,

kernel.areas_3perday_trial1,

kernel.areas_3perday_trial2,

kernel.areas_3perday_trial3,

kernel.areas_3perday_trial4,

kernel.areas_3perday_trial5,

kernel.areas_3perday_trial6,

kernel.areas_3perday_trial7,

kernel.areas_3perday_trial8,

kernel.areas_3perday_trial9,

kernel.areas_3perday_trial10,

kernel.areas_12perday_trial1,

kernel.areas_12perday_trial2,

kernel.areas_12perday_trial3,

kernel.areas_12perday_trial4,

kernel.areas_12perday_trial5,

kernel.areas_12perday_trial6,

kernel.areas_12perday_trial7,

kernel.areas_12perday_trial8,

kernel.areas_12perday_trial9,

kernel.areas_12perday_trial10))

n_samples<-c(rep(1, 10),rep(3, 10),rep(12, 10))

kernel_areas<-cbind(kernel_areas,n_samples)

kernel_areas

kernel_areas$n_samples <- ordered(kernel_areas$n_samples,

levels = c("1", "3", "12"))

levels(kernel_areas$n_samples)

kernel_areas<-kernel_areas%>% rename( "p50"= "50","p95"= "95")

tapply(kernel_areas$p95,kernel_areas$n_samples, mean)

tapply(kernel_areas$p95,kernel_areas$n_samples, sd)

tapply(kernel_areas$p50,kernel_areas$n_samples, mean)

tapply(kernel_areas$p50,kernel_areas$n_samples, sd)

install.packages("ggpubr")#to plot boxplot

install.packages("car")#to compute Levene test (Homogeinity variances)

library("ggpubr")

library(car)

##Contrast between 95%KDE

leveneTest(p95~ n_samples, data = kernel_areas)

res.aov <- aov(p95~ n_samples, data = kernel_areas)

summary(res.aov)

TukeyHSD(res.aov)

plot(res.aov, 2)

ggboxplot(kernel_areas, x = "n_samples", y = "p95",

color = "n_samples", palette = c("#00AFBB", "#E7B800", "#FC4E07"),

order = c("1", "3", "12"),

ylab = "Km2 (KDE95) ", xlab = "No.relocations per day")

##Contrast between 50%KDE

leveneTest(p50~ n_samples, data = kernel_areas)

res.aov <- aov(p50~ n_samples, data = kernel_areas)

summary(res.aov)

TukeyHSD(res.aov)

plot(res.aov, 2)

ggboxplot(kernel_areas, x = "n_samples", y = "p50",

color = "n_samples", palette = c("#00AFBB", "#E7B800", "#FC4E07"),

order = c("1", "3", "12"),

ylab = "Km2 (KDE50) ", xlab = "No.relocations per day")

########################

# 10.6 Comparing MPC across the three different subsampling schemes

########################

MCP_areas<-as.data.frame(

rbind(MCP.areas_1perday_trial1,

MCP.areas_1perday_trial2,

MCP.areas_1perday_trial3,

MCP.areas_1perday_trial4,

MCP.areas_1perday_trial5,

MCP.areas_1perday_trial6,

MCP.areas_1perday_trial7,

MCP.areas_1perday_trial8,

MCP.areas_1perday_trial9,

MCP.areas_1perday_trial10,

MCP.areas_3perday_trial1,

MCP.areas_3perday_trial2,

MCP.areas_3perday_trial3,

MCP.areas_3perday_trial4,

MCP.areas_3perday_trial5,

MCP.areas_3perday_trial6,

MCP.areas_3perday_trial7,

MCP.areas_3perday_trial8,

MCP.areas_3perday_trial9,

MCP.areas_3perday_trial10,

MCP.areas_12perday_trial1,

MCP.areas_12perday_trial2,

MCP.areas_12perday_trial3,

MCP.areas_12perday_trial4,

MCP.areas_12perday_trial5,

MCP.areas_12perday_trial6,

MCP.areas_12perday_trial7,

MCP.areas_12perday_trial8,

MCP.areas_12perday_trial9,

MCP.areas_12perday_trial10))

MCP_areas<-cbind(MCP_areas,n_samples)

MCP_areas$n_samples <- ordered(MCP_areas$n_samples,

levels = c("1", "3", "12"))

levels(MCP_areas$n_samples)

MCP_areas<-MCP_areas%>% rename( "p50"= "50","p100"= "100")

tapply(MCP_areas$p100,MCP_areas$n_samples, mean)

tapply(MCP_areas$p100,MCP_areas$n_samples, sd)

tapply(MCP_areas$p50,MCP_areas$n_samples, mean)

tapply(MCP_areas$p50,MCP_areas$n_samples, sd)

MCPareas1perday<-MCP_areas[MCP_areas$n_samples==1,]

summary(MCPareas1perday)

##Contrast between 100%MCP

leveneTest(p100~ n_samples, data = MCP_areas)

res.aov <- aov(p100~ n_samples, data = MCP_areas)

summary(res.aov)

TukeyHSD(res.aov)

plot(res.aov, 2)

ggboxplot(MCP_areas, x = "n_samples", y = "p100",

color = "n_samples", palette = c("#00AFBB", "#E7B800", "#FC4E07"),

order = c("1", "3", "12"),

ylab = "Km2 (MCP100) ", xlab = "No.relocations per day")

##Contrast between 50%MCP

leveneTest(p50~ n_samples, data = MCP_areas)

res.aov <- aov(p50~ n_samples, data = MCP_areas)

summary(res.aov)

TukeyHSD(res.aov)

plot(res.aov, 2)

ggboxplot(MCP_areas, x = "n_samples", y = "p50",

color = "n_samples", palette = c("#00AFBB", "#E7B800", "#FC4E07"),

order = c("1", "3", "12"),

ylab = "Km2 (MCP50) ", xlab = "No.relocations per day")
